# Consequences of different sample drying temperatures for accuracy of biomass inventories in forest ecosystems

**DOI:** 10.1038/s41598-020-73053-9

**Published:** 2020-09-29

**Authors:** Andrzej M. Jagodziński, Marcin K. Dyderski, Kamil Gęsikiewicz, Paweł Horodecki

**Affiliations:** Institute of Dendrology, Polish Academy of Sciences, Parkowa 5, 62-035 Kórnik, Poland

**Keywords:** Ecology, Ecology

## Abstract

Biomass estimation is one of the crucial tasks of forest ecology. Drying tree material is a crucial stage of preparing biomass estimation tools. However, at this step researchers use different drying temperatures, but we do not know how this influences accuracy of models. We aimed to assess differences in dry biomass between two drying temperatures (75 °C and 105 °C) in tree biomass components and to provide coefficients allowing for recalculation between the given temperatures. We used a set of 1440 samples from bark, branches, foliage and wood of eight European tree species: *Abies alba* Mill., *Alnus glutinosa* (L.) Gaertn., *Betula pendula* Roth., *Fagus sylvatica* L., *Larix decidua* Mill., *Picea abies* (L.) H. Karst., *Pinus sylvestris* L. and *Quercus robur* L. The differences between drying temperatures were 1.67%, 1.76%, 2.20% and 0.96% of sample dry masses of bark, branches, foliage and stem wood, respectively. Tree species influenced these differences. Our study provided coefficients allowing for recalculation of masses between the two temperatures, to unify results from different studies. However, the difference in dry mass between the two temperatures studied is lower than the range of uncertainty of biomass models, thus its influence on results of large-scale biomass assessments is low.

## Introduction

Due to increasing carbon dioxide concentration in the atmosphere and increasing problems with climate change, researchers are focusing on assessment of forest capacity for carbon storage^1–3^. Due to high carbon content in woody biomass, reforestation and afforestation are considered as strategies of climate change mitigation, together with bioenergy carbon capture and storage (e.g. biochar), wetland restoration and construction, direct air carbon capture and storage, and enhanced terrestrial weathering^4^. However, afforestation cannot be considered as a holy grail, because the cooling effects of forests are connected not only with carbon accumulation, but also with canopy albedo and summertime atmospheric boundary layer temperature change by species conversion^5^. Therefore, forest management aiming to mitigate climate change has a trade-off between maximizing carbon sinks and reducing air temperatures^5^. Also, permanence of carbon storage in forest ecosystems is vulnerable to natural and human disturbances, especially fire, pests and wind^6^.

Another uses of tree biomass in climate change mitigation covers usage of biochar–biomass pyrolytically converted into charcoal, and biomass combustion instead of fossil fuels. Both methods are also sources of CO_2_, and also other greenhouse gases emissions, such as NO_x_^7^. Application of biochar increases the time of carbon immobilization and subsequently can increase crop yields^8,9^. In contrast, biomass combustion substitutes long-term stored carbon from fossil fuels with short-term stored carbon in biomass. This solution does not capture and immobilize carbon. In contrast to biochar, biomass combustion might result in the increase of atmospheric CO_2_ concentration in a long-term perspective^10^.

Forest ecosystems are one of the most important carbon sinks, estimated to be globally about. 2.4 ± 0.4 Pg C per year^11^. Due to relatively constant carbon content within species and tree components^12–14^, assessment of carbon pools is based on the variability of biomass in forest ecosystems. This variability is connected with tree stand parameters, especially those shaping trees dimensions (age, stand density and volume), as well as with tree species, shaping wood density. Thus, biomass estimation may be conducted on tree- and stand-levels^15–17^.

Regardless of the method applied, assessments of biomass rely on statistical models, highly dependent on the quality of samples collected in the field and processed in the laboratory. As harvest of sample trees is time and money consuming^18^, researchers usually tend to use one dataset for many purposes. For that reason samples are not dried at the temperature of 105 °C, provided in numerous guidelines^18^. Lower drying temperature is usually connected with the need to use one sample for other analyses, e.g. nitrogen content. Thus, a brief literature review revealed that biomass components were dried at different temperatures, including 60 °C^19–21^, 65 °C^22–24^, 70 °C^25–27^, 75 °C^17,28,29^, 85 °C^30,31^ and 105 °C^32–34^.

Water in wood is stored in two different states. Bound water is present in cell walls, whereas free water is present in the cell lumen and other void spaces^35^. Moreover, at higher temperatures some of the volatile organic compounds evaporate, as their boiling temperatures are lower than 105 °C. Depending on material type, these compounds may constitute from 0.004% (hard wood) to 1.74% (pitchy wood) of dry matter after drying at 105 °C^36^. Despite the wide range of temperatures used in biomass studies, there are no comprehensive estimates of the difference in biomass obtained at different drying temperatures. Samuelsson et al.^37^ compared moisture content in 20 different biomass materials, including drying at three temperatures (80, 105 and 130 °C). However, the differences revealed between drying temperatures were based on small sample sizes (in almost all cases ten samples dried at 105 °C and three samples dried at other temperatures). Thus, up to this time there are no comprehensive estimates of differences in biomass at different drying temperatures. Consequently we are not certain how much these differences influence estimates of carbon pools. There are also no calculation coefficients allowing recalculation of results obtained by different authors. Hence, we aimed to assess differences in dry biomass between two drying temperatures (75 °C and 105 °C) in tree biomass components (Table 1), and to evaluate the impacts of these changes on biomass assessment using published data on biomass stock, at the levels of tree, stand and country. We hypothesized that (1) differences in dry biomass between drying temperatures will differ among tree species and will be sample mass-dependent and (2) these differences will be highest in foliage and fine branches, as these components have higher moisture content than bark and wood.

**Table 1:** Overview of tree species studied and their biological traits.

Species	Type of wood	Wood density^1^ [Mg m^−3^]	Leaf area^2^ [mm^2^]	Volume in Europe^3^ [10^6^ m^3^]
*Abies alba*	Coniferous	0.353	41.25	694.4
*Alnus glutinosa*	Diffuse porous	0.420	3462.00	622.2
*Betula pendula*	Diffuse porous	0.513	1181.10	1802.0
*Fagus sylvatica*	Diffuse porous	0.585	2027.68	2320.6
*Larix decidua*	Coniferous	0.474	20.13	287.9
*Picea abies*	Coniferous	0.370	–	6624.6
*Pinus sylvestris*	Coniferous	0.395	65.94	7430.1
*Quercus robur*	Ring porous	0.575	2073.53	2289.6

Sources: 1—Wood density database^48,51^, 2—LEDA traits database^52^, no data for *P. abies* leaf area; 3—total volume of tree stand according to the FAO Global Resources Assessment in 2010^53^—data for countries in temperate and boreal climate, for *Alnus*, *Betula*, *Larix* and *Quercus* aggregated at the genera level.

## Results

**Figure 1:**
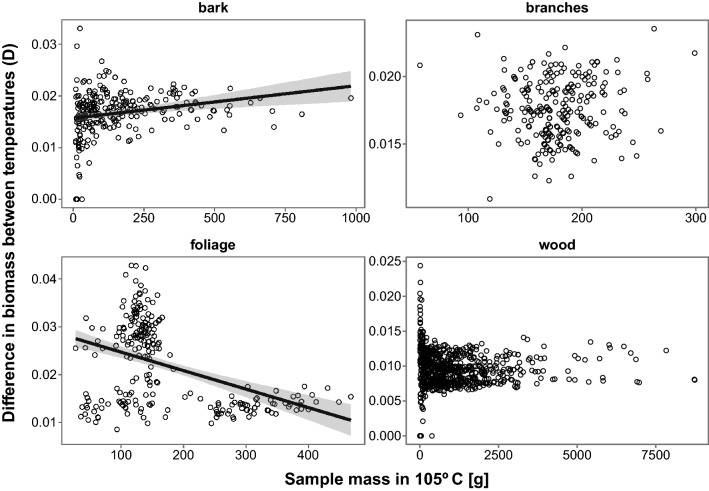
Relationships between sample mass (at 105 °C) and difference between drying temperatures (D) for biomass components (dimensionless). Regression lines were drawn for components with significant influence of sample mass (Table 2, S3). The plot has been generated using R software^38^.

The mean difference between dry masses obtained at different drying temperatures were highest for foliage (2.20 ± 0.06%) and lowest for wood (0.96 ± 0.01%). For bark and branches the mean differences were 1.67 ± 0.03% and 1.76 ± 0.01%, respectively. Models of differences between drying temperatures revealed statistically significant impacts of tree species for each component studied (Table 2, S3). In the cases of bark and foliage we also found statistically significant influences of sample mass (Fig. 1). However, for foliage higher values of sample mass occurred only for two species (*A. alba* and *P. abies*). For bark samples fixed effects explained 20.7% of the variability in D and both random and fixed effects explained 26.7%. The highest D was in *P. abies* and *P. sylvestris* and the lowest in *B. pendula* (Table 3). Fixed effects in the model of D for branches explained 61.0% of variability whereas both random and fixed effects explained 64.4%. The highest D was found in *L. decidua* and the lowest in *B. pendula*. In the case of foliage, fixed effects explained 88.8% of the variability whereas both random and fixed effects explained 89.8%. We found the highest D in *A. glutinosa* while the lowest was in *P. abies*, *A. alba* and *P. sylvestris*. Fixed effects in the model of D in wood explained 88.7% of the variability whereas both random and fixed effects explained 89.8%. The highest D was in *A. alba* and *Q. robur* and the lowest in *A. glutinosa*, *B. pendula* and *F. sylvatica*.

**Table 2:** Results of mixed effects ANCOVA explaining impacts of the species studied and sample masses on the differences in dry mass between two drying temperatures (D).

Component	Term	Sum of squares	Mean square	Numerator df	Denominator df	F value	Pr( >|F|)
Bark	Species	0.00066	0.00009	7	47.625	5.615	0.0001
Bark	Sample mass	0.00015	0.00015	1	186.199	9.032	0.0030
Branches	Species	0.00049	0.00007	7	42.705	39.081	< 0.0001
Branches	Sample mass	< 0.00001	< 0.00001	1	154.639	0.004	0.9509
Foliage	Species	0.00932	0.00133	7	54.757	170.260	< 0.0001
Foliage	Sample mass	0.00004	0.00004	1	205.419	5.720	0.0177
Wood	Species	0.00133	0.00019	7	76.214	53.259	< 0.0001
Wood	Sample mass	0.00001	0.00001	1	554.701	3.159	0.0761

**Table 3:** Mean and SE of difference in dry mass between two drying temperatures (D) for each biomass component and species. Species which did not differ statistically significantly (p > 0.05 according to Tukey’s *posteriori* test) within each component are marked with the same letter.

Species	Bark (n = 30)	SE	Branches (n = 30)	SE	Foliage (n = 30)	SE	Wood (n = 90)	SE
*Abies alba*	0.01720	0.00036ac	0.01705	0.00019bc	0.01314	0.00025b	0.01195	0.00011e
*Alnus glutinosa*	0.01627	0.00074ac	0.01623	0.00026b	0.03375	0.00089d	0.00760	0.00014a
*Betula pendula*	0.01327	0.00105a	0.01493	0.00023a	0.02918	0.00065c	0.00774	0.00013a
*Fagus sylvatica*	0.01434	0.00104ab	0.01598	0.00022ab	0.02826	0.00063c	0.00785	0.00015a
*Larix decidua*	0.01848	0.00040bc	0.02007	0.00022e	0.01608	0.00048a	0.00960	0.00015b
*Picea abies*	0.01884	0.00096c	0.01949	0.00019e	0.01386	0.00033b	0.01082	0.00029cd
*Pinus sylvestris*	0.01889	0.00073c	0.01816	0.00030cd	0.01275	0.00030ab	0.00994	0.00033bc
*Quercus robur*	0.01627	0.00074ac	0.01889	0.00036de	0.02878	0.00047c	0.01157	0.00020de

**Figure 2:**
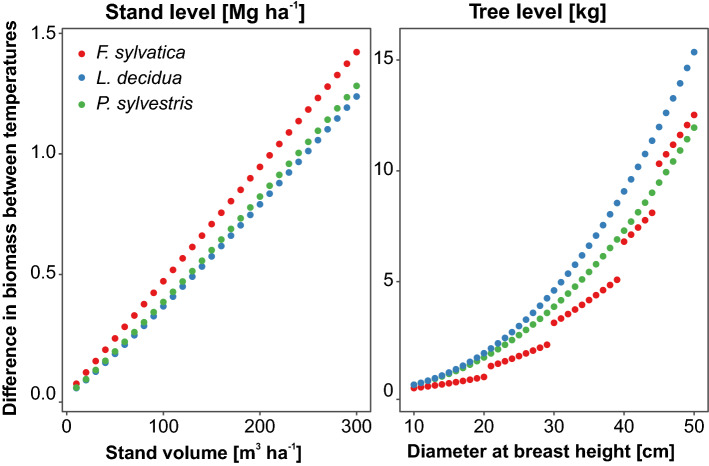
Difference between tree and stand level stem biomass for 75 °C and 105 °C drying temperatures along tree diameter at breast height and stand volume gradients for three example species calculated using species-specific models (Tables S4 and S5). The plot has been generated using R software^38^.

Difference in tree stem biomass in samples of calculated biomass was highest for *L. decidua* (0.34–15.36 kg, with an average of 5.74 ± 0.71 kg), while the lowest—for *F. sylvatica* (0.19–12.25 kg, with an average of 4.05 ± 0.60 kg; Fig. 2). At the stand level we found the highest difference for *F. sylvatica* (0.05–1.42 Mg ha^−1^, with an average of 0.73 ± 0.08 Mg ha^−1^) and the lowest—for *L. decidua* (0.03–1.24 Mg ha^−1^, with an average of 0.61 ± 0.07 Mg ha^−1^; Fig. 2). At the country scale the difference was the highest for *P. sylvestris* (6.3 × 10^6^ Mg), while the lowest—for *Alnus* spp. (0.4 × 10^6^ Mg; Table 4). In total, for species studied the sum of species-specific differences was 11.2 × 10^6^ Mg at the country level (Table 4).

**Table 4:** Country-level stem wood and bark biomass published by Jabłoński and Budniak^39^, biomass recalculated using D coefficients (Table 3) and differences in biomass. All values in 10^6^ Mg.

Species	Biomass published	Biomass recalculated	Difference
*A. alba*	36.6	36.1	0.5
*A. glutinosa* + *A. incana*	50.3	49.9	0.4
*B. pendula* + *B. pubsecens*	77.2	76.5	0.7
*F. sylvatica*	95.8	95.0	0.8
*P. abies*	66	65.2	0.8
*P. sylvestris*	565.7	559.4	6.3
*Q. robur* + *Q. petraea*	142.8	141.1	1.7

## Discussion

Similar to Samuelsson et al.^37^, our study revealed the differences between tree species studied in reference to masses obtained by drying at different temperatures. For foliage, we found that all broadleaved tree species had higher D than coniferous tree species (Table 3). This may be connected with higher leaf area and higher moisture in broadleaved species. The highest D was for leaves of *A. glutinosa*, which is a nitrogen-fixing species and has one of the highest nitrogen concentrations in leaves^40–42^. As lower drying temperature is recommended for analysis of nitrogen content, this difference may be connected with the presence of nitrogen-based compounds. In the case of bark we found the lowest differences between drying temperatures in *B. pendula*, which has the thinnest bark. However, the species with thick bark, such as *Q. robur* and *A. alba* did not differ statistically significantly from *B. pendula*. A similar pattern was found for branch samples. For wood samples we found the lowest D values in three diffuse porous species and the highest in ring-porous *Q. rubra* and coniferous species, especially in *A. alba*. Two species in which wood contains numerous resin ducts—*P. sylvestris* and *L. decidua*—had intermediate D values. This pattern was connected with anatomical build of vessels in diffuse porous wood. In diffuse porous wood vessels in both earlywood and latewood have even lumen diameters, while in ring porous and coniferous wood, latewood has much tighter vessels. These vessels may be opened during drying at higher temperatures.

In the cases of foliage and bark we found impacts of sample mass on differences in masses obtained after drying at two temperatures. In contrast to our expectations, sample mass did not affect wood and branches. This might be connected with the decrease of wood density along stem height^43^, increasing sample sensitivity to difference in drying temperature. Significant influence in the case of foliage was connected with different sample masses for *A. alba* and *P. abies*, connected with length and thickness of their needles. Thus, these differences were likely an effect of species and have no other biological meaning. For bark the trend was connected with higher variability of D in smaller samples. Variance estimated by random factors was low and did not amount to more than 6.2% (in bark). This indicates a low level of site-specificity and good transferability of our results.

Despite the low values of the differences, accounting for about 1%, our study provided values which allowed recalculation of biomasses using models developed from data obtained after different drying temperatures Comparing the differences between drying temperatures with errors of biomass estimation models we can state that the bias connected with drying temperature is lower than biases of tree- and stand-level biomass models. For example, mean difference of tree-level stem biomass was 4.05, 5.74 and 5.67 kg, while RMSE of tree-level models (Table S4) was 8.54, 42.16 and 6.34, for *F. sylvatica*, *L. decidua* and *P. sylvestris*, respectively. For stand level estimations mean difference was 0.73, 0.61 and 0.63 Mg ha^−1^ while models RMSE (Table S4) was 1.30, 0.71 and 6.34, respectively. For that reason we may assume that this source of differences has a low impact on compilation of data sources, such as large biomass databases^44–46^ or generalized allometric equations based on published models^16,47^. Nevertheless, application of D coefficients proposed in our study would surely decrease the uncertainty.

Our study revealed small differences between dry masses of biomass samples dried at the two most commonly used temperatures (i.e. 75 °C and 105 °C). We explored differences between biomass components and tree species studied. The differences among species may result from different morphology of the species studied. We also developed a set of coefficients which may be used to recalculate dry masses between the two temperatures studied. The differences between temperatures are lower than the range of uncertainty of models used in forest biomass assessments. For that reason this source of bias in large-scale forest carbon assessments is low. However, application of our coefficients may decrease these biases.

Although our study covered the eight most economically important tree species in temperate and boreal European forests, the results are not representative for all tree species from this area. Further studies should focus on more tree species rather than on sample size. We believe that D coefficients will be correlated with plant functional traits, especially with wood density and leaf dry matter content. Also, it can be phylogenetically-dependent, similarly to functional traits of wood^48^ and leaves^49,50^. Therefore expansion of data about impacts of drying temperature on a wide set of species and correlation with traits and phylogeny can yield in models providing species-specific D coefficients transferable across understudied taxa. Such models would decrease uncertainty of the global carbon budget connected with uneven drying temperatures in previous studies. Also, this lead to the question whether we should use higher drying temperature, connected with higher carbon footprint, for studies on climate change mitigation capacity of forest ecosystems.

## Methods

### Study material

To assess the differences between drying temperatures we prepared random sets of samples collected during a large biomass assessment in Western Poland; for details based on the example of one species analysis see Jagodziński et al.^14^. We analyzed 1440 (15.6%; Table S1) of 9216 samples of biomass components from eight tree species (Table 1) from 124 study plots (Table S2). These samples consisted of four biomass components (stem bark, stem wood, fine branches, i.e. branches with diameter < 7 cm, and foliage) and eight tree species: *Abies alba* Mill., *Alnus glutinosa* (L.) Gaertn., *Betula pendula* Roth., *Fagus sylvatica* L., *Larix decidua* Mill., *Picea abies* (L.) H. Karst., *Pinus sylvestris* L. and *Quercus robur* L. We assumed that wood and bark of coarse branches (> 7 cm diameter) were similar to stem wood and stem bark, respectively. These species are the most frequent forest-forming trees in Central Europe (Table 1). From each species and each component we randomly selected 30 samples, with the exception of stem wood. For the latter we selected 90 samples, among which 30 were small discs (from the higher parts of stems), 30 intermediate and 30 large discs (from the lower parts of trees), to account for the variability in sample mass. As the research project was focused on aboveground biomass estimation, we did not harvest belowground organs of sample trees. For that reason our study did not cover this important and variable part of tree biomass.

After harvesting, each sample was dried at 75 °C for biomass assessment for other studies^14^ and then stored in our sample warehouse, as study material was harvested from 2015 to 2017. After all samples were collected, we randomly selected our samples and dried them in ovens with forced air circulation (UN 750 and ULE 600, Memmert GmbH + Co. KG, Germany) at 75 °C to constant mass. Then, we weighed the samples with an accuracy of 0.1 g, dried them at 105 °C to constant mass and weighed them again. We determined mass of a few samples representing different dimensions every day and we assumed constant mass when a given sample did not change on two consecutive days.

## Data analysis

All analyses were performed using R software^38^. All mean values are followed by standard error (SE). For each species and component we calculated the relative difference between dry weight of samples dried at both temperatures:

1 $$D=\frac{{m}_{75}-{m}_{105}}{{m}_{105}}$$

where D—difference in masses, m_75_—dry mass of sample at 75 °C and m_105_—dry mass of sample at 105 °C. Calculated D allowed us to recalculate dry mass for each temperatures using the following formulas:

2 $${m}_{105}=\frac{{m}_{75}}{1+D}$$

3 $${m}_{75}=\left(1+D\right)\times {m}_{105}$$

To assess the differences among species studied and impacts on sample mass we used mixed effects analysis of covariance (ANCOVA), assuming sample mass and species as fixed effects and study plot as a random effect. The latter allowed us to evaluate and exclude site-specific effects. We did not account for sample tree as a random effect, as preliminary analyses revealed that this effect on variance was lower than 0.0000001 g. Mixed effects ANCOVA were implemented in the *lmerTest::lmer()* function^54,55^. To assess the differences among species we used Tukey post hoc tests, implemented in the *multcomp::glht()* function. We also used two types of determination coefficients to assess the proportion of variance explained by random and fixed effects. Marginal coefficients of determination (R^2^_m_) express the amount of variance explained only by fixed effects and conditional coefficients of determination (R^2^_c_) express the amount of variance explained by both random and fixed effects. These coefficients were calculated using the *MuMIn::r.squaredGLMM()* function^56^.

We showed impact of drying temperature differences using published biomass models at tree and stand levels and we used published biomass estimation at the country level. The difference also showed the level of uncertainty when drying temperature is unknown (for country level). We extracted tree and plot level biomasses from our previous studies for three species: *P. sylvestris*^57^, *L. decidua*^14^ and *F. sylvatica*^58^ (Table S4). We calculated biomass of tree stem and plot stem biomass across gradients of tree DBH and stand volume, for tree and plot level analyses (Table S5). We provided estimated biomass for DBH ranging from 10 to 50 cm and volume ranging from 10 to 300 m^3^ ha^−1^. For *F. sylvatica* we also used height ranging from 10 to 30 m, as models were based on both DBH and height. For calculations we assumed that bark comprised 15% of stem biomass, similarly as Jabłoński and Budniak^39^ in country-level biomass assessment, as some of models provided only stem biomass.

## Supplementary information

Supplementary Tables.

## Data Availability

All data generated or analyzed during this study are included in this published article (and its Supplementary Information files).
