# Supplementary material for: Consequences of different sample drying temperatures for accuracy of biomass inventories in forest ecosystems
Source: Sci Rep. 2020 Sep 29;10:16009. doi: 10.1038/s41598-020-73053-9 (PMC7525458; doi:10.1038/s41598-020-73053-9)
Supplement: Supplementary file 1 — Supplementary Tables. [file 41598_2020_73053_MOESM1_ESM.docx]

**Consequences of different sample drying temperatures for accuracy of biomass inventories in forest ecosystems**

**Andrzej M. Jagodziński, Marcin K. Dyderski, Kamil Gęsikiewicz, Paweł Horodecki**

**Table S1.** Database with samples used in the study

| **Component** | **Species** | **Plot (Table S2)** | **Sample tree number** | **Dry mass in 75°C [g]** | **Dry mass in 105°C [g]** | **D (difference in masses between temperatures, related to mass in 105°C)** |
| --- | --- | --- | --- | --- | --- | --- |
| bark | *B. pendula* | 30 | 2 | 108.6 | 107.1 | 0.014006 |
| bark | *B. pendula* | 30 | 3 | 76.7 | 75.4 | 0.017241 |
| bark | *B. pendula* | 30 | 4 | 82.7 | 81.7 | 0.012240 |
| bark | *B. pendula* | 45 | 5 | 74.1 | 73.3 | 0.010914 |
| bark | *B. pendula* | 45 | 5 | 114.8 | 113.2 | 0.014134 |
| bark | *B. pendula* | 45 | 6 | 14.3 | 14.3 | 0.000000 |
| bark | *B. pendula* | 58 | 1 | 198.3 | 196.1 | 0.011219 |
| bark | *B. pendula* | 58 | 1 | 281.6 | 277.9 | 0.013314 |
| bark | *B. pendula* | 58 | 2 | 21.9 | 21.6 | 0.013889 |
| bark | *B. pendula* | 58 | 5 | 132.4 | 130.6 | 0.013783 |
| bark | *B. pendula* | 72 | 6 | 30.9 | 30.4 | 0.016447 |
| bark | *B. pendula* | 86 | 2 | 37.3 | 36.6 | 0.019126 |
| bark | *B. pendula* | 86 | 2 | 719.2 | 709.3 | 0.013957 |
| bark | *B. pendula* | 86 | 3 | 43.3 | 42.7 | 0.014052 |
| bark | *B. pendula* | 89 | 2 | 58.5 | 57.7 | 0.013865 |
| bark | *B. pendula* | 89 | 2 | 69.5 | 68.6 | 0.013120 |
| bark | *B. pendula* | 89 | 2 | 36.1 | 35.5 | 0.016901 |
| bark | *B. pendula* | 89 | 2 | 49.3 | 48.8 | 0.010246 |
| bark | *B. pendula* | 89 | 2 | 56.0 | 55.2 | 0.014493 |
| bark | *B. pendula* | 89 | 6 | 19.1 | 18.8 | 0.015957 |
| bark | *B. pendula* | 134 | 4 | 92.2 | 90.7 | 0.016538 |
| bark | *B. pendula* | 134 | 4 | 100.6 | 99.1 | 0.015136 |
| bark | *B. pendula* | 134 | 4 | 105.8 | 104.2 | 0.015355 |
| bark | *B. pendula* | 134 | 4 | 203.3 | 201.0 | 0.011443 |
| bark | *B. pendula* | 134 | 4 | 233.9 | 231.1 | 0.012116 |
| bark | *B. pendula* | 171 | 1 | 22.6 | 22.2 | 0.018018 |
| bark | *B. pendula* | 171 | 1 | 10.3 | 10.3 | 0.000000 |
| bark | *B. pendula* | 171 | 3 | 13.9 | 13.5 | 0.029630 |
| bark | *B. pendula* | 171 | 6 | 9.9 | 9.9 | 0.000000 |
| bark | *B. pendula* | 171 | 8 | 9.2 | 9.1 | 0.010989 |
| bark | *F. sylvatica* | 100 | 6 | 204.4 | 200.9 | 0.017422 |
| bark | *F. sylvatica* | 100 | 6 | 207.3 | 203.6 | 0.018173 |
| bark | *F. sylvatica* | 100 | 6 | 187.5 | 184.1 | 0.018468 |
| bark | *F. sylvatica* | 100 | 6 | 222.9 | 219.1 | 0.017344 |
| bark | *F. sylvatica* | 108 | 2 | 144.1 | 141.9 | 0.015504 |
| bark | *F. sylvatica* | 108 | 5 | 70.7 | 69.4 | 0.018732 |
| bark | *F. sylvatica* | 119 | 2 | 259.4 | 254.5 | 0.019253 |
| bark | *F. sylvatica* | 119 | 5 | 228.4 | 224.8 | 0.016014 |
| bark | *F. sylvatica* | 119 | 5 | 224.3 | 221.3 | 0.013556 |
| bark | *F. sylvatica* | 119 | 7 | 147.9 | 145.7 | 0.015100 |
| bark | *F. sylvatica* | 119 | 8 | 154.1 | 151.3 | 0.018506 |
| bark | *F. sylvatica* | 130 | 4 | 20.4 | 20.0 | 0.020000 |
| bark | *F. sylvatica* | 130 | 4 | 21.2 | 21.1 | 0.004739 |
| bark | *F. sylvatica* | 130 | 4 | 21.5 | 21.3 | 0.009390 |
| bark | *F. sylvatica* | 130 | 4 | 12.2 | 12.2 | 0.000000 |
| bark | *F. sylvatica* | 130 | 4 | 13.3 | 13.0 | 0.023077 |
| bark | *F. sylvatica* | 131 | 1 | 200.9 | 198.1 | 0.014134 |
| bark | *F. sylvatica* | 131 | 4 | 31.5 | 31.5 | 0.000000 |
| bark | *F. sylvatica* | 131 | 5 | 153.6 | 151.3 | 0.015202 |
| bark | *F. sylvatica* | 131 | 8 | 57.4 | 57.0 | 0.007018 |
| bark | *F. sylvatica* | 144 | 2 | 12.9 | 12.7 | 0.015748 |
| bark | *F. sylvatica* | 144 | 3 | 97.5 | 95.7 | 0.018809 |
| bark | *F. sylvatica* | 144 | 3 | 47.9 | 47.2 | 0.014831 |
| bark | *F. sylvatica* | 144 | 4 | 53.1 | 52.5 | 0.011429 |
| bark | *F. sylvatica* | 144 | 6 | 46.4 | 45.5 | 0.019780 |
| bark | *F. sylvatica* | 170 | 6 | 15.5 | 15.4 | 0.006494 |
| bark | *F. sylvatica* | 170 | 6 | 89.3 | 88.0 | 0.014773 |
| bark | *F. sylvatica* | 170 | 6 | 68.1 | 67.2 | 0.013393 |
| bark | *F. sylvatica* | 170 | 7 | 44.8 | 44.1 | 0.015873 |
| bark | *F. sylvatica* | 170 | 7 | 35.3 | 34.7 | 0.017291 |
| bark | *Q. robur* | 21 | 2 | 328.7 | 323.7 | 0.015446 |
| bark | *Q. robur* | 29 | 5 | 425.1 | 417.9 | 0.017229 |
| bark | *Q. robur* | 29 | 5 | 490.8 | 483.1 | 0.015939 |
| bark | *Q. robur* | 48 | 4 | 672.9 | 660.0 | 0.019545 |
| bark | *Q. robur* | 48 | 6 | 561.1 | 552.1 | 0.016301 |
| bark | *Q. robur* | 61 | 6 | 408.6 | 401.1 | 0.018699 |
| bark | *Q. robur* | 61 | 8 | 246.6 | 242.3 | 0.017747 |
| bark | *Q. robur* | 68 | 3 | 69.3 | 68.3 | 0.014641 |
| bark | *Q. robur* | 68 | 3 | 446.4 | 439.6 | 0.015469 |
| bark | *Q. robur* | 127 | 4 | 52.7 | 51.8 | 0.017375 |
| bark | *Q. robur* | 127 | 4 | 82.2 | 80.6 | 0.019851 |
| bark | *Q. robur* | 127 | 4 | 125.0 | 122.9 | 0.017087 |
| bark | *Q. robur* | 127 | 4 | 32.1 | 31.6 | 0.015823 |
| bark | *Q. robur* | 127 | 4 | 50.7 | 49.8 | 0.018072 |
| bark | *Q. robur* | 133 | 2 | 17.7 | 17.5 | 0.011429 |
| bark | *Q. robur* | 133 | 2 | 27.3 | 26.8 | 0.018657 |
| bark | *Q. robur* | 133 | 2 | 34.0 | 33.4 | 0.017964 |
| bark | *Q. robur* | 137 | 4 | 110.8 | 109.2 | 0.014652 |
| bark | *Q. robur* | 137 | 4 | 127.2 | 125.1 | 0.016787 |
| bark | *Q. robur* | 137 | 4 | 145.3 | 143.3 | 0.013957 |
| bark | *Q. robur* | 137 | 4 | 1000.6 | 981.4 | 0.019564 |
| bark | *Q. robur* | 137 | 4 | 821.6 | 808.3 | 0.016454 |
| bark | *Q. robur* | 141 | 1 | 15.3 | 15.0 | 0.020000 |
| bark | *Q. robur* | 141 | 3 | 12.5 | 12.4 | 0.008065 |
| bark | *Q. robur* | 141 | 5 | 11.1 | 11.1 | 0.000000 |
| bark | *Q. robur* | 143 | 3 | 143.4 | 141.2 | 0.015581 |
| bark | *Q. robur* | 143 | 7 | 59.1 | 57.9 | 0.020725 |
| bark | *Q. robur* | 143 | 7 | 370.4 | 363.9 | 0.017862 |
| bark | *Q. robur* | 143 | 8 | 429.7 | 421.1 | 0.020423 |
| bark | *Q. robur* | 143 | 8 | 321.0 | 315.7 | 0.016788 |
| bark | *A. alba* | 105 | 1 | 76.7 | 75.3 | 0.018592 |
| bark | *A. alba* | 105 | 5 | 215.6 | 211.6 | 0.018904 |
| bark | *A. alba* | 105 | 7 | 82.2 | 81.0 | 0.014815 |
| bark | *A. alba* | 105 | 8 | 100.3 | 98.8 | 0.015182 |
| bark | *A. alba* | 105 | 8 | 82.1 | 80.7 | 0.017348 |
| bark | *A. alba* | 121 | 1 | 147.1 | 144.3 | 0.019404 |
| bark | *A. alba* | 121 | 2 | 24.2 | 23.9 | 0.012552 |
| bark | *A. alba* | 121 | 2 | 162.8 | 160.5 | 0.014330 |
| bark | *A. alba* | 121 | 5 | 72.2 | 70.9 | 0.018336 |
| bark | *A. alba* | 121 | 6 | 65.1 | 64.0 | 0.017187 |
| bark | *A. alba* | 122 | 4 | 440.1 | 432.0 | 0.018750 |
| bark | *A. alba* | 122 | 4 | 381.7 | 375.5 | 0.016511 |
| bark | *A. alba* | 122 | 4 | 292.2 | 287.2 | 0.017409 |
| bark | *A. alba* | 122 | 4 | 177.5 | 174.1 | 0.019529 |
| bark | *A. alba* | 122 | 4 | 72.6 | 71.3 | 0.018233 |
| bark | *A. alba* | 124 | 2 | 407.5 | 401.9 | 0.013934 |
| bark | *A. alba* | 124 | 3 | 715.9 | 703.9 | 0.017048 |
| bark | *A. alba* | 124 | 3 | 461.3 | 453.1 | 0.018098 |
| bark | *A. alba* | 124 | 4 | 181.1 | 178.0 | 0.017416 |
| bark | *A. alba* | 124 | 4 | 376.3 | 369.7 | 0.017852 |
| bark | *A. alba* | 124 | 4 | 423.0 | 415.8 | 0.017316 |
| bark | *A. alba* | 124 | 4 | 503.5 | 495.0 | 0.017172 |
| bark | *A. alba* | 124 | 4 | 539.8 | 532.7 | 0.013328 |
| bark | *A. alba* | 124 | 6 | 354.2 | 348.8 | 0.015482 |
| bark | *A. alba* | 124 | 8 | 239.0 | 234.5 | 0.019190 |
| bark | *A. alba* | 125 | 1 | 440.1 | 432.1 | 0.018514 |
| bark | *A. alba* | 125 | 1 | 192.8 | 189.2 | 0.019027 |
| bark | *A. alba* | 125 | 4 | 131.8 | 129.5 | 0.017761 |
| bark | *A. alba* | 125 | 5 | 133.7 | 131.6 | 0.015957 |
| bark | *A. alba* | 125 | 7 | 78.2 | 76.6 | 0.020888 |
| bark | *L. decidua* | 25 | 1 | 361.0 | 353.4 | 0.021505 |
| bark | *L. decidua* | 25 | 2 | 281.5 | 276.5 | 0.018083 |
| bark | *L. decidua* | 25 | 4 | 317.0 | 310.5 | 0.020934 |
| bark | *L. decidua* | 54 | 1 | 506.6 | 498.1 | 0.017065 |
| bark | *L. decidua* | 54 | 5 | 363.4 | 356.0 | 0.020787 |
| bark | *L. decidua* | 59 | 3 | 374.0 | 366.8 | 0.019629 |
| bark | *L. decidua* | 59 | 4 | 333.6 | 327.3 | 0.019248 |
| bark | *L. decidua* | 63 | 2 | 280.4 | 274.5 | 0.021494 |
| bark | *L. decidua* | 63 | 3 | 214.2 | 210.2 | 0.019029 |
| bark | *L. decidua* | 63 | 4 | 360.6 | 353.4 | 0.020374 |
| bark | *L. decidua* | 85 | 1 | 348.0 | 341.0 | 0.020528 |
| bark | *L. decidua* | 85 | 7 | 171.5 | 168.7 | 0.016598 |
| bark | *L. decidua* | 87 | 4 | 75.1 | 73.6 | 0.020380 |
| bark | *L. decidua* | 87 | 4 | 76.5 | 75.1 | 0.018642 |
| bark | *L. decidua* | 87 | 4 | 61.0 | 60.1 | 0.014975 |
| bark | *L. decidua* | 87 | 4 | 71.0 | 69.8 | 0.017192 |
| bark | *L. decidua* | 87 | 4 | 76.5 | 75.2 | 0.017287 |
| bark | *L. decidua* | 99 | 4 | 638.8 | 627.1 | 0.018657 |
| bark | *L. decidua* | 99 | 4 | 183.0 | 179.6 | 0.018931 |
| bark | *L. decidua* | 99 | 4 | 220.1 | 216.2 | 0.018039 |
| bark | *L. decidua* | 99 | 4 | 230.8 | 226.8 | 0.017637 |
| bark | *L. decidua* | 99 | 4 | 277.9 | 272.9 | 0.018322 |
| bark | *L. decidua* | 120 | 2 | 137.0 | 134.6 | 0.017831 |
| bark | *L. decidua* | 120 | 2 | 553.1 | 543.7 | 0.017289 |
| bark | *L. decidua* | 120 | 3 | 92.7 | 91.2 | 0.016447 |
| bark | *L. decidua* | 120 | 3 | 563.6 | 553.6 | 0.018064 |
| bark | *L. decidua* | 120 | 3 | 245.7 | 241.6 | 0.016970 |
| bark | *L. decidua* | 139 | 2 | 9.6 | 9.4 | 0.021277 |
| bark | *L. decidua* | 139 | 3 | 10.3 | 10.1 | 0.019802 |
| bark | *L. decidua* | 139 | 4 | 9.0 | 8.9 | 0.011236 |
| bark | *A. glutinosa* | 19 | 2 | 302.1 | 296.5 | 0.018887 |
| bark | *A. glutinosa* | 19 | 6 | 37.4 | 36.9 | 0.013550 |
| bark | *A. glutinosa* | 19 | 7 | 152.2 | 149.3 | 0.019424 |
| bark | *A. glutinosa* | 19 | 8 | 24.6 | 24.3 | 0.012346 |
| bark | *A. glutinosa* | 20 | 1 | 208.9 | 205.6 | 0.016051 |
| bark | *A. glutinosa* | 20 | 1 | 257.8 | 253.6 | 0.016562 |
| bark | *A. glutinosa* | 20 | 2 | 176.6 | 173.5 | 0.017867 |
| bark | *A. glutinosa* | 20 | 4 | 37.4 | 36.9 | 0.013550 |
| bark | *A. glutinosa* | 20 | 4 | 145.0 | 142.5 | 0.017544 |
| bark | *A. glutinosa* | 20 | 8 | 53.6 | 52.6 | 0.019011 |
| bark | *A. glutinosa* | 53 | 1 | 167.6 | 164.8 | 0.016990 |
| bark | *A. glutinosa* | 53 | 2 | 32.7 | 32.2 | 0.015528 |
| bark | *A. glutinosa* | 66 | 1 | 94.5 | 93.0 | 0.016129 |
| bark | *A. glutinosa* | 66 | 2 | 77.2 | 75.7 | 0.019815 |
| bark | *A. glutinosa* | 102 | 3 | 170.4 | 167.0 | 0.020359 |
| bark | *A. glutinosa* | 102 | 4 | 23.0 | 22.9 | 0.004367 |
| bark | *A. glutinosa* | 102 | 4 | 62.6 | 61.5 | 0.017886 |
| bark | *A. glutinosa* | 102 | 4 | 177.3 | 174.5 | 0.016046 |
| bark | *A. glutinosa* | 102 | 4 | 141.2 | 138.6 | 0.018759 |
| bark | *A. glutinosa* | 102 | 4 | 154.0 | 151.3 | 0.017845 |
| bark | *A. glutinosa* | 102 | 4 | 162.5 | 160.0 | 0.015625 |
| bark | *A. glutinosa* | 102 | 4 | 168.0 | 165.0 | 0.018182 |
| bark | *A. glutinosa* | 102 | 4 | 161.7 | 159.8 | 0.011890 |
| bark | *A. glutinosa* | 102 | 6 | 55.3 | 54.4 | 0.016544 |
| bark | *A. glutinosa* | 102 | 8 | 103.8 | 101.1 | 0.026706 |
| bark | *A. glutinosa* | 135 | 1 | 36.6 | 36.2 | 0.011050 |
| bark | *A. glutinosa* | 135 | 1 | 24.5 | 24.1 | 0.016598 |
| bark | *A. glutinosa* | 135 | 3 | 20.8 | 20.5 | 0.014634 |
| bark | *A. glutinosa* | 135 | 3 | 19.9 | 19.7 | 0.010152 |
| bark | *A. glutinosa* | 135 | 5 | 13.6 | 13.4 | 0.014925 |
| bark | *P. sylvestris* | 22 | 7 | 44.0 | 43.3 | 0.016166 |
| bark | *P. sylvestris* | 23 | 3 | 58.3 | 57.1 | 0.021016 |
| bark | *P. sylvestris* | 26 | 1 | 362.8 | 354.9 | 0.022260 |
| bark | *P. sylvestris* | 33 | 6 | 16.5 | 16.2 | 0.018519 |
| bark | *P. sylvestris* | 52 | 1 | 102.5 | 100.6 | 0.018887 |
| bark | *P. sylvestris* | 52 | 1 | 189.9 | 186.4 | 0.018777 |
| bark | *P. sylvestris* | 52 | 1 | 367.7 | 360.1 | 0.021105 |
| bark | *P. sylvestris* | 52 | 5 | 196.0 | 192.6 | 0.017653 |
| bark | *P. sylvestris* | 88 | 2 | 20.2 | 19.9 | 0.015075 |
| bark | *P. sylvestris* | 88 | 4 | 123.5 | 121.1 | 0.019818 |
| bark | *P. sylvestris* | 88 | 4 | 86.4 | 84.9 | 0.017668 |
| bark | *P. sylvestris* | 88 | 4 | 67.4 | 66.3 | 0.016591 |
| bark | *P. sylvestris* | 101 | 1 | 325.9 | 320.4 | 0.017166 |
| bark | *P. sylvestris* | 101 | 2 | 38.2 | 37.6 | 0.015957 |
| bark | *P. sylvestris* | 101 | 2 | 400.9 | 392.4 | 0.021662 |
| bark | *P. sylvestris* | 101 | 3 | 115.6 | 113.6 | 0.017606 |
| bark | *P. sylvestris* | 101 | 4 | 31.0 | 30.7 | 0.009772 |
| bark | *P. sylvestris* | 101 | 4 | 48.0 | 47.2 | 0.016949 |
| bark | *P. sylvestris* | 101 | 4 | 567.0 | 555.1 | 0.021438 |
| bark | *P. sylvestris* | 101 | 4 | 65.7 | 64.6 | 0.017028 |
| bark | *P. sylvestris* | 101 | 4 | 71.7 | 70.4 | 0.018466 |
| bark | *P. sylvestris* | 101 | 5 | 189.4 | 185.5 | 0.021024 |
| bark | *P. sylvestris* | 128 | 2 | 71.0 | 69.6 | 0.020115 |
| bark | *P. sylvestris* | 128 | 2 | 115.6 | 113.2 | 0.021201 |
| bark | *P. sylvestris* | 128 | 2 | 25.0 | 24.2 | 0.033058 |
| bark | *P. sylvestris* | 128 | 2 | 26.4 | 26.1 | 0.011494 |
| bark | *P. sylvestris* | 138 | 1 | 57.8 | 56.8 | 0.017606 |
| bark | *P. sylvestris* | 138 | 1 | 109.0 | 106.7 | 0.021556 |
| bark | *P. sylvestris* | 138 | 1 | 36.1 | 35.3 | 0.022663 |
| bark | *P. sylvestris* | 138 | 1 | 38.5 | 37.8 | 0.018519 |
| bark | *P. abies* | 28 | 6 | 34.2 | 33.7 | 0.014837 |
| bark | *P. abies* | 38 | 5 | 123.5 | 121.0 | 0.020661 |
| bark | *P. abies* | 82 | 1 | 265.5 | 260.4 | 0.019585 |
| bark | *P. abies* | 97 | 6 | 17.3 | 17.0 | 0.017647 |
| bark | *P. abies* | 97 | 6 | 48.5 | 47.6 | 0.018908 |
| bark | *P. abies* | 98 | 5 | 119.5 | 116.6 | 0.024871 |
| bark | *P. abies* | 103 | 2 | 19.3 | 19.1 | 0.010471 |
| bark | *P. abies* | 103 | 3 | 22.3 | 21.9 | 0.018265 |
| bark | *P. abies* | 103 | 5 | 15.2 | 15.0 | 0.013333 |
| bark | *P. abies* | 114 | 4 | 81.6 | 80.3 | 0.016189 |
| bark | *P. abies* | 114 | 4 | 440.4 | 431.4 | 0.020862 |
| bark | *P. abies* | 114 | 4 | 384.0 | 376.3 | 0.020462 |
| bark | *P. abies* | 117 | 5 | 106.0 | 103.8 | 0.021195 |
| bark | *P. abies* | 117 | 7 | 94.5 | 92.3 | 0.023835 |
| bark | *P. abies* | 118 | 1 | 40.2 | 39.3 | 0.022901 |
| bark | *P. abies* | 118 | 2 | 120.3 | 117.9 | 0.020356 |
| bark | *P. abies* | 118 | 3 | 172.9 | 169.4 | 0.020661 |
| bark | *P. abies* | 118 | 4 | 97.4 | 95.3 | 0.022036 |
| bark | *P. abies* | 123 | 1 | 44.3 | 43.5 | 0.018391 |
| bark | *P. abies* | 123 | 4 | 20.3 | 20.1 | 0.009950 |
| bark | *P. abies* | 123 | 4 | 18.9 | 18.6 | 0.016129 |
| bark | *P. abies* | 123 | 8 | 13.1 | 12.9 | 0.015504 |
| bark | *P. abies* | 123 | 8 | 11.8 | 11.8 | 0.000000 |
| bark | *P. abies* | 126 | 4 | 151.9 | 148.4 | 0.023585 |
| bark | *P. abies* | 126 | 4 | 98.0 | 96.1 | 0.019771 |
| bark | *P. abies* | 126 | 4 | 111.6 | 109.0 | 0.023853 |
| bark | *P. abies* | 169 | 5 | 58.4 | 57.1 | 0.022767 |
| bark | *P. abies* | 169 | 5 | 184.1 | 180.0 | 0.022778 |
| bark | *P. abies* | 169 | 6 | 207.1 | 202.9 | 0.020700 |
| bark | *P. abies* | 169 | 6 | 128.9 | 125.8 | 0.024642 |
| branches | *B. pendula* | 45 | 2 | 192.9 | 190.5 | 0.012598 |
| branches | *B. pendula* | 45 | 3 | 181.8 | 179.2 | 0.014509 |
| branches | *B. pendula* | 45 | 5 | 213.6 | 210.7 | 0.013764 |
| branches | *B. pendula* | 45 | 7 | 233.3 | 229.7 | 0.015673 |
| branches | *B. pendula* | 58 | 2 | 170.4 | 168.0 | 0.014286 |
| branches | *B. pendula* | 58 | 5 | 177.2 | 174.7 | 0.014310 |
| branches | *B. pendula* | 86 | 4 | 170.7 | 168.0 | 0.016071 |
| branches | *B. pendula* | 86 | 7 | 248.4 | 244.7 | 0.015121 |
| branches | *B. pendula* | 89 | 1 | 165.1 | 162.9 | 0.013505 |
| branches | *B. pendula* | 89 | 2 | 160.6 | 158.6 | 0.012610 |
| branches | *B. pendula* | 89 | 3 | 169.4 | 166.9 | 0.014979 |
| branches | *B. pendula* | 89 | 4 | 163.8 | 161.5 | 0.014241 |
| branches | *B. pendula* | 89 | 7 | 172.4 | 169.8 | 0.015312 |
| branches | *B. pendula* | 89 | 8 | 146.2 | 144.0 | 0.015278 |
| branches | *B. pendula* | 134 | 1 | 197.5 | 194.7 | 0.014381 |
| branches | *B. pendula* | 134 | 2 | 161.2 | 159.0 | 0.013836 |
| branches | *B. pendula* | 134 | 3 | 174.8 | 172.3 | 0.014510 |
| branches | *B. pendula* | 134 | 4 | 198.8 | 196.0 | 0.014286 |
| branches | *B. pendula* | 134 | 5 | 169.1 | 166.6 | 0.015006 |
| branches | *B. pendula* | 134 | 6 | 197.4 | 194.6 | 0.014388 |
| branches | *B. pendula* | 134 | 7 | 156.1 | 153.6 | 0.016276 |
| branches | *B. pendula* | 134 | 8 | 160.6 | 158.4 | 0.013889 |
| branches | *B. pendula* | 171 | 1 | 184.0 | 181.3 | 0.014892 |
| branches | *B. pendula* | 171 | 2 | 193.0 | 190.2 | 0.014721 |
| branches | *B. pendula* | 171 | 3 | 173.2 | 170.5 | 0.015836 |
| branches | *B. pendula* | 171 | 4 | 171.3 | 168.6 | 0.016014 |
| branches | *B. pendula* | 171 | 5 | 168.4 | 165.8 | 0.015682 |
| branches | *B. pendula* | 171 | 6 | 174.9 | 172.1 | 0.016270 |
| branches | *B. pendula* | 171 | 7 | 186.4 | 183.2 | 0.017467 |
| branches | *B. pendula* | 171 | 8 | 111.7 | 109.7 | 0.018232 |
| branches | *F. sylvatica* | 100 | 2 | 222.7 | 218.9 | 0.017360 |
| branches | *F. sylvatica* | 100 | 4 | 238.1 | 234.8 | 0.013927 |
| branches | *F. sylvatica* | 100 | 6 | 229.2 | 225.7 | 0.015507 |
| branches | *F. sylvatica* | 100 | 8 | 239.1 | 235.0 | 0.017447 |
| branches | *F. sylvatica* | 116 | 3 | 184.1 | 181.2 | 0.016004 |
| branches | *F. sylvatica* | 116 | 4 | 201.4 | 197.8 | 0.018200 |
| branches | *F. sylvatica* | 116 | 6 | 173.1 | 170.3 | 0.016442 |
| branches | *F. sylvatica* | 116 | 7 | 215.5 | 212.0 | 0.016509 |
| branches | *F. sylvatica* | 116 | 8 | 199.8 | 196.7 | 0.015760 |
| branches | *F. sylvatica* | 119 | 2 | 191.5 | 188.5 | 0.015915 |
| branches | *F. sylvatica* | 119 | 3 | 236.1 | 232.4 | 0.015921 |
| branches | *F. sylvatica* | 119 | 5 | 187.8 | 184.7 | 0.016784 |
| branches | *F. sylvatica* | 119 | 6 | 174.6 | 171.6 | 0.017483 |
| branches | *F. sylvatica* | 119 | 8 | 273.6 | 269.3 | 0.015967 |
| branches | *F. sylvatica* | 132 | 1 | 210.7 | 207.2 | 0.016892 |
| branches | *F. sylvatica* | 132 | 4 | 251.5 | 248.0 | 0.014113 |
| branches | *F. sylvatica* | 132 | 5 | 174.9 | 172.1 | 0.016270 |
| branches | *F. sylvatica* | 132 | 6 | 214.0 | 210.1 | 0.018563 |
| branches | *F. sylvatica* | 132 | 7 | 194.3 | 191.5 | 0.014621 |
| branches | *F. sylvatica* | 144 | 2 | 181.2 | 178.5 | 0.015126 |
| branches | *F. sylvatica* | 144 | 3 | 190.0 | 186.9 | 0.016586 |
| branches | *F. sylvatica* | 144 | 8 | 162.2 | 159.7 | 0.015654 |
| branches | *F. sylvatica* | 170 | 1 | 167.8 | 165.4 | 0.014510 |
| branches | *F. sylvatica* | 170 | 2 | 180.7 | 177.8 | 0.016310 |
| branches | *F. sylvatica* | 170 | 3 | 176.1 | 173.7 | 0.013817 |
| branches | *F. sylvatica* | 170 | 4 | 173.8 | 171.1 | 0.015780 |
| branches | *F. sylvatica* | 170 | 5 | 171.6 | 169.1 | 0.014784 |
| branches | *F. sylvatica* | 170 | 6 | 181.3 | 178.4 | 0.016256 |
| branches | *F. sylvatica* | 170 | 7 | 192.1 | 189.3 | 0.014791 |
| branches | *F. sylvatica* | 170 | 8 | 171.0 | 168.3 | 0.016043 |
| branches | *Q. robur* | 127 | 1 | 222.0 | 217.9 | 0.018816 |
| branches | *Q. robur* | 127 | 2 | 222.5 | 218.3 | 0.019240 |
| branches | *Q. robur* | 127 | 3 | 181.6 | 178.2 | 0.019080 |
| branches | *Q. robur* | 127 | 4 | 214.6 | 210.5 | 0.019477 |
| branches | *Q. robur* | 127 | 5 | 198.2 | 194.3 | 0.020072 |
| branches | *Q. robur* | 127 | 6 | 191.7 | 188.1 | 0.019139 |
| branches | *Q. robur* | 127 | 7 | 212.0 | 208.1 | 0.018741 |
| branches | *Q. robur* | 133 | 1 | 171.1 | 168.4 | 0.016033 |
| branches | *Q. robur* | 133 | 2 | 155.6 | 152.7 | 0.018991 |
| branches | *Q. robur* | 133 | 3 | 152.4 | 149.8 | 0.017356 |
| branches | *Q. robur* | 133 | 4 | 171.6 | 168.5 | 0.018398 |
| branches | *Q. robur* | 133 | 5 | 155.3 | 152.6 | 0.017693 |
| branches | *Q. robur* | 133 | 6 | 159.8 | 156.5 | 0.021086 |
| branches | *Q. robur* | 133 | 7 | 120.3 | 119.0 | 0.010924 |
| branches | *Q. robur* | 133 | 8 | 58.8 | 57.6 | 0.020833 |
| branches | *Q. robur* | 137 | 1 | 185.5 | 181.7 | 0.020914 |
| branches | *Q. robur* | 137 | 2 | 191.1 | 187.5 | 0.019200 |
| branches | *Q. robur* | 137 | 3 | 210.0 | 206.0 | 0.019417 |
| branches | *Q. robur* | 137 | 4 | 165.9 | 163.0 | 0.017791 |
| branches | *Q. robur* | 137 | 5 | 174.4 | 170.7 | 0.021675 |
| branches | *Q. robur* | 137 | 7 | 173.6 | 170.6 | 0.017585 |
| branches | *Q. robur* | 137 | 8 | 173.8 | 170.8 | 0.017564 |
| branches | *Q. robur* | 141 | 1 | 194.1 | 190.4 | 0.019433 |
| branches | *Q. robur* | 141 | 3 | 178.1 | 174.8 | 0.018879 |
| branches | *Q. robur* | 143 | 1 | 181.5 | 177.9 | 0.020236 |
| branches | *Q. robur* | 143 | 3 | 175.9 | 172.4 | 0.020302 |
| branches | *Q. robur* | 143 | 4 | 178.2 | 174.7 | 0.020034 |
| branches | *Q. robur* | 143 | 5 | 186.7 | 183.4 | 0.017993 |
| branches | *Q. robur* | 143 | 7 | 176.3 | 172.7 | 0.020845 |
| branches | *Q. robur* | 143 | 8 | 189.4 | 185.9 | 0.018827 |
| branches | *A. alba* | 109 | 2 | 202.7 | 199.3 | 0.017060 |
| branches | *A. alba* | 109 | 4 | 193.2 | 189.9 | 0.017378 |
| branches | *A. alba* | 109 | 7 | 182.6 | 179.8 | 0.015573 |
| branches | *A. alba* | 112 | 5 | 175.0 | 172.3 | 0.015670 |
| branches | *A. alba* | 112 | 6 | 209.0 | 205.2 | 0.018519 |
| branches | *A. alba* | 113 | 1 | 135.1 | 132.8 | 0.017319 |
| branches | *A. alba* | 113 | 4 | 179.9 | 176.7 | 0.018110 |
| branches | *A. alba* | 113 | 8 | 167.4 | 164.5 | 0.017629 |
| branches | *A. alba* | 121 | 1 | 155.6 | 153.1 | 0.016329 |
| branches | *A. alba* | 121 | 2 | 170.3 | 167.7 | 0.015504 |
| branches | *A. alba* | 121 | 3 | 208.0 | 204.6 | 0.016618 |
| branches | *A. alba* | 121 | 4 | 185.7 | 182.6 | 0.016977 |
| branches | *A. alba* | 121 | 5 | 118.2 | 116.1 | 0.018088 |
| branches | *A. alba* | 121 | 6 | 160.2 | 157.5 | 0.017143 |
| branches | *A. alba* | 121 | 7 | 172.3 | 169.3 | 0.017720 |
| branches | *A. alba* | 121 | 8 | 167.4 | 164.3 | 0.018868 |
| branches | *A. alba* | 122 | 2 | 169.9 | 167.4 | 0.014934 |
| branches | *A. alba* | 122 | 3 | 190.5 | 187.3 | 0.017085 |
| branches | *A. alba* | 122 | 4 | 190.2 | 187.2 | 0.016026 |
| branches | *A. alba* | 122 | 6 | 203.1 | 199.7 | 0.017026 |
| branches | *A. alba* | 122 | 7 | 188.7 | 185.4 | 0.017799 |
| branches | *A. alba* | 122 | 8 | 157.9 | 155.3 | 0.016742 |
| branches | *A. alba* | 124 | 1 | 186.1 | 183.2 | 0.015830 |
| branches | *A. alba* | 124 | 2 | 163.2 | 160.4 | 0.017456 |
| branches | *A. alba* | 124 | 3 | 158.1 | 155.4 | 0.017375 |
| branches | *A. alba* | 124 | 4 | 166.3 | 163.5 | 0.017125 |
| branches | *A. alba* | 124 | 5 | 174.8 | 171.8 | 0.017462 |
| branches | *A. alba* | 124 | 6 | 154.3 | 152.0 | 0.015132 |
| branches | *A. alba* | 124 | 7 | 174.2 | 171.0 | 0.018713 |
| branches | *A. alba* | 124 | 8 | 194.8 | 191.3 | 0.018296 |
| branches | *L. decidua* | 25 | 6 | 235.6 | 230.7 | 0.021240 |
| branches | *L. decidua* | 36 | 6 | 174.5 | 171.0 | 0.020468 |
| branches | *L. decidua* | 44 | 3 | 269.5 | 263.3 | 0.023547 |
| branches | *L. decidua* | 44 | 8 | 188.1 | 184.3 | 0.020619 |
| branches | *L. decidua* | 54 | 7 | 305.5 | 299.0 | 0.021739 |
| branches | *L. decidua* | 54 | 8 | 262.3 | 257.1 | 0.020226 |
| branches | *L. decidua* | 63 | 3 | 213.2 | 208.8 | 0.021073 |
| branches | *L. decidua* | 63 | 4 | 195.4 | 191.8 | 0.018770 |
| branches | *L. decidua* | 63 | 5 | 185.6 | 181.9 | 0.020341 |
| branches | *L. decidua* | 63 | 6 | 215.4 | 211.0 | 0.020853 |
| branches | *L. decidua* | 75 | 3 | 221.9 | 217.8 | 0.018825 |
| branches | *L. decidua* | 75 | 6 | 157.6 | 154.4 | 0.020725 |
| branches | *L. decidua* | 85 | 6 | 217.7 | 213.2 | 0.021107 |
| branches | *L. decidua* | 87 | 1 | 157.1 | 154.1 | 0.019468 |
| branches | *L. decidua* | 87 | 2 | 156.5 | 153.8 | 0.017555 |
| branches | *L. decidua* | 87 | 3 | 173.0 | 169.6 | 0.020047 |
| branches | *L. decidua* | 87 | 4 | 144.1 | 141.3 | 0.019816 |
| branches | *L. decidua* | 87 | 5 | 156.7 | 153.9 | 0.018194 |
| branches | *L. decidua* | 87 | 6 | 152.0 | 149.2 | 0.018767 |
| branches | *L. decidua* | 87 | 7 | 170.2 | 166.7 | 0.020996 |
| branches | *L. decidua* | 87 | 8 | 153.6 | 150.6 | 0.019920 |
| branches | *L. decidua* | 99 | 1 | 206.4 | 202.4 | 0.019763 |
| branches | *L. decidua* | 99 | 2 | 207.4 | 203.3 | 0.020167 |
| branches | *L. decidua* | 99 | 3 | 201.0 | 197.0 | 0.020305 |
| branches | *L. decidua* | 99 | 4 | 225.7 | 221.2 | 0.020344 |
| branches | *L. decidua* | 99 | 5 | 200.3 | 196.5 | 0.019338 |
| branches | *L. decidua* | 99 | 6 | 194.2 | 190.5 | 0.019423 |
| branches | *L. decidua* | 99 | 7 | 209.3 | 205.2 | 0.019981 |
| branches | *L. decidua* | 120 | 3 | 207.8 | 204.1 | 0.018128 |
| branches | *L. decidua* | 120 | 6 | 171.2 | 167.8 | 0.020262 |
| branches | *A. glutinosa* | 19 | 2 | 128.6 | 126.7 | 0.014996 |
| branches | *A. glutinosa* | 19 | 4 | 173.1 | 171.0 | 0.012281 |
| branches | *A. glutinosa* | 20 | 2 | 187.9 | 185.2 | 0.014579 |
| branches | *A. glutinosa* | 20 | 5 | 224.6 | 221.1 | 0.015830 |
| branches | *A. glutinosa* | 20 | 6 | 172.8 | 170.2 | 0.015276 |
| branches | *A. glutinosa* | 20 | 7 | 157.4 | 154.7 | 0.017453 |
| branches | *A. glutinosa* | 31 | 3 | 231.1 | 227.4 | 0.016271 |
| branches | *A. glutinosa* | 50 | 4 | 169.2 | 166.3 | 0.017438 |
| branches | *A. glutinosa* | 53 | 3 | 174.1 | 171.6 | 0.014569 |
| branches | *A. glutinosa* | 53 | 6 | 148.7 | 146.5 | 0.015017 |
| branches | *A. glutinosa* | 53 | 7 | 149.2 | 146.2 | 0.020520 |
| branches | *A. glutinosa* | 55 | 3 | 211.3 | 207.9 | 0.016354 |
| branches | *A. glutinosa* | 55 | 4 | 233.3 | 229.7 | 0.015673 |
| branches | *A. glutinosa* | 55 | 5 | 241.7 | 237.6 | 0.017256 |
| branches | *A. glutinosa* | 102 | 1 | 173.1 | 170.3 | 0.016442 |
| branches | *A. glutinosa* | 102 | 2 | 176.5 | 173.8 | 0.015535 |
| branches | *A. glutinosa* | 102 | 3 | 186.5 | 183.6 | 0.015795 |
| branches | *A. glutinosa* | 102 | 4 | 172.0 | 169.0 | 0.017751 |
| branches | *A. glutinosa* | 102 | 5 | 191.2 | 188.3 | 0.015401 |
| branches | *A. glutinosa* | 102 | 6 | 186.9 | 183.8 | 0.016866 |
| branches | *A. glutinosa* | 102 | 7 | 191.3 | 188.0 | 0.017553 |
| branches | *A. glutinosa* | 102 | 8 | 188.3 | 185.1 | 0.017288 |
| branches | *A. glutinosa* | 135 | 1 | 183.2 | 180.5 | 0.014958 |
| branches | *A. glutinosa* | 135 | 2 | 151.8 | 149.3 | 0.016745 |
| branches | *A. glutinosa* | 135 | 3 | 174.4 | 171.5 | 0.016910 |
| branches | *A. glutinosa* | 135 | 4 | 183.7 | 180.9 | 0.015478 |
| branches | *A. glutinosa* | 135 | 5 | 174.2 | 171.3 | 0.016929 |
| branches | *A. glutinosa* | 135 | 6 | 171.2 | 168.5 | 0.016024 |
| branches | *A. glutinosa* | 135 | 7 | 146.1 | 143.8 | 0.015994 |
| branches | *A. glutinosa* | 135 | 8 | 109.5 | 107.6 | 0.017658 |
| branches | *P. sylvestris* | 22 | 2 | 198.2 | 195.1 | 0.015889 |
| branches | *P. sylvestris* | 23 | 2 | 204.4 | 201.0 | 0.016915 |
| branches | *P. sylvestris* | 23 | 7 | 199.2 | 195.8 | 0.017365 |
| branches | *P. sylvestris* | 26 | 8 | 155.2 | 152.3 | 0.019041 |
| branches | *P. sylvestris* | 33 | 4 | 154.4 | 151.5 | 0.019142 |
| branches | *P. sylvestris* | 52 | 1 | 150.0 | 147.4 | 0.017639 |
| branches | *P. sylvestris* | 56 | 2 | 209.9 | 206.5 | 0.016465 |
| branches | *P. sylvestris* | 56 | 7 | 202.0 | 198.5 | 0.017632 |
| branches | *P. sylvestris* | 62 | 1 | 167.3 | 164.7 | 0.015786 |
| branches | *P. sylvestris* | 88 | 1 | 95.0 | 93.4 | 0.017131 |
| branches | *P. sylvestris* | 88 | 2 | 138.6 | 136.3 | 0.016875 |
| branches | *P. sylvestris* | 88 | 4 | 121.5 | 119.6 | 0.015886 |
| branches | *P. sylvestris* | 88 | 5 | 135.6 | 133.2 | 0.018018 |
| branches | *P. sylvestris* | 88 | 6 | 150.3 | 147.5 | 0.018983 |
| branches | *P. sylvestris* | 88 | 7 | 142.9 | 140.1 | 0.019986 |
| branches | *P. sylvestris* | 88 | 8 | 127.4 | 125.2 | 0.017572 |
| branches | *P. sylvestris* | 113 | 1 | 133.7 | 131.5 | 0.016730 |
| branches | *P. sylvestris* | 128 | 1 | 139.8 | 137.4 | 0.017467 |
| branches | *P. sylvestris* | 128 | 2 | 125.0 | 122.7 | 0.018745 |
| branches | *P. sylvestris* | 128 | 3 | 133.0 | 130.5 | 0.019157 |
| branches | *P. sylvestris* | 128 | 5 | 164.1 | 161.3 | 0.017359 |
| branches | *P. sylvestris* | 128 | 6 | 110.7 | 108.2 | 0.023105 |
| branches | *P. sylvestris* | 128 | 7 | 135.6 | 133.3 | 0.017254 |
| branches | *P. sylvestris* | 138 | 2 | 143.7 | 140.9 | 0.019872 |
| branches | *P. sylvestris* | 138 | 3 | 134.3 | 132.0 | 0.017424 |
| branches | *P. sylvestris* | 138 | 4 | 134.5 | 131.8 | 0.020486 |
| branches | *P. sylvestris* | 138 | 5 | 134.0 | 131.5 | 0.019011 |
| branches | *P. sylvestris* | 138 | 6 | 137.2 | 134.7 | 0.018560 |
| branches | *P. sylvestris* | 138 | 7 | 136.4 | 134.0 | 0.017910 |
| branches | *P. sylvestris* | 138 | 8 | 134.1 | 131.3 | 0.021325 |
| branches | *P. abies* | 38 | 4 | 215.1 | 210.9 | 0.019915 |
| branches | *P. abies* | 38 | 7 | 224.5 | 220.2 | 0.019528 |
| branches | *P. abies* | 46 | 3 | 189.2 | 185.1 | 0.022150 |
| branches | *P. abies* | 46 | 6 | 204.2 | 200.2 | 0.019980 |
| branches | *P. abies* | 51 | 4 | 180.1 | 176.8 | 0.018665 |
| branches | *P. abies* | 51 | 5 | 210.7 | 206.5 | 0.020339 |
| branches | *P. abies* | 51 | 7 | 202.9 | 199.2 | 0.018574 |
| branches | *P. abies* | 69 | 6 | 224.0 | 219.9 | 0.018645 |
| branches | *P. abies* | 69 | 8 | 241.5 | 237.0 | 0.018987 |
| branches | *P. abies* | 73 | 4 | 238.0 | 233.6 | 0.018836 |
| branches | *P. abies* | 73 | 7 | 218.0 | 213.5 | 0.021077 |
| branches | *P. abies* | 78 | 2 | 236.1 | 231.4 | 0.020311 |
| branches | *P. abies* | 82 | 1 | 171.7 | 168.4 | 0.019596 |
| branches | *P. abies* | 82 | 3 | 194.1 | 190.5 | 0.018898 |
| branches | *P. abies* | 82 | 7 | 262.8 | 257.7 | 0.019790 |
| branches | *P. abies* | 82 | 8 | 183.0 | 179.9 | 0.017232 |
| branches | *P. abies* | 98 | 1 | 209.1 | 205.3 | 0.018509 |
| branches | *P. abies* | 98 | 4 | 195.8 | 191.9 | 0.020323 |
| branches | *P. abies* | 98 | 7 | 195.9 | 192.2 | 0.019251 |
| branches | *P. abies* | 140 | 2 | 188.5 | 185.0 | 0.018919 |
| branches | *P. abies* | 140 | 3 | 189.3 | 185.9 | 0.018289 |
| branches | *P. abies* | 140 | 6 | 201.2 | 197.1 | 0.020802 |
| branches | *P. abies* | 140 | 8 | 194.9 | 191.4 | 0.018286 |
| branches | *P. abies* | 169 | 1 | 164.7 | 161.6 | 0.019183 |
| branches | *P. abies* | 169 | 2 | 177.2 | 173.9 | 0.018976 |
| branches | *P. abies* | 169 | 3 | 166.6 | 163.4 | 0.019584 |
| branches | *P. abies* | 169 | 4 | 153.9 | 151.1 | 0.018531 |
| branches | *P. abies* | 169 | 5 | 180.6 | 176.9 | 0.020916 |
| branches | *P. abies* | 169 | 6 | 159.8 | 156.5 | 0.021086 |
| branches | *P. abies* | 169 | 8 | 176.6 | 173.2 | 0.019630 |
| foliage | *B. pendula* | 39 | 4 | 125.9 | 121.6 | 0.035362 |
| foliage | *B. pendula* | 45 | 6 | 160.5 | 155.7 | 0.030829 |
| foliage | *B. pendula* | 45 | 8 | 148.0 | 142.9 | 0.035689 |
| foliage | *B. pendula* | 58 | 5 | 114.8 | 111.8 | 0.026834 |
| foliage | *B. pendula* | 72 | 3 | 117.6 | 114.9 | 0.023499 |
| foliage | *B. pendula* | 86 | 2 | 128.6 | 125.0 | 0.028800 |
| foliage | *B. pendula* | 86 | 6 | 148.1 | 144.5 | 0.024913 |
| foliage | *B. pendula* | 89 | 1 | 118.4 | 115.2 | 0.027778 |
| foliage | *B. pendula* | 89 | 2 | 128.2 | 124.1 | 0.033038 |
| foliage | *B. pendula* | 89 | 3 | 141.7 | 138.4 | 0.023844 |
| foliage | *B. pendula* | 89 | 4 | 139.0 | 135.2 | 0.028107 |
| foliage | *B. pendula* | 89 | 5 | 113.4 | 110.5 | 0.026244 |
| foliage | *B. pendula* | 89 | 6 | 59.5 | 58.1 | 0.024096 |
| foliage | *B. pendula* | 89 | 7 | 142.1 | 138.1 | 0.028965 |
| foliage | *B. pendula* | 89 | 8 | 94.4 | 92.0 | 0.026087 |
| foliage | *B. pendula* | 134 | 1 | 139.3 | 135.2 | 0.030325 |
| foliage | *B. pendula* | 134 | 2 | 154.5 | 150.4 | 0.027261 |
| foliage | *B. pendula* | 134 | 3 | 148.4 | 143.7 | 0.032707 |
| foliage | *B. pendula* | 134 | 4 | 166.9 | 161.8 | 0.031520 |
| foliage | *B. pendula* | 134 | 5 | 157.2 | 152.7 | 0.029470 |
| foliage | *B. pendula* | 134 | 6 | 145.0 | 140.8 | 0.029830 |
| foliage | *B. pendula* | 134 | 7 | 143.1 | 138.4 | 0.033960 |
| foliage | *B. pendula* | 134 | 8 | 147.1 | 143.0 | 0.028671 |
| foliage | *B. pendula* | 171 | 1 | 134.3 | 129.5 | 0.037066 |
| foliage | *B. pendula* | 171 | 2 | 138.8 | 134.7 | 0.030438 |
| foliage | *B. pendula* | 171 | 3 | 127.3 | 123.8 | 0.028271 |
| foliage | *B. pendula* | 171 | 5 | 126.1 | 122.7 | 0.027710 |
| foliage | *B. pendula* | 171 | 6 | 121.5 | 117.8 | 0.031409 |
| foliage | *B. pendula* | 171 | 7 | 44.0 | 42.9 | 0.025641 |
| foliage | *B. pendula* | 171 | 8 | 64.5 | 62.8 | 0.027070 |
| foliage | *F. sylvatica* | 100 | 2 | 157.5 | 153.2 | 0.028068 |
| foliage | *F. sylvatica* | 100 | 5 | 163.2 | 159.0 | 0.026415 |
| foliage | *F. sylvatica* | 104 | 2 | 148.9 | 145.5 | 0.023368 |
| foliage | *F. sylvatica* | 108 | 6 | 160.5 | 156.5 | 0.025559 |
| foliage | *F. sylvatica* | 111 | 3 | 145.3 | 141.3 | 0.028309 |
| foliage | *F. sylvatica* | 111 | 7 | 129.6 | 126.1 | 0.027756 |
| foliage | *F. sylvatica* | 116 | 1 | 148.8 | 144.4 | 0.030471 |
| foliage | *F. sylvatica* | 116 | 8 | 156.2 | 150.9 | 0.035123 |
| foliage | *F. sylvatica* | 119 | 4 | 182.7 | 178.4 | 0.024103 |
| foliage | *F. sylvatica* | 129 | 1 | 55.3 | 53.7 | 0.029795 |
| foliage | *F. sylvatica* | 129 | 5 | 45.4 | 44.0 | 0.031818 |
| foliage | *F. sylvatica* | 130 | 1 | 165.1 | 161.0 | 0.025466 |
| foliage | *F. sylvatica* | 130 | 2 | 148.7 | 144.8 | 0.026934 |
| foliage | *F. sylvatica* | 130 | 5 | 165.2 | 160.9 | 0.026725 |
| foliage | *F. sylvatica* | 130 | 6 | 140.4 | 136.7 | 0.027067 |
| foliage | *F. sylvatica* | 130 | 7 | 146.0 | 142.6 | 0.023843 |
| foliage | *F. sylvatica* | 132 | 1 | 165.0 | 160.0 | 0.031250 |
| foliage | *F. sylvatica* | 132 | 3 | 151.1 | 147.9 | 0.021636 |
| foliage | *F. sylvatica* | 132 | 4 | 156.2 | 152.2 | 0.026281 |
| foliage | *F. sylvatica* | 132 | 7 | 154.0 | 149.7 | 0.028724 |
| foliage | *F. sylvatica* | 144 | 1 | 153.8 | 149.0 | 0.032215 |
| foliage | *F. sylvatica* | 144 | 2 | 155.0 | 151.0 | 0.026490 |
| foliage | *F. sylvatica* | 144 | 3 | 135.6 | 132.0 | 0.027273 |
| foliage | *F. sylvatica* | 144 | 6 | 143.1 | 138.9 | 0.030238 |
| foliage | *F. sylvatica* | 144 | 7 | 143.8 | 138.6 | 0.037518 |
| foliage | *F. sylvatica* | 170 | 2 | 143.4 | 139.4 | 0.028694 |
| foliage | *F. sylvatica* | 170 | 3 | 125.6 | 122.5 | 0.025306 |
| foliage | *F. sylvatica* | 170 | 4 | 139.4 | 135.1 | 0.031828 |
| foliage | *F. sylvatica* | 170 | 6 | 143.4 | 139.1 | 0.030913 |
| foliage | *F. sylvatica* | 170 | 8 | 143.6 | 139.6 | 0.028653 |
| foliage | *Q. robur* | 127 | 1 | 145.8 | 142.1 | 0.026038 |
| foliage | *Q. robur* | 127 | 2 | 153.8 | 150.1 | 0.024650 |
| foliage | *Q. robur* | 127 | 3 | 137.5 | 133.5 | 0.029963 |
| foliage | *Q. robur* | 127 | 6 | 149.5 | 145.2 | 0.029614 |
| foliage | *Q. robur* | 127 | 7 | 146.9 | 142.4 | 0.031601 |
| foliage | *Q. robur* | 127 | 8 | 160.7 | 155.9 | 0.030789 |
| foliage | *Q. robur* | 133 | 1 | 105.9 | 103.0 | 0.028155 |
| foliage | *Q. robur* | 133 | 2 | 106.9 | 103.8 | 0.029865 |
| foliage | *Q. robur* | 133 | 3 | 99.7 | 96.9 | 0.028896 |
| foliage | *Q. robur* | 133 | 4 | 95.5 | 92.9 | 0.027987 |
| foliage | *Q. robur* | 133 | 5 | 112.0 | 109.4 | 0.023766 |
| foliage | *Q. robur* | 133 | 6 | 100.2 | 97.3 | 0.029805 |
| foliage | *Q. robur* | 133 | 8 | 72.3 | 70.5 | 0.025532 |
| foliage | *Q. robur* | 136 | 6 | 128.6 | 124.6 | 0.032103 |
| foliage | *Q. robur* | 137 | 1 | 128.4 | 124.8 | 0.028846 |
| foliage | *Q. robur* | 137 | 2 | 132.1 | 129.0 | 0.024031 |
| foliage | *Q. robur* | 137 | 3 | 119.0 | 115.5 | 0.030303 |
| foliage | *Q. robur* | 137 | 4 | 132.2 | 128.9 | 0.025601 |
| foliage | *Q. robur* | 137 | 5 | 153.3 | 148.8 | 0.030242 |
| foliage | *Q. robur* | 137 | 7 | 107.2 | 104.1 | 0.029779 |
| foliage | *Q. robur* | 137 | 8 | 137.0 | 133.1 | 0.029301 |
| foliage | *Q. robur* | 141 | 1 | 137.5 | 133.5 | 0.029963 |
| foliage | *Q. robur* | 141 | 2 | 135.1 | 131.4 | 0.028158 |
| foliage | *Q. robur* | 141 | 4 | 133.5 | 129.7 | 0.029298 |
| foliage | *Q. robur* | 141 | 5 | 146.6 | 142.9 | 0.025892 |
| foliage | *Q. robur* | 141 | 7 | 135.0 | 130.6 | 0.033691 |
| foliage | *Q. robur* | 143 | 1 | 120.2 | 117.1 | 0.026473 |
| foliage | *Q. robur* | 143 | 2 | 130.8 | 126.5 | 0.033992 |
| foliage | *Q. robur* | 143 | 3 | 73.1 | 71.0 | 0.029577 |
| foliage | *Q. robur* | 143 | 4 | 136.7 | 132.8 | 0.029367 |
| foliage | *A. alba* | 105 | 3 | 368.2 | 363.1 | 0.014046 |
| foliage | *A. alba* | 105 | 5 | 256.5 | 253.4 | 0.012234 |
| foliage | *A. alba* | 105 | 8 | 257.0 | 254.0 | 0.011811 |
| foliage | *A. alba* | 109 | 2 | 316.6 | 312.9 | 0.011825 |
| foliage | *A. alba* | 109 | 3 | 266.0 | 262.7 | 0.012562 |
| foliage | *A. alba* | 109 | 4 | 259.6 | 256.1 | 0.013667 |
| foliage | *A. alba* | 109 | 6 | 259.4 | 256.4 | 0.011700 |
| foliage | *A. alba* | 112 | 1 | 308.2 | 304.8 | 0.011155 |
| foliage | *A. alba* | 112 | 2 | 274.3 | 271.1 | 0.011804 |
| foliage | *A. alba* | 112 | 7 | 294.9 | 290.9 | 0.013750 |
| foliage | *A. alba* | 112 | 8 | 338.4 | 334.2 | 0.012567 |
| foliage | *A. alba* | 113 | 2 | 455.2 | 448.8 | 0.014260 |
| foliage | *A. alba* | 113 | 3 | 373.5 | 368.6 | 0.013294 |
| foliage | *A. alba* | 113 | 6 | 347.9 | 343.1 | 0.013990 |
| foliage | *A. alba* | 115 | 1 | 308.1 | 302.8 | 0.017503 |
| foliage | *A. alba* | 115 | 2 | 285.9 | 281.9 | 0.014189 |
| foliage | *A. alba* | 115 | 4 | 247.1 | 244.7 | 0.009808 |
| foliage | *A. alba* | 115 | 5 | 320.8 | 316.1 | 0.014869 |
| foliage | *A. alba* | 121 | 2 | 322.9 | 318.5 | 0.013815 |
| foliage | *A. alba* | 121 | 4 | 306.4 | 302.3 | 0.013563 |
| foliage | *A. alba* | 121 | 5 | 305.1 | 301.3 | 0.012612 |
| foliage | *A. alba* | 122 | 1 | 318.3 | 314.2 | 0.013049 |
| foliage | *A. alba* | 122 | 5 | 259.4 | 255.8 | 0.014073 |
| foliage | *A. alba* | 122 | 6 | 246.4 | 243.2 | 0.013158 |
| foliage | *A. alba* | 124 | 2 | 310.7 | 306.9 | 0.012382 |
| foliage | *A. alba* | 124 | 5 | 393.7 | 388.7 | 0.012863 |
| foliage | *A. alba* | 124 | 6 | 392.3 | 387.0 | 0.013695 |
| foliage | *A. alba* | 125 | 1 | 297.4 | 293.6 | 0.012943 |
| foliage | *A. alba* | 125 | 2 | 385.7 | 380.9 | 0.012602 |
| foliage | *A. alba* | 125 | 7 | 394.4 | 388.8 | 0.014403 |
| foliage | *L. decidua* | 25 | 2 | 187.9 | 184.0 | 0.021196 |
| foliage | *L. decidua* | 25 | 5 | 125.7 | 124.0 | 0.013710 |
| foliage | *L. decidua* | 36 | 5 | 152.0 | 149.9 | 0.014009 |
| foliage | *L. decidua* | 36 | 6 | 134.1 | 132.4 | 0.012840 |
| foliage | *L. decidua* | 54 | 3 | 214.4 | 211.0 | 0.016114 |
| foliage | *L. decidua* | 54 | 4 | 205.4 | 202.3 | 0.015324 |
| foliage | *L. decidua* | 54 | 7 | 153.4 | 151.0 | 0.015894 |
| foliage | *L. decidua* | 59 | 4 | 125.0 | 123.2 | 0.014610 |
| foliage | *L. decidua* | 63 | 1 | 142.0 | 139.8 | 0.015737 |
| foliage | *L. decidua* | 75 | 1 | 113.2 | 111.6 | 0.014337 |
| foliage | *L. decidua* | 87 | 2 | 120.8 | 118.6 | 0.018550 |
| foliage | *L. decidua* | 87 | 4 | 99.9 | 98.3 | 0.016277 |
| foliage | *L. decidua* | 87 | 6 | 108.6 | 107.1 | 0.014006 |
| foliage | *L. decidua* | 87 | 7 | 109.1 | 107.4 | 0.015829 |
| foliage | *L. decidua* | 87 | 8 | 100.3 | 98.9 | 0.014156 |
| foliage | *L. decidua* | 99 | 2 | 144.1 | 142.7 | 0.009811 |
| foliage | *L. decidua* | 99 | 3 | 126.8 | 124.4 | 0.019293 |
| foliage | *L. decidua* | 99 | 4 | 148.8 | 146.2 | 0.017784 |
| foliage | *L. decidua* | 99 | 5 | 133.0 | 131.4 | 0.012177 |
| foliage | *L. decidua* | 99 | 8 | 118.8 | 116.9 | 0.016253 |
| foliage | *L. decidua* | 120 | 1 | 148.0 | 145.1 | 0.019986 |
| foliage | *L. decidua* | 120 | 2 | 147.0 | 145.0 | 0.013793 |
| foliage | *L. decidua* | 120 | 3 | 148.9 | 146.9 | 0.013615 |
| foliage | *L. decidua* | 120 | 6 | 143.8 | 141.0 | 0.019858 |
| foliage | *L. decidua* | 120 | 7 | 159.8 | 156.9 | 0.018483 |
| foliage | *L. decidua* | 139 | 2 | 161.2 | 158.3 | 0.018320 |
| foliage | *L. decidua* | 139 | 3 | 144.8 | 142.1 | 0.019001 |
| foliage | *L. decidua* | 139 | 5 | 148.9 | 146.2 | 0.018468 |
| foliage | *L. decidua* | 139 | 6 | 115.6 | 113.8 | 0.015817 |
| foliage | *L. decidua* | 139 | 8 | 95.2 | 93.6 | 0.017094 |
| foliage | *A. glutinosa* | 20 | 4 | 124.2 | 120.8 | 0.028146 |
| foliage | *A. glutinosa* | 31 | 1 | 126.1 | 122.0 | 0.033607 |
| foliage | *A. glutinosa* | 31 | 2 | 135.1 | 130.3 | 0.036838 |
| foliage | *A. glutinosa* | 31 | 3 | 131.5 | 127.6 | 0.030564 |
| foliage | *A. glutinosa* | 31 | 6 | 129.3 | 125.2 | 0.032748 |
| foliage | *A. glutinosa* | 50 | 3 | 171.2 | 165.9 | 0.031947 |
| foliage | *A. glutinosa* | 50 | 4 | 164.1 | 158.3 | 0.036639 |
| foliage | *A. glutinosa* | 50 | 7 | 108.0 | 104.9 | 0.029552 |
| foliage | *A. glutinosa* | 55 | 4 | 111.7 | 108.2 | 0.032348 |
| foliage | *A. glutinosa* | 55 | 6 | 128.2 | 123.7 | 0.036378 |
| foliage | *A. glutinosa* | 55 | 7 | 105.9 | 102.4 | 0.034180 |
| foliage | *A. glutinosa* | 55 | 8 | 112.0 | 107.6 | 0.040892 |
| foliage | *A. glutinosa* | 66 | 3 | 129.3 | 124.0 | 0.042742 |
| foliage | *A. glutinosa* | 66 | 5 | 145.3 | 139.4 | 0.042324 |
| foliage | *A. glutinosa* | 66 | 7 | 133.2 | 128.7 | 0.034965 |
| foliage | *A. glutinosa* | 66 | 8 | 124.0 | 119.9 | 0.034195 |
| foliage | *A. glutinosa* | 102 | 1 | 126.1 | 122.1 | 0.032760 |
| foliage | *A. glutinosa* | 102 | 2 | 137.0 | 131.9 | 0.038666 |
| foliage | *A. glutinosa* | 102 | 4 | 121.7 | 116.7 | 0.042845 |
| foliage | *A. glutinosa* | 102 | 5 | 141.1 | 137.5 | 0.026182 |
| foliage | *A. glutinosa* | 102 | 6 | 125.9 | 122.1 | 0.031122 |
| foliage | *A. glutinosa* | 102 | 7 | 129.9 | 126.4 | 0.027690 |
| foliage | *A. glutinosa* | 102 | 8 | 128.6 | 123.7 | 0.039612 |
| foliage | *A. glutinosa* | 135 | 1 | 135.8 | 132.1 | 0.028009 |
| foliage | *A. glutinosa* | 135 | 2 | 105.7 | 102.9 | 0.027211 |
| foliage | *A. glutinosa* | 135 | 3 | 119.9 | 116.2 | 0.031842 |
| foliage | *A. glutinosa* | 135 | 4 | 135.2 | 130.7 | 0.034430 |
| foliage | *A. glutinosa* | 135 | 6 | 125.0 | 121.0 | 0.033058 |
| foliage | *A. glutinosa* | 135 | 7 | 108.1 | 104.4 | 0.035441 |
| foliage | *A. glutinosa* | 135 | 8 | 28.1 | 27.4 | 0.025547 |
| foliage | *P. sylvestris* | 26 | 6 | 63.7 | 63.0 | 0.011111 |
| foliage | *P. sylvestris* | 40 | 1 | 48.1 | 47.6 | 0.010504 |
| foliage | *P. sylvestris* | 40 | 6 | 64.3 | 63.6 | 0.011006 |
| foliage | *P. sylvestris* | 42 | 3 | 49.0 | 48.3 | 0.014493 |
| foliage | *P. sylvestris* | 42 | 4 | 60.3 | 59.7 | 0.010050 |
| foliage | *P. sylvestris* | 47 | 3 | 44.5 | 43.8 | 0.015982 |
| foliage | *P. sylvestris* | 47 | 6 | 46.9 | 46.2 | 0.015152 |
| foliage | *P. sylvestris* | 59 | 3 | 57.7 | 57.0 | 0.012281 |
| foliage | *P. sylvestris* | 60 | 2 | 134.1 | 132.4 | 0.012840 |
| foliage | *P. sylvestris* | 60 | 4 | 172.9 | 171.0 | 0.011111 |
| foliage | *P. sylvestris* | 60 | 5 | 137.6 | 136.1 | 0.011021 |
| foliage | *P. sylvestris* | 60 | 7 | 80.5 | 79.4 | 0.013854 |
| foliage | *P. sylvestris* | 60 | 8 | 60.9 | 60.1 | 0.013311 |
| foliage | *P. sylvestris* | 62 | 5 | 43.1 | 42.6 | 0.011737 |
| foliage | *P. sylvestris* | 62 | 6 | 44.9 | 44.3 | 0.013544 |
| foliage | *P. sylvestris* | 76 | 2 | 46.5 | 45.9 | 0.013072 |
| foliage | *P. sylvestris* | 76 | 3 | 38.3 | 37.8 | 0.013228 |
| foliage | *P. sylvestris* | 76 | 5 | 36.1 | 35.7 | 0.011204 |
| foliage | *P. sylvestris* | 80 | 1 | 70.1 | 69.2 | 0.013006 |
| foliage | *P. sylvestris* | 80 | 4 | 69.8 | 68.8 | 0.014535 |
| foliage | *P. sylvestris* | 88 | 3 | 176.8 | 174.6 | 0.012600 |
| foliage | *P. sylvestris* | 101 | 3 | 103.4 | 102.0 | 0.013725 |
| foliage | *P. sylvestris* | 101 | 5 | 109.0 | 107.6 | 0.013011 |
| foliage | *P. sylvestris* | 101 | 6 | 108.2 | 106.8 | 0.013109 |
| foliage | *P. sylvestris* | 101 | 7 | 111.5 | 110.2 | 0.011797 |
| foliage | *P. sylvestris* | 101 | 8 | 100.6 | 99.2 | 0.014113 |
| foliage | *P. sylvestris* | 138 | 1 | 105.1 | 103.6 | 0.014479 |
| foliage | *P. sylvestris* | 138 | 2 | 94.5 | 93.7 | 0.008538 |
| foliage | *P. sylvestris* | 138 | 4 | 98.5 | 97.1 | 0.014418 |
| foliage | *P. sylvestris* | 138 | 7 | 104.9 | 103.5 | 0.013527 |
| foliage | *P. abies* | 28 | 6 | 389.2 | 383.2 | 0.015658 |
| foliage | *P. abies* | 28 | 7 | 305.5 | 301.7 | 0.012595 |
| foliage | *P. abies* | 38 | 2 | 283.2 | 280.2 | 0.010707 |
| foliage | *P. abies* | 38 | 3 | 345.6 | 341.5 | 0.012006 |
| foliage | *P. abies* | 38 | 5 | 298.5 | 294.4 | 0.013927 |
| foliage | *P. abies* | 38 | 6 | 340.0 | 335.8 | 0.012507 |
| foliage | *P. abies* | 51 | 3 | 282.5 | 279.4 | 0.011095 |
| foliage | *P. abies* | 51 | 6 | 303.2 | 299.1 | 0.013708 |
| foliage | *P. abies* | 52 | 5 | 255.7 | 252.9 | 0.011072 |
| foliage | *P. abies* | 69 | 2 | 405.2 | 400.1 | 0.012747 |
| foliage | *P. abies* | 69 | 3 | 350.9 | 346.1 | 0.013869 |
| foliage | *P. abies* | 71 | 2 | 281.6 | 277.3 | 0.015507 |
| foliage | *P. abies* | 71 | 5 | 475.2 | 468.0 | 0.015385 |
| foliage | *P. abies* | 73 | 1 | 375.8 | 370.7 | 0.013758 |
| foliage | *P. abies* | 78 | 6 | 276.7 | 273.3 | 0.012441 |
| foliage | *P. abies* | 79 | 1 | 255.4 | 252.2 | 0.012688 |
| foliage | *P. abies* | 81 | 3 | 263.5 | 260.4 | 0.011905 |
| foliage | *P. abies* | 81 | 8 | 281.6 | 277.8 | 0.013679 |
| foliage | *P. abies* | 82 | 1 | 352.1 | 348.0 | 0.011782 |
| foliage | *P. abies* | 82 | 2 | 317.9 | 313.8 | 0.013066 |
| foliage | *P. abies* | 92 | 1 | 381.6 | 375.8 | 0.015434 |
| foliage | *P. abies* | 92 | 2 | 322.5 | 317.7 | 0.015109 |
| foliage | *P. abies* | 92 | 5 | 400.5 | 393.6 | 0.017530 |
| foliage | *P. abies* | 97 | 3 | 438.2 | 430.8 | 0.017177 |
| foliage | *P. abies* | 97 | 4 | 230.6 | 227.2 | 0.014965 |
| foliage | *P. abies* | 98 | 1 | 418.2 | 412.3 | 0.014310 |
| foliage | *P. abies* | 98 | 3 | 412.3 | 405.9 | 0.015767 |
| foliage | *P. abies* | 98 | 4 | 354.0 | 348.2 | 0.016657 |
| foliage | *P. abies* | 98 | 6 | 295.1 | 291.0 | 0.014089 |
| foliage | *P. abies* | 98 | 7 | 344.5 | 339.5 | 0.014728 |
| wood | *B. pendula* | 30 | 1 | 1552.0 | 1541.8 | 0.006616 |
| wood | *B. pendula* | 30 | 1 | 770.0 | 765.1 | 0.006404 |
| wood | *B. pendula* | 30 | 2 | 938.8 | 931.8 | 0.007512 |
| wood | *B. pendula* | 30 | 2 | 511.5 | 507.1 | 0.008677 |
| wood | *B. pendula* | 30 | 3 | 858.5 | 852.7 | 0.006802 |
| wood | *B. pendula* | 30 | 3 | 459.9 | 456.5 | 0.007448 |
| wood | *B. pendula* | 30 | 4 | 1110.7 | 1102.5 | 0.007438 |
| wood | *B. pendula* | 30 | 5 | 404.3 | 401.7 | 0.006472 |
| wood | *B. pendula* | 30 | 7 | 648.3 | 644.1 | 0.006521 |
| wood | *B. pendula* | 30 | 7 | 367.9 | 365.1 | 0.007669 |
| wood | *B. pendula* | 45 | 1 | 1107.9 | 1099.5 | 0.007640 |
| wood | *B. pendula* | 45 | 2 | 1224.4 | 1216.0 | 0.006908 |
| wood | *B. pendula* | 45 | 4 | 1105.4 | 1097.8 | 0.006923 |
| wood | *B. pendula* | 45 | 5 | 986.6 | 980.1 | 0.006632 |
| wood | *B. pendula* | 45 | 5 | 535.4 | 531.8 | 0.006769 |
| wood | *B. pendula* | 45 | 7 | 695.5 | 690.3 | 0.007533 |
| wood | *B. pendula* | 45 | 8 | 529.6 | 525.0 | 0.008762 |
| wood | *B. pendula* | 45 | 8 | 279.8 | 277.4 | 0.008652 |
| wood | *B. pendula* | 46 | 3 | 696.0 | 691.0 | 0.007236 |
| wood | *B. pendula* | 58 | 2 | 1107.9 | 1099.9 | 0.007273 |
| wood | *B. pendula* | 58 | 3 | 1916.7 | 1903.4 | 0.006987 |
| wood | *B. pendula* | 58 | 4 | 1432.8 | 1422.3 | 0.007382 |
| wood | *B. pendula* | 58 | 5 | 1255.4 | 1246.5 | 0.007140 |
| wood | *B. pendula* | 58 | 5 | 704.6 | 699.6 | 0.007147 |
| wood | *B. pendula* | 58 | 6 | 677.2 | 672.1 | 0.007588 |
| wood | *B. pendula* | 58 | 7 | 907.3 | 900.9 | 0.007104 |
| wood | *B. pendula* | 58 | 7 | 907.9 | 901.5 | 0.007099 |
| wood | *B. pendula* | 64 | 4 | 1426.3 | 1415.9 | 0.007345 |
| wood | *B. pendula* | 72 | 1 | 900.7 | 894.3 | 0.007156 |
| wood | *B. pendula* | 72 | 1 | 454.0 | 450.5 | 0.007769 |
| wood | *B. pendula* | 72 | 3 | 289.3 | 287.2 | 0.007312 |
| wood | *B. pendula* | 72 | 4 | 231.6 | 229.9 | 0.007395 |
| wood | *B. pendula* | 72 | 5 | 444.2 | 440.9 | 0.007485 |
| wood | *B. pendula* | 86 | 1 | 248.5 | 246.7 | 0.007296 |
| wood | *B. pendula* | 86 | 2 | 5411.0 | 5369.5 | 0.007729 |
| wood | *B. pendula* | 86 | 2 | 2679.0 | 2658.5 | 0.007711 |
| wood | *B. pendula* | 86 | 3 | 1930.9 | 1916.5 | 0.007514 |
| wood | *B. pendula* | 86 | 4 | 3132.0 | 3108.4 | 0.007592 |
| wood | *B. pendula* | 86 | 4 | 1735.1 | 1721.3 | 0.008017 |
| wood | *B. pendula* | 86 | 5 | 3747.5 | 3719.7 | 0.007474 |
| wood | *B. pendula* | 86 | 5 | 2381.3 | 2362.9 | 0.007787 |
| wood | *B. pendula* | 86 | 7 | 3070.2 | 3046.8 | 0.007680 |
| wood | *B. pendula* | 86 | 7 | 1511.2 | 1499.7 | 0.007668 |
| wood | *B. pendula* | 86 | 8 | 2493.1 | 2474.6 | 0.007476 |
| wood | *B. pendula* | 86 | 8 | 1543.5 | 1532.1 | 0.007441 |
| wood | *B. pendula* | 89 | 1 | 348.1 | 345.7 | 0.006942 |
| wood | *B. pendula* | 89 | 1 | 175.5 | 174.2 | 0.007463 |
| wood | *B. pendula* | 89 | 2 | 49.2 | 48.8 | 0.008197 |
| wood | *B. pendula* | 89 | 2 | 114.4 | 113.2 | 0.010601 |
| wood | *B. pendula* | 89 | 2 | 349.3 | 346.6 | 0.007790 |
| wood | *B. pendula* | 89 | 2 | 406.3 | 403.1 | 0.007938 |
| wood | *B. pendula* | 89 | 2 | 496.9 | 493.1 | 0.007706 |
| wood | *B. pendula* | 89 | 2 | 181.2 | 179.7 | 0.008347 |
| wood | *B. pendula* | 89 | 2 | 263.8 | 261.6 | 0.008410 |
| wood | *B. pendula* | 89 | 2 | 326.8 | 324.3 | 0.007709 |
| wood | *B. pendula* | 89 | 3 | 169.2 | 167.8 | 0.008343 |
| wood | *B. pendula* | 89 | 3 | 120.2 | 119.1 | 0.009236 |
| wood | *B. pendula* | 89 | 4 | 196.2 | 194.5 | 0.008740 |
| wood | *B. pendula* | 89 | 4 | 78.1 | 77.5 | 0.007742 |
| wood | *B. pendula* | 89 | 5 | 136.2 | 135.2 | 0.007396 |
| wood | *B. pendula* | 89 | 7 | 81.6 | 80.9 | 0.008653 |
| wood | *B. pendula* | 134 | 1 | 127.0 | 126.0 | 0.007937 |
| wood | *B. pendula* | 134 | 1 | 3126.8 | 3105.0 | 0.007021 |
| wood | *B. pendula* | 134 | 1 | 1900.9 | 1886.5 | 0.007633 |
| wood | *B. pendula* | 134 | 2 | 108.8 | 107.2 | 0.014925 |
| wood | *B. pendula* | 134 | 2 | 2173.4 | 2157.1 | 0.007556 |
| wood | *B. pendula* | 134 | 3 | 103.5 | 102.6 | 0.008772 |
| wood | *B. pendula* | 134 | 4 | 20.9 | 20.8 | 0.004808 |
| wood | *B. pendula* | 134 | 4 | 134.8 | 133.7 | 0.008227 |
| wood | *B. pendula* | 134 | 4 | 252.5 | 250.5 | 0.007984 |
| wood | *B. pendula* | 134 | 4 | 1060.5 | 1052.3 | 0.007792 |
| wood | *B. pendula* | 134 | 4 | 1259.9 | 1250.7 | 0.007356 |
| wood | *B. pendula* | 134 | 4 | 1267.5 | 1258.2 | 0.007392 |
| wood | *B. pendula* | 134 | 4 | 1634.4 | 1621.6 | 0.007893 |
| wood | *B. pendula* | 134 | 4 | 2079.7 | 2063.1 | 0.008046 |
| wood | *B. pendula* | 134 | 4 | 2588.1 | 2569.0 | 0.007435 |
| wood | *B. pendula* | 134 | 4 | 293.9 | 291.6 | 0.007888 |
| wood | *B. pendula* | 134 | 4 | 421.3 | 418.0 | 0.007895 |
| wood | *B. pendula* | 134 | 4 | 644.8 | 640.1 | 0.007343 |
| wood | *B. pendula* | 134 | 5 | 90.4 | 89.7 | 0.007804 |
| wood | *B. pendula* | 134 | 5 | 1495.3 | 1485.1 | 0.006868 |
| wood | *B. pendula* | 134 | 5 | 901.5 | 895.0 | 0.007263 |
| wood | *B. pendula* | 134 | 6 | 1220.9 | 1212.2 | 0.007177 |
| wood | *B. pendula* | 134 | 7 | 106.6 | 105.8 | 0.007561 |
| wood | *B. pendula* | 134 | 7 | 747.2 | 741.7 | 0.007415 |
| wood | *B. pendula* | 134 | 8 | 648.3 | 643.5 | 0.007459 |
| wood | *B. pendula* | 171 | 1 | 135.4 | 134.2 | 0.008942 |
| wood | *B. pendula* | 171 | 1 | 40.8 | 40.5 | 0.007407 |
| wood | *B. pendula* | 171 | 2 | 90.0 | 89.3 | 0.007839 |
| wood | *B. pendula* | 171 | 5 | 14.8 | 14.6 | 0.013699 |
| wood | *F. sylvatica* | 18 | 1 | 2205.2 | 2187.3 | 0.008184 |
| wood | *F. sylvatica* | 18 | 2 | 1543.5 | 1530.8 | 0.008296 |
| wood | *F. sylvatica* | 18 | 3 | 2366.7 | 2348.8 | 0.007621 |
| wood | *F. sylvatica* | 18 | 4 | 2513.5 | 2492.7 | 0.008344 |
| wood | *F. sylvatica* | 18 | 4 | 1341.1 | 1331.0 | 0.007588 |
| wood | *F. sylvatica* | 18 | 7 | 1338.9 | 1328.0 | 0.008208 |
| wood | *F. sylvatica* | 35 | 5 | 3880.4 | 3848.6 | 0.008263 |
| wood | *F. sylvatica* | 37 | 2 | 777.3 | 772.7 | 0.005953 |
| wood | *F. sylvatica* | 37 | 3 | 633.6 | 629.5 | 0.006513 |
| wood | *F. sylvatica* | 41 | 1 | 1256.2 | 1246.9 | 0.007458 |
| wood | *F. sylvatica* | 65 | 1 | 987.0 | 979.9 | 0.007246 |
| wood | *F. sylvatica* | 65 | 7 | 1723.8 | 1709.8 | 0.008188 |
| wood | *F. sylvatica* | 93 | 4 | 2674.1 | 2653.6 | 0.007725 |
| wood | *F. sylvatica* | 93 | 5 | 1309.2 | 1299.3 | 0.007619 |
| wood | *F. sylvatica* | 93 | 7 | 2250.6 | 2233.7 | 0.007566 |
| wood | *F. sylvatica* | 93 | 8 | 1591.2 | 1579.6 | 0.007344 |
| wood | *F. sylvatica* | 100 | 1 | 542.1 | 538.0 | 0.007621 |
| wood | *F. sylvatica* | 100 | 1 | 8808.5 | 8738.5 | 0.008011 |
| wood | *F. sylvatica* | 100 | 3 | 5864.7 | 5816.2 | 0.008339 |
| wood | *F. sylvatica* | 100 | 3 | 595.5 | 590.4 | 0.008638 |
| wood | *F. sylvatica* | 100 | 4 | 4324.8 | 4289.9 | 0.008135 |
| wood | *F. sylvatica* | 100 | 4 | 2659.1 | 2634.9 | 0.009184 |
| wood | *F. sylvatica* | 104 | 2 | 4784.5 | 4746.0 | 0.008112 |
| wood | *F. sylvatica* | 104 | 3 | 193.3 | 191.2 | 0.010983 |
| wood | *F. sylvatica* | 104 | 4 | 20.0 | 19.8 | 0.010101 |
| wood | *F. sylvatica* | 104 | 4 | 134.4 | 133.4 | 0.007496 |
| wood | *F. sylvatica* | 104 | 4 | 397.2 | 394.6 | 0.006589 |
| wood | *F. sylvatica* | 104 | 4 | 3042.1 | 3020.7 | 0.007084 |
| wood | *F. sylvatica* | 104 | 4 | 1749.3 | 1737.2 | 0.006965 |
| wood | *F. sylvatica* | 104 | 4 | 1871.2 | 1856.9 | 0.007701 |
| wood | *F. sylvatica* | 104 | 4 | 2093.1 | 2077.5 | 0.007509 |
| wood | *F. sylvatica* | 104 | 5 | 196.3 | 194.6 | 0.008736 |
| wood | *F. sylvatica* | 108 | 1 | 1678.7 | 1667.6 | 0.006656 |
| wood | *F. sylvatica* | 108 | 4 | 137.6 | 136.2 | 0.010279 |
| wood | *F. sylvatica* | 108 | 4 | 195.9 | 194.2 | 0.008754 |
| wood | *F. sylvatica* | 108 | 4 | 255.4 | 253.7 | 0.006701 |
| wood | *F. sylvatica* | 108 | 4 | 2033.8 | 2019.2 | 0.007231 |
| wood | *F. sylvatica* | 108 | 4 | 2143.4 | 2126.1 | 0.008137 |
| wood | *F. sylvatica* | 108 | 4 | 401.2 | 398.1 | 0.007787 |
| wood | *F. sylvatica* | 108 | 4 | 456.9 | 453.9 | 0.006609 |
| wood | *F. sylvatica* | 108 | 8 | 565.8 | 561.1 | 0.008376 |
| wood | *F. sylvatica* | 111 | 2 | 582.9 | 579.1 | 0.006562 |
| wood | *F. sylvatica* | 111 | 4 | 18.7 | 18.5 | 0.010811 |
| wood | *F. sylvatica* | 111 | 4 | 824.4 | 818.7 | 0.006962 |
| wood | *F. sylvatica* | 116 | 3 | 179.4 | 177.9 | 0.008432 |
| wood | *F. sylvatica* | 116 | 3 | 407.6 | 404.7 | 0.007166 |
| wood | *F. sylvatica* | 116 | 3 | 6959.5 | 6906.0 | 0.007747 |
| wood | *F. sylvatica* | 116 | 3 | 513.3 | 509.2 | 0.008052 |
| wood | *F. sylvatica* | 116 | 3 | 816.1 | 809.8 | 0.007780 |
| wood | *F. sylvatica* | 116 | 3 | 1380.8 | 1371.0 | 0.007148 |
| wood | *F. sylvatica* | 116 | 3 | 1989.2 | 1971.5 | 0.008978 |
| wood | *F. sylvatica* | 116 | 3 | 2028.9 | 2013.5 | 0.007648 |
| wood | *F. sylvatica* | 116 | 5 | 243.2 | 241.5 | 0.007039 |
| wood | *F. sylvatica* | 119 | 2 | 543.8 | 539.3 | 0.008344 |
| wood | *F. sylvatica* | 119 | 3 | 7026.5 | 6973.2 | 0.007644 |
| wood | *F. sylvatica* | 119 | 4 | 47.6 | 47.0 | 0.012766 |
| wood | *F. sylvatica* | 119 | 4 | 149.3 | 148.3 | 0.006743 |
| wood | *F. sylvatica* | 119 | 4 | 325.3 | 322.9 | 0.007433 |
| wood | *F. sylvatica* | 119 | 4 | 388.6 | 385.7 | 0.007519 |
| wood | *F. sylvatica* | 119 | 4 | 1177.5 | 1167.6 | 0.008479 |
| wood | *F. sylvatica* | 119 | 7 | 2595.5 | 2575.2 | 0.007883 |
| wood | *F. sylvatica* | 119 | 8 | 3084.4 | 3063.2 | 0.006921 |
| wood | *F. sylvatica* | 130 | 4 | 12.6 | 12.4 | 0.016129 |
| wood | *F. sylvatica* | 130 | 4 | 91.5 | 90.9 | 0.006601 |
| wood | *F. sylvatica* | 131 | 4 | 16.9 | 16.8 | 0.005952 |
| wood | *F. sylvatica* | 131 | 4 | 523.0 | 519.5 | 0.006737 |
| wood | *F. sylvatica* | 131 | 4 | 584.6 | 580.4 | 0.007236 |
| wood | *F. sylvatica* | 131 | 4 | 800.4 | 794.8 | 0.007046 |
| wood | *F. sylvatica* | 131 | 4 | 862.0 | 856.1 | 0.006892 |
| wood | *F. sylvatica* | 131 | 4 | 1307.2 | 1297.8 | 0.007243 |
| wood | *F. sylvatica* | 131 | 6 | 2710.7 | 2689.2 | 0.007995 |
| wood | *F. sylvatica* | 131 | 8 | 1016.4 | 1009.6 | 0.006735 |
| wood | *F. sylvatica* | 131 | 8 | 560.0 | 556.1 | 0.007013 |
| wood | *F. sylvatica* | 132 | 3 | 8798.3 | 8727.7 | 0.008089 |
| wood | *F. sylvatica* | 132 | 5 | 5871.9 | 5826.0 | 0.007878 |
| wood | *F. sylvatica* | 132 | 6 | 4966.8 | 4927.3 | 0.008017 |
| wood | *F. sylvatica* | 132 | 8 | 207.5 | 206.1 | 0.006793 |
| wood | *F. sylvatica* | 144 | 2 | 1507.3 | 1495.9 | 0.007621 |
| wood | *F. sylvatica* | 144 | 4 | 43.4 | 43.0 | 0.009302 |
| wood | *F. sylvatica* | 144 | 4 | 89.6 | 89.0 | 0.006742 |
| wood | *F. sylvatica* | 144 | 4 | 1287.1 | 1278.5 | 0.006727 |
| wood | *F. sylvatica* | 144 | 4 | 1690.6 | 1677.7 | 0.007689 |
| wood | *F. sylvatica* | 144 | 4 | 1770.5 | 1758.6 | 0.006767 |
| wood | *F. sylvatica* | 144 | 4 | 1829.9 | 1816.2 | 0.007543 |
| wood | *F. sylvatica* | 144 | 4 | 773.2 | 767.8 | 0.007033 |
| wood | *F. sylvatica* | 144 | 6 | 1185.0 | 1176.0 | 0.007653 |
| wood | *F. sylvatica* | 170 | 3 | 2915.9 | 2892.5 | 0.008090 |
| wood | *F. sylvatica* | 170 | 4 | 4260.7 | 4226.5 | 0.008092 |
| wood | *F. sylvatica* | 170 | 6 | 1463.1 | 1451.5 | 0.007992 |
| wood | *F. sylvatica* | 170 | 7 | 1139.8 | 1132.0 | 0.006890 |
| wood | *Q. robur* | 24 | 1 | 1143.0 | 1130.1 | 0.011415 |
| wood | *Q. robur* | 24 | 5 | 12.3 | 12.2 | 0.008197 |
| wood | *Q. robur* | 24 | 5 | 339.7 | 336.2 | 0.010410 |
| wood | *Q. robur* | 24 | 8 | 154.6 | 152.9 | 0.011118 |
| wood | *Q. robur* | 29 | 2 | 314.0 | 310.3 | 0.011924 |
| wood | *Q. robur* | 29 | 2 | 3996.0 | 3945.4 | 0.012825 |
| wood | *Q. robur* | 29 | 4 | 474.5 | 468.1 | 0.013672 |
| wood | *Q. robur* | 29 | 4 | 1420.9 | 1404.7 | 0.011533 |
| wood | *Q. robur* | 29 | 5 | 297.0 | 293.8 | 0.010892 |
| wood | *Q. robur* | 29 | 6 | 2797.2 | 2766.9 | 0.010951 |
| wood | *Q. robur* | 29 | 8 | 1968.4 | 1945.9 | 0.011563 |
| wood | *Q. robur* | 29 | 8 | 1035.9 | 1024.7 | 0.010930 |
| wood | *Q. robur* | 32 | 1 | 18.4 | 18.3 | 0.005464 |
| wood | *Q. robur* | 32 | 1 | 177.0 | 175.8 | 0.006826 |
| wood | *Q. robur* | 32 | 4 | 13.7 | 13.5 | 0.014815 |
| wood | *Q. robur* | 32 | 8 | 14.3 | 14.1 | 0.014184 |
| wood | *Q. robur* | 34 | 3 | 1342.9 | 1327.9 | 0.011296 |
| wood | *Q. robur* | 34 | 3 | 1066.1 | 1054.3 | 0.011192 |
| wood | *Q. robur* | 34 | 7 | 666.7 | 659.8 | 0.010458 |
| wood | *Q. robur* | 43 | 3 | 892.0 | 880.8 | 0.012716 |
| wood | *Q. robur* | 43 | 5 | 768.6 | 759.3 | 0.012248 |
| wood | *Q. robur* | 43 | 6 | 927.3 | 917.4 | 0.010791 |
| wood | *Q. robur* | 43 | 6 | 510.6 | 505.0 | 0.011089 |
| wood | *Q. robur* | 43 | 8 | 207.1 | 204.7 | 0.011724 |
| wood | *Q. robur* | 48 | 4 | 495.8 | 489.3 | 0.013284 |
| wood | *Q. robur* | 49 | 2 | 1604.5 | 1585.4 | 0.012047 |
| wood | *Q. robur* | 49 | 4 | 116.2 | 115.2 | 0.008681 |
| wood | *Q. robur* | 49 | 6 | 1284.0 | 1269.5 | 0.011422 |
| wood | *Q. robur* | 61 | 1 | 373.0 | 368.6 | 0.011937 |
| wood | *Q. robur* | 61 | 2 | 302.1 | 298.5 | 0.012060 |
| wood | *Q. robur* | 61 | 6 | 3536.1 | 3497.1 | 0.011152 |
| wood | *Q. robur* | 61 | 7 | 105.5 | 104.3 | 0.011505 |
| wood | *Q. robur* | 61 | 8 | 237.2 | 234.5 | 0.011514 |
| wood | *Q. robur* | 64 | 4 | 2146.2 | 2120.4 | 0.012168 |
| wood | *Q. robur* | 64 | 4 | 1180.4 | 1166.1 | 0.012263 |
| wood | *Q. robur* | 64 | 5 | 1593.1 | 1572.7 | 0.012971 |
| wood | *Q. robur* | 64 | 6 | 1838.5 | 1819.0 | 0.010720 |
| wood | *Q. robur* | 68 | 1 | 271.7 | 268.4 | 0.012295 |
| wood | *Q. robur* | 68 | 1 | 1993.5 | 1967.5 | 0.013215 |
| wood | *Q. robur* | 68 | 3 | 216.3 | 213.3 | 0.014065 |
| wood | *Q. robur* | 77 | 1 | 809.0 | 798.6 | 0.013023 |
| wood | *Q. robur* | 77 | 5 | 3463.3 | 3416.1 | 0.013817 |
| wood | *Q. robur* | 77 | 8 | 95.5 | 95.3 | 0.002099 |
| wood | *Q. robur* | 78 | 1 | 3159.5 | 3123.2 | 0.011623 |
| wood | *Q. robur* | 79 | 1 | 233.3 | 230.8 | 0.010832 |
| wood | *Q. robur* | 79 | 2 | 1366.2 | 1350.2 | 0.011850 |
| wood | *Q. robur* | 79 | 3 | 891.2 | 881.5 | 0.011004 |
| wood | *Q. robur* | 79 | 5 | 112.3 | 111.1 | 0.010801 |
| wood | *Q. robur* | 79 | 8 | 338.5 | 334.5 | 0.011958 |
| wood | *Q. robur* | 83 | 1 | 319.7 | 315.7 | 0.012670 |
| wood | *Q. robur* | 83 | 2 | 248.0 | 245.1 | 0.011832 |
| wood | *Q. robur* | 83 | 8 | 127.4 | 126.1 | 0.010309 |
| wood | *Q. robur* | 84 | 1 | 1281.8 | 1266.2 | 0.012320 |
| wood | *Q. robur* | 84 | 2 | 950.4 | 938.8 | 0.012356 |
| wood | *Q. robur* | 84 | 3 | 1703.0 | 1681.7 | 0.012666 |
| wood | *Q. robur* | 84 | 7 | 463.9 | 458.6 | 0.011557 |
| wood | *Q. robur* | 127 | 1 | 12.2 | 12.0 | 0.016667 |
| wood | *Q. robur* | 127 | 1 | 732.2 | 724.4 | 0.010768 |
| wood | *Q. robur* | 127 | 2 | 635.0 | 627.8 | 0.011469 |
| wood | *Q. robur* | 127 | 2 | 247.0 | 244.3 | 0.011052 |
| wood | *Q. robur* | 127 | 3 | 184.2 | 182.2 | 0.010977 |
| wood | *Q. robur* | 127 | 4 | 22.9 | 22.7 | 0.008811 |
| wood | *Q. robur* | 127 | 4 | 281.1 | 278.5 | 0.009336 |
| wood | *Q. robur* | 127 | 5 | 283.0 | 280.4 | 0.009272 |
| wood | *Q. robur* | 127 | 7 | 102.9 | 102.1 | 0.007835 |
| wood | *Q. robur* | 127 | 8 | 166.6 | 165.0 | 0.009697 |
| wood | *Q. robur* | 133 | 1 | 97.1 | 96.0 | 0.011458 |
| wood | *Q. robur* | 133 | 2 | 98.1 | 97.1 | 0.010299 |
| wood | *Q. robur* | 133 | 5 | 14.1 | 13.9 | 0.014388 |
| wood | *Q. robur* | 133 | 6 | 15.6 | 15.4 | 0.012987 |
| wood | *Q. robur* | 137 | 4 | 271.5 | 268.4 | 0.011550 |
| wood | *Q. robur* | 137 | 4 | 3344.5 | 3298.0 | 0.014099 |
| wood | *Q. robur* | 137 | 4 | 5500.0 | 5427.0 | 0.013451 |
| wood | *Q. robur* | 137 | 4 | 6095.1 | 6016.7 | 0.013030 |
| wood | *Q. robur* | 137 | 4 | 6122.3 | 6046.1 | 0.012603 |
| wood | *Q. robur* | 137 | 4 | 6569.4 | 6486.3 | 0.012812 |
| wood | *Q. robur* | 137 | 4 | 7927.7 | 7831.9 | 0.012232 |
| wood | *Q. robur* | 137 | 4 | 1322.5 | 1306.6 | 0.012169 |
| wood | *Q. robur* | 137 | 4 | 1485.6 | 1467.0 | 0.012679 |
| wood | *Q. robur* | 137 | 4 | 1632.0 | 1611.4 | 0.012784 |
| wood | *Q. robur* | 137 | 4 | 1678.1 | 1657.5 | 0.012428 |
| wood | *Q. robur* | 137 | 5 | 252.8 | 249.7 | 0.012415 |
| wood | *Q. robur* | 137 | 6 | 247.4 | 244.5 | 0.011861 |
| wood | *Q. robur* | 137 | 6 | 2775.6 | 2742.2 | 0.012180 |
| wood | *Q. robur* | 141 | 1 | 108.1 | 106.9 | 0.011225 |
| wood | *Q. robur* | 141 | 4 | 37.9 | 37.5 | 0.010667 |
| wood | *Q. robur* | 143 | 1 | 330.3 | 326.2 | 0.012569 |
| wood | *Q. robur* | 143 | 2 | 506.8 | 501.2 | 0.011173 |
| wood | *Q. robur* | 143 | 3 | 6786.5 | 6706.9 | 0.011868 |
| wood | *Q. robur* | 143 | 5 | 438.0 | 432.7 | 0.012249 |
| wood | *A. alba* | 105 | 4 | 317.3 | 313.5 | 0.012121 |
| wood | *A. alba* | 105 | 4 | 572.8 | 566.2 | 0.011657 |
| wood | *A. alba* | 105 | 4 | 582.4 | 575.6 | 0.011814 |
| wood | *A. alba* | 105 | 4 | 2467.8 | 2440.8 | 0.011062 |
| wood | *A. alba* | 105 | 4 | 2661.0 | 2631.0 | 0.011403 |
| wood | *A. alba* | 105 | 4 | 3159.6 | 3123.2 | 0.011655 |
| wood | *A. alba* | 105 | 4 | 5307.5 | 5250.3 | 0.010895 |
| wood | *A. alba* | 105 | 4 | 982.7 | 971.4 | 0.011633 |
| wood | *A. alba* | 105 | 4 | 1392.1 | 1375.0 | 0.012436 |
| wood | *A. alba* | 105 | 4 | 1629.1 | 1609.8 | 0.011989 |
| wood | *A. alba* | 109 | 1 | 129.4 | 127.9 | 0.011728 |
| wood | *A. alba* | 109 | 4 | 80.3 | 79.3 | 0.012610 |
| wood | *A. alba* | 109 | 4 | 1032.4 | 1019.9 | 0.012256 |
| wood | *A. alba* | 109 | 4 | 1152.7 | 1138.3 | 0.012650 |
| wood | *A. alba* | 109 | 4 | 525.3 | 518.8 | 0.012529 |
| wood | *A. alba* | 109 | 4 | 622.8 | 615.2 | 0.012354 |
| wood | *A. alba* | 109 | 6 | 68.4 | 67.5 | 0.013333 |
| wood | *A. alba* | 109 | 6 | 495.4 | 489.3 | 0.012467 |
| wood | *A. alba* | 109 | 7 | 372.8 | 368.5 | 0.011669 |
| wood | *A. alba* | 109 | 8 | 632.4 | 625.4 | 0.011193 |
| wood | *A. alba* | 112 | 4 | 4004.3 | 3960.2 | 0.011136 |
| wood | *A. alba* | 112 | 4 | 771.9 | 762.4 | 0.012461 |
| wood | *A. alba* | 112 | 4 | 1665.1 | 1647.0 | 0.010990 |
| wood | *A. alba* | 112 | 4 | 1817.0 | 1794.8 | 0.012369 |
| wood | *A. alba* | 112 | 4 | 2753.8 | 2722.7 | 0.011422 |
| wood | *A. alba* | 112 | 5 | 278.5 | 275.2 | 0.011991 |
| wood | *A. alba* | 112 | 6 | 193.9 | 191.3 | 0.013591 |
| wood | *A. alba* | 112 | 8 | 163.4 | 161.4 | 0.012392 |
| wood | *A. alba* | 113 | 1 | 298.9 | 295.3 | 0.012191 |
| wood | *A. alba* | 113 | 3 | 418.3 | 413.7 | 0.011119 |
| wood | *A. alba* | 113 | 3 | 432.3 | 427.1 | 0.012175 |
| wood | *A. alba* | 113 | 3 | 1621.8 | 1603.3 | 0.011539 |
| wood | *A. alba* | 113 | 3 | 1737.7 | 1718.3 | 0.011290 |
| wood | *A. alba* | 113 | 3 | 1890.2 | 1870.4 | 0.010586 |
| wood | *A. alba* | 113 | 3 | 2489.5 | 2461.3 | 0.011457 |
| wood | *A. alba* | 113 | 3 | 666.2 | 658.5 | 0.011693 |
| wood | *A. alba* | 113 | 3 | 808.1 | 797.7 | 0.013037 |
| wood | *A. alba* | 113 | 3 | 837.7 | 827.8 | 0.011959 |
| wood | *A. alba* | 113 | 3 | 1130.3 | 1117.1 | 0.011816 |
| wood | *A. alba* | 113 | 4 | 222.3 | 219.6 | 0.012295 |
| wood | *A. alba* | 113 | 5 | 213.3 | 210.7 | 0.012340 |
| wood | *A. alba* | 113 | 6 | 1212.2 | 1197.8 | 0.012022 |
| wood | *A. alba* | 113 | 7 | 155.1 | 153.2 | 0.012402 |
| wood | *A. alba* | 113 | 8 | 73.3 | 72.5 | 0.011034 |
| wood | *A. alba* | 113 | 8 | 553.2 | 546.3 | 0.012630 |
| wood | *A. alba* | 115 | 2 | 757.0 | 747.3 | 0.012980 |
| wood | *A. alba* | 115 | 3 | 266.1 | 262.8 | 0.012557 |
| wood | *A. alba* | 115 | 4 | 240.0 | 237.3 | 0.011378 |
| wood | *A. alba* | 115 | 4 | 476.4 | 470.7 | 0.012110 |
| wood | *A. alba* | 115 | 4 | 627.1 | 620.1 | 0.011289 |
| wood | *A. alba* | 115 | 5 | 294.9 | 291.4 | 0.012011 |
| wood | *A. alba* | 115 | 6 | 241.7 | 239.1 | 0.010874 |
| wood | *A. alba* | 115 | 8 | 8.4 | 8.3 | 0.012048 |
| wood | *A. alba* | 115 | 8 | 159.4 | 157.5 | 0.012063 |
| wood | *A. alba* | 121 | 1 | 148.1 | 146.4 | 0.011612 |
| wood | *A. alba* | 121 | 2 | 57.6 | 56.5 | 0.019469 |
| wood | *A. alba* | 121 | 4 | 1147.4 | 1134.0 | 0.011817 |
| wood | *A. alba* | 121 | 4 | 1154.9 | 1142.3 | 0.011030 |
| wood | *A. alba* | 121 | 4 | 1161.8 | 1148.3 | 0.011757 |
| wood | *A. alba* | 121 | 4 | 433.9 | 428.6 | 0.012366 |
| wood | *A. alba* | 121 | 4 | 919.1 | 908.8 | 0.011334 |
| wood | *A. alba* | 121 | 7 | 305.7 | 302.2 | 0.011582 |
| wood | *A. alba* | 121 | 8 | 32.4 | 32.0 | 0.012500 |
| wood | *A. alba* | 121 | 8 | 396.9 | 392.2 | 0.011984 |
| wood | *A. alba* | 121 | 8 | 183.4 | 181.3 | 0.011583 |
| wood | *A. alba* | 122 | 3 | 3043.7 | 3008.4 | 0.011734 |
| wood | *A. alba* | 122 | 3 | 363.3 | 358.7 | 0.012824 |
| wood | *A. alba* | 122 | 4 | 3883.1 | 3840.6 | 0.011066 |
| wood | *A. alba* | 122 | 4 | 1822.3 | 1799.3 | 0.012783 |
| wood | *A. alba* | 122 | 4 | 192.2 | 189.8 | 0.012645 |
| wood | *A. alba* | 122 | 4 | 5778.0 | 5714.7 | 0.011077 |
| wood | *A. alba* | 122 | 4 | 4982.7 | 4926.1 | 0.011490 |
| wood | *A. alba* | 122 | 4 | 5086.9 | 5031.1 | 0.011091 |
| wood | *A. alba* | 122 | 7 | 338.1 | 333.9 | 0.012579 |
| wood | *A. alba* | 124 | 4 | 300.0 | 297.0 | 0.010101 |
| wood | *A. alba* | 124 | 4 | 483.0 | 477.2 | 0.012154 |
| wood | *A. alba* | 124 | 4 | 639.8 | 632.5 | 0.011542 |
| wood | *A. alba* | 124 | 4 | 763.7 | 754.4 | 0.012328 |
| wood | *A. alba* | 124 | 4 | 6944.2 | 6868.9 | 0.010962 |
| wood | *A. alba* | 124 | 4 | 1050.8 | 1038.7 | 0.011649 |
| wood | *A. alba* | 124 | 4 | 1434.1 | 1417.4 | 0.011782 |
| wood | *A. alba* | 124 | 4 | 2081.7 | 2058.9 | 0.011074 |
| wood | *A. alba* | 124 | 4 | 2875.6 | 2842.5 | 0.011645 |
| wood | *A. alba* | 124 | 4 | 2900.4 | 2866.3 | 0.011897 |
| wood | *A. alba* | 124 | 6 | 512.5 | 506.6 | 0.011646 |
| wood | *A. alba* | 125 | 4 | 2064.3 | 2041.6 | 0.011119 |
| wood | *A. alba* | 125 | 4 | 3754.2 | 3712.3 | 0.011287 |
| wood | *A. alba* | 125 | 4 | 540.3 | 534.1 | 0.011608 |
| wood | *A. alba* | 125 | 4 | 947.8 | 936.8 | 0.011742 |
| wood | *A. alba* | 125 | 8 | 102.9 | 101.5 | 0.013793 |
| wood | *L. decidua* | 25 | 2 | 203.3 | 201.2 | 0.010437 |
| wood | *L. decidua* | 25 | 3 | 3222.1 | 3191.8 | 0.009493 |
| wood | *L. decidua* | 25 | 3 | 1476.8 | 1462.9 | 0.009502 |
| wood | *L. decidua* | 25 | 4 | 2799.8 | 2776.6 | 0.008356 |
| wood | *L. decidua* | 25 | 5 | 1118.8 | 1107.9 | 0.009838 |
| wood | *L. decidua* | 25 | 7 | 2013.6 | 1994.6 | 0.009526 |
| wood | *L. decidua* | 25 | 8 | 1801.5 | 1785.4 | 0.009018 |
| wood | *L. decidua* | 36 | 1 | 451.8 | 447.2 | 0.010286 |
| wood | *L. decidua* | 36 | 2 | 761.1 | 753.3 | 0.010354 |
| wood | *L. decidua* | 36 | 2 | 2667.9 | 2644.9 | 0.008696 |
| wood | *L. decidua* | 36 | 3 | 492.2 | 486.9 | 0.010885 |
| wood | *L. decidua* | 36 | 3 | 2138.9 | 2119.2 | 0.009296 |
| wood | *L. decidua* | 36 | 4 | 507.0 | 502.9 | 0.008153 |
| wood | *L. decidua* | 36 | 4 | 1782.6 | 1766.6 | 0.009057 |
| wood | *L. decidua* | 36 | 6 | 1383.9 | 1370.5 | 0.009777 |
| wood | *L. decidua* | 36 | 7 | 1225.3 | 1213.7 | 0.009558 |
| wood | *L. decidua* | 36 | 8 | 1023.3 | 1013.8 | 0.009371 |
| wood | *L. decidua* | 44 | 1 | 1612.0 | 1596.0 | 0.010025 |
| wood | *L. decidua* | 44 | 2 | 889.4 | 880.0 | 0.010682 |
| wood | *L. decidua* | 44 | 4 | 1032.5 | 1022.7 | 0.009582 |
| wood | *L. decidua* | 44 | 5 | 888.1 | 879.8 | 0.009434 |
| wood | *L. decidua* | 44 | 6 | 701.8 | 695.2 | 0.009494 |
| wood | *L. decidua* | 54 | 1 | 3019.9 | 2991.5 | 0.009494 |
| wood | *L. decidua* | 54 | 3 | 705.0 | 698.0 | 0.010029 |
| wood | *L. decidua* | 54 | 7 | 710.9 | 704.4 | 0.009228 |
| wood | *L. decidua* | 59 | 2 | 794.1 | 786.9 | 0.009150 |
| wood | *L. decidua* | 59 | 3 | 800.3 | 792.5 | 0.009842 |
| wood | *L. decidua* | 59 | 5 | 563.8 | 558.5 | 0.009490 |
| wood | *L. decidua* | 59 | 6 | 621.6 | 615.4 | 0.010075 |
| wood | *L. decidua* | 59 | 6 | 3699.7 | 3666.0 | 0.009193 |
| wood | *L. decidua* | 59 | 8 | 423.1 | 419.0 | 0.009785 |
| wood | *L. decidua* | 63 | 2 | 428.5 | 424.1 | 0.010375 |
| wood | *L. decidua* | 63 | 7 | 1070.0 | 1059.9 | 0.009529 |
| wood | *L. decidua* | 63 | 8 | 1007.4 | 997.6 | 0.009824 |
| wood | *L. decidua* | 63 | 8 | 624.2 | 618.1 | 0.009869 |
| wood | *L. decidua* | 75 | 1 | 2017.1 | 1999.5 | 0.008802 |
| wood | *L. decidua* | 75 | 1 | 1244.7 | 1232.4 | 0.009981 |
| wood | *L. decidua* | 75 | 2 | 873.8 | 864.9 | 0.010290 |
| wood | *L. decidua* | 75 | 3 | 877.3 | 868.5 | 0.010132 |
| wood | *L. decidua* | 75 | 4 | 1260.3 | 1248.3 | 0.009613 |
| wood | *L. decidua* | 75 | 4 | 805.5 | 797.6 | 0.009905 |
| wood | *L. decidua* | 75 | 5 | 1236.7 | 1224.5 | 0.009963 |
| wood | *L. decidua* | 75 | 5 | 637.3 | 631.3 | 0.009504 |
| wood | *L. decidua* | 75 | 6 | 1145.5 | 1134.5 | 0.009696 |
| wood | *L. decidua* | 75 | 6 | 681.6 | 674.7 | 0.010227 |
| wood | *L. decidua* | 75 | 7 | 984.1 | 974.1 | 0.010266 |
| wood | *L. decidua* | 75 | 8 | 760.0 | 752.5 | 0.009967 |
| wood | *L. decidua* | 85 | 2 | 610.1 | 604.2 | 0.009765 |
| wood | *L. decidua* | 85 | 4 | 1576.1 | 1560.5 | 0.009997 |
| wood | *L. decidua* | 85 | 5 | 279.4 | 276.4 | 0.010854 |
| wood | *L. decidua* | 85 | 5 | 2344.5 | 2323.6 | 0.008995 |
| wood | *L. decidua* | 85 | 5 | 1437.8 | 1423.7 | 0.009904 |
| wood | *L. decidua* | 85 | 6 | 527.8 | 522.0 | 0.011111 |
| wood | *L. decidua* | 85 | 6 | 1499.9 | 1485.9 | 0.009422 |
| wood | *L. decidua* | 85 | 7 | 957.3 | 947.3 | 0.010556 |
| wood | *L. decidua* | 85 | 8 | 1442.9 | 1429.5 | 0.009374 |
| wood | *L. decidua* | 85 | 8 | 973.6 | 963.9 | 0.010063 |
| wood | *L. decidua* | 87 | 2 | 604.3 | 598.4 | 0.009860 |
| wood | *L. decidua* | 87 | 2 | 377.0 | 373.2 | 0.010182 |
| wood | *L. decidua* | 87 | 4 | 18.4 | 18.3 | 0.005464 |
| wood | *L. decidua* | 87 | 5 | 457.7 | 453.4 | 0.009484 |
| wood | *L. decidua* | 87 | 5 | 282.8 | 280.0 | 0.010000 |
| wood | *L. decidua* | 87 | 6 | 384.0 | 384.0 | 0.000000 |
| wood | *L. decidua* | 87 | 6 | 163.2 | 161.7 | 0.009276 |
| wood | *L. decidua* | 87 | 7 | 239.8 | 237.3 | 0.010535 |
| wood | *L. decidua* | 87 | 7 | 125.8 | 124.4 | 0.011254 |
| wood | *L. decidua* | 87 | 8 | 223.1 | 221.0 | 0.009502 |
| wood | *L. decidua* | 87 | 8 | 97.7 | 96.8 | 0.009298 |
| wood | *L. decidua* | 99 | 1 | 295.5 | 292.3 | 0.010948 |
| wood | *L. decidua* | 99 | 1 | 3958.3 | 3921.8 | 0.009307 |
| wood | *L. decidua* | 99 | 2 | 321.2 | 318.3 | 0.009111 |
| wood | *L. decidua* | 99 | 3 | 376.2 | 372.3 | 0.010475 |
| wood | *L. decidua* | 99 | 3 | 3007.6 | 2979.4 | 0.009465 |
| wood | *L. decidua* | 99 | 3 | 1788.1 | 1770.5 | 0.009941 |
| wood | *L. decidua* | 99 | 4 | 33.3 | 33.1 | 0.006042 |
| wood | *L. decidua* | 99 | 4 | 178.9 | 176.9 | 0.011306 |
| wood | *L. decidua* | 99 | 4 | 192.2 | 190.3 | 0.009984 |
| wood | *L. decidua* | 99 | 5 | 2089.5 | 2071.0 | 0.008933 |
| wood | *L. decidua* | 99 | 6 | 262.8 | 260.2 | 0.009992 |
| wood | *L. decidua* | 99 | 7 | 758.2 | 750.6 | 0.010125 |
| wood | *L. decidua* | 99 | 8 | 147.0 | 145.8 | 0.008230 |
| wood | *L. decidua* | 120 | 2 | 81.3 | 80.3 | 0.012453 |
| wood | *L. decidua* | 120 | 2 | 738.4 | 731.0 | 0.010123 |
| wood | *L. decidua* | 120 | 3 | 3313.1 | 3283.1 | 0.009138 |
| wood | *L. decidua* | 120 | 4 | 743.3 | 735.8 | 0.010193 |
| wood | *L. decidua* | 120 | 5 | 1305.5 | 1292.0 | 0.010449 |
| wood | *L. decidua* | 120 | 6 | 503.3 | 498.4 | 0.009831 |
| wood | *L. decidua* | 120 | 6 | 2077.7 | 2058.8 | 0.009180 |
| wood | *L. decidua* | 120 | 8 | 411.5 | 406.9 | 0.011305 |
| wood | *L. decidua* | 139 | 1 | 63.3 | 62.8 | 0.007962 |
| wood | *A. glutinosa* | 19 | 1 | 2062.0 | 2044.0 | 0.008806 |
| wood | *A. glutinosa* | 19 | 2 | 347.2 | 343.7 | 0.010183 |
| wood | *A. glutinosa* | 19 | 2 | 2955.4 | 2931.9 | 0.008015 |
| wood | *A. glutinosa* | 19 | 3 | 2065.1 | 2047.3 | 0.008694 |
| wood | *A. glutinosa* | 19 | 5 | 1811.8 | 1797.9 | 0.007731 |
| wood | *A. glutinosa* | 19 | 5 | 1026.1 | 1018.0 | 0.007957 |
| wood | *A. glutinosa* | 19 | 8 | 730.2 | 724.1 | 0.008424 |
| wood | *A. glutinosa* | 20 | 1 | 1314.3 | 1303.4 | 0.008363 |
| wood | *A. glutinosa* | 20 | 2 | 1179.4 | 1170.2 | 0.007862 |
| wood | *A. glutinosa* | 20 | 3 | 1395.5 | 1384.1 | 0.008236 |
| wood | *A. glutinosa* | 20 | 7 | 769.8 | 763.8 | 0.007855 |
| wood | *A. glutinosa* | 20 | 8 | 291.9 | 289.8 | 0.007246 |
| wood | *A. glutinosa* | 31 | 1 | 3139.0 | 3115.3 | 0.007608 |
| wood | *A. glutinosa* | 31 | 1 | 2218.8 | 2202.7 | 0.007309 |
| wood | *A. glutinosa* | 31 | 2 | 2105.4 | 2089.6 | 0.007561 |
| wood | *A. glutinosa* | 31 | 2 | 1297.2 | 1286.5 | 0.008317 |
| wood | *A. glutinosa* | 31 | 3 | 1783.2 | 1768.6 | 0.008255 |
| wood | *A. glutinosa* | 31 | 3 | 1052.7 | 1044.0 | 0.008333 |
| wood | *A. glutinosa* | 31 | 4 | 839.8 | 831.7 | 0.009739 |
| wood | *A. glutinosa* | 31 | 6 | 990.7 | 982.7 | 0.008141 |
| wood | *A. glutinosa* | 31 | 7 | 873.0 | 866.4 | 0.007618 |
| wood | *A. glutinosa* | 50 | 3 | 600.0 | 595.1 | 0.008234 |
| wood | *A. glutinosa* | 53 | 1 | 1693.6 | 1681.6 | 0.007136 |
| wood | *A. glutinosa* | 53 | 1 | 1144.4 | 1135.8 | 0.007572 |
| wood | *A. glutinosa* | 53 | 2 | 1240.5 | 1231.3 | 0.007472 |
| wood | *A. glutinosa* | 53 | 3 | 1142.6 | 1134.2 | 0.007406 |
| wood | *A. glutinosa* | 53 | 4 | 869.4 | 863.0 | 0.007416 |
| wood | *A. glutinosa* | 53 | 6 | 591.8 | 587.1 | 0.008005 |
| wood | *A. glutinosa* | 55 | 1 | 1607.3 | 1593.9 | 0.008407 |
| wood | *A. glutinosa* | 55 | 2 | 2132.9 | 2116.9 | 0.007558 |
| wood | *A. glutinosa* | 55 | 3 | 1551.0 | 1539.5 | 0.007470 |
| wood | *A. glutinosa* | 55 | 3 | 804.3 | 798.0 | 0.007895 |
| wood | *A. glutinosa* | 55 | 4 | 1541.3 | 1529.2 | 0.007913 |
| wood | *A. glutinosa* | 55 | 4 | 747.3 | 742.0 | 0.007143 |
| wood | *A. glutinosa* | 55 | 8 | 581.6 | 577.3 | 0.007448 |
| wood | *A. glutinosa* | 66 | 1 | 921.1 | 914.2 | 0.007548 |
| wood | *A. glutinosa* | 66 | 1 | 590.0 | 586.0 | 0.006826 |
| wood | *A. glutinosa* | 66 | 2 | 770.9 | 764.5 | 0.008371 |
| wood | *A. glutinosa* | 66 | 3 | 644.0 | 639.1 | 0.007667 |
| wood | *A. glutinosa* | 66 | 4 | 471.5 | 467.7 | 0.008125 |
| wood | *A. glutinosa* | 102 | 1 | 169.2 | 167.7 | 0.008945 |
| wood | *A. glutinosa* | 102 | 1 | 1181.7 | 1172.4 | 0.007932 |
| wood | *A. glutinosa* | 102 | 2 | 111.2 | 110.6 | 0.005425 |
| wood | *A. glutinosa* | 102 | 2 | 762.9 | 756.5 | 0.008460 |
| wood | *A. glutinosa* | 102 | 3 | 1343.8 | 1333.2 | 0.007951 |
| wood | *A. glutinosa* | 102 | 3 | 103.4 | 102.8 | 0.005837 |
| wood | *A. glutinosa* | 102 | 4 | 9.6 | 9.5 | 0.010526 |
| wood | *A. glutinosa* | 102 | 4 | 41.9 | 41.6 | 0.007212 |
| wood | *A. glutinosa* | 102 | 4 | 177.3 | 176.3 | 0.005672 |
| wood | *A. glutinosa* | 102 | 4 | 335.8 | 333.2 | 0.007803 |
| wood | *A. glutinosa* | 102 | 4 | 425.1 | 421.7 | 0.008063 |
| wood | *A. glutinosa* | 102 | 4 | 524.1 | 520.1 | 0.007691 |
| wood | *A. glutinosa* | 102 | 4 | 1105.9 | 1097.6 | 0.007562 |
| wood | *A. glutinosa* | 102 | 4 | 1300.2 | 1289.7 | 0.008141 |
| wood | *A. glutinosa* | 102 | 4 | 593.3 | 588.7 | 0.007814 |
| wood | *A. glutinosa* | 102 | 4 | 670.1 | 665.1 | 0.007518 |
| wood | *A. glutinosa* | 102 | 4 | 713.0 | 707.9 | 0.007204 |
| wood | *A. glutinosa* | 102 | 4 | 805.5 | 799.5 | 0.007505 |
| wood | *A. glutinosa* | 102 | 4 | 829.7 | 822.5 | 0.008754 |
| wood | *A. glutinosa* | 102 | 4 | 834.9 | 828.1 | 0.008212 |
| wood | *A. glutinosa* | 102 | 4 | 944.2 | 937.1 | 0.007577 |
| wood | *A. glutinosa* | 102 | 4 | 989.8 | 982.3 | 0.007635 |
| wood | *A. glutinosa* | 102 | 5 | 1195.4 | 1186.1 | 0.007841 |
| wood | *A. glutinosa* | 102 | 5 | 698.7 | 693.7 | 0.007208 |
| wood | *A. glutinosa* | 102 | 6 | 825.4 | 819.1 | 0.007691 |
| wood | *A. glutinosa* | 102 | 6 | 479.0 | 475.4 | 0.007573 |
| wood | *A. glutinosa* | 102 | 7 | 88.6 | 87.9 | 0.007964 |
| wood | *A. glutinosa* | 102 | 7 | 590.7 | 585.9 | 0.008193 |
| wood | *A. glutinosa* | 102 | 7 | 331.4 | 329.0 | 0.007295 |
| wood | *A. glutinosa* | 135 | 1 | 347.4 | 344.4 | 0.008711 |
| wood | *A. glutinosa* | 135 | 1 | 166.6 | 165.0 | 0.009697 |
| wood | *A. glutinosa* | 135 | 2 | 185.4 | 183.8 | 0.008705 |
| wood | *A. glutinosa* | 135 | 3 | 40.6 | 40.4 | 0.004950 |
| wood | *A. glutinosa* | 135 | 3 | 72.5 | 72.2 | 0.004155 |
| wood | *A. glutinosa* | 135 | 3 | 106.5 | 105.7 | 0.007569 |
| wood | *A. glutinosa* | 135 | 3 | 183.2 | 181.7 | 0.008255 |
| wood | *A. glutinosa* | 135 | 3 | 192.5 | 190.9 | 0.008381 |
| wood | *A. glutinosa* | 135 | 3 | 197.2 | 195.8 | 0.007150 |
| wood | *A. glutinosa* | 135 | 3 | 356.1 | 353.5 | 0.007355 |
| wood | *A. glutinosa* | 135 | 3 | 83.1 | 82.5 | 0.007273 |
| wood | *A. glutinosa* | 135 | 3 | 133.5 | 132.6 | 0.006787 |
| wood | *A. glutinosa* | 135 | 3 | 144.4 | 143.5 | 0.006272 |
| wood | *A. glutinosa* | 135 | 3 | 169.8 | 168.6 | 0.007117 |
| wood | *A. glutinosa* | 135 | 4 | 154.7 | 153.3 | 0.009132 |
| wood | *A. glutinosa* | 135 | 4 | 87.9 | 87.2 | 0.008028 |
| wood | *A. glutinosa* | 135 | 5 | 105.1 | 104.7 | 0.003820 |
| wood | *A. glutinosa* | 135 | 5 | 74.9 | 74.6 | 0.004021 |
| wood | *A. glutinosa* | 135 | 6 | 96.7 | 96.0 | 0.007292 |
| wood | *A. glutinosa* | 135 | 7 | 81.6 | 80.9 | 0.008653 |
| wood | *A. glutinosa* | 135 | 7 | 42.5 | 42.5 | 0.000000 |
| wood | *P. sylvestris* | 22 | 5 | 428.2 | 424.1 | 0.009668 |
| wood | *P. sylvestris* | 22 | 6 | 1745.4 | 1731.0 | 0.008319 |
| wood | *P. sylvestris* | 22 | 7 | 1664.9 | 1650.3 | 0.008847 |
| wood | *P. sylvestris* | 22 | 8 | 959.6 | 950.7 | 0.009362 |
| wood | *P. sylvestris* | 23 | 3 | 299.0 | 296.2 | 0.009453 |
| wood | *P. sylvestris* | 23 | 6 | 400.1 | 396.6 | 0.008825 |
| wood | *P. sylvestris* | 26 | 2 | 3199.7 | 3166.4 | 0.010517 |
| wood | *P. sylvestris* | 26 | 4 | 2667.2 | 2639.7 | 0.010418 |
| wood | *P. sylvestris* | 26 | 6 | 1930.4 | 1911.2 | 0.010046 |
| wood | *P. sylvestris* | 26 | 7 | 1217.9 | 1206.8 | 0.009198 |
| wood | *P. sylvestris* | 26 | 8 | 2252.4 | 2230.0 | 0.010045 |
| wood | *P. sylvestris* | 33 | 1 | 261.0 | 258.4 | 0.010062 |
| wood | *P. sylvestris* | 33 | 1 | 2014.2 | 1995.1 | 0.009573 |
| wood | *P. sylvestris* | 33 | 2 | 1976.2 | 1958.0 | 0.009295 |
| wood | *P. sylvestris* | 33 | 2 | 1027.6 | 1018.4 | 0.009034 |
| wood | *P. sylvestris* | 33 | 3 | 1623.3 | 1608.2 | 0.009389 |
| wood | *P. sylvestris* | 33 | 4 | 1278.4 | 1267.3 | 0.008759 |
| wood | *P. sylvestris* | 33 | 4 | 626.3 | 620.7 | 0.009022 |
| wood | *P. sylvestris* | 42 | 1 | 2692.4 | 2667.0 | 0.009524 |
| wood | *P. sylvestris* | 42 | 3 | 1738.8 | 1721.6 | 0.009991 |
| wood | *P. sylvestris* | 42 | 4 | 1710.3 | 1696.0 | 0.008432 |
| wood | *P. sylvestris* | 42 | 4 | 965.1 | 956.6 | 0.008886 |
| wood | *P. sylvestris* | 42 | 5 | 1278.5 | 1267.7 | 0.008519 |
| wood | *P. sylvestris* | 42 | 7 | 850.6 | 842.9 | 0.009135 |
| wood | *P. sylvestris* | 47 | 3 | 453.2 | 449.0 | 0.009354 |
| wood | *P. sylvestris* | 47 | 6 | 1419.5 | 1406.5 | 0.009243 |
| wood | *P. sylvestris* | 47 | 7 | 1664.5 | 1649.7 | 0.008971 |
| wood | *P. sylvestris* | 47 | 7 | 765.6 | 759.4 | 0.008164 |
| wood | *P. sylvestris* | 47 | 8 | 1675.8 | 1661.1 | 0.008850 |
| wood | *P. sylvestris* | 52 | 1 | 688.8 | 681.8 | 0.010267 |
| wood | *P. sylvestris* | 52 | 6 | 2546.8 | 2523.8 | 0.009113 |
| wood | *P. sylvestris* | 52 | 7 | 232.6 | 230.0 | 0.011304 |
| wood | *P. sylvestris* | 52 | 7 | 1153.9 | 1142.7 | 0.009801 |
| wood | *P. sylvestris* | 56 | 2 | 2104.0 | 2083.4 | 0.009888 |
| wood | *P. sylvestris* | 56 | 3 | 1226.0 | 1214.9 | 0.009137 |
| wood | *P. sylvestris* | 56 | 4 | 234.0 | 231.6 | 0.010363 |
| wood | *P. sylvestris* | 56 | 7 | 186.4 | 184.6 | 0.009751 |
| wood | *P. sylvestris* | 56 | 7 | 1230.1 | 1219.1 | 0.009023 |
| wood | *P. sylvestris* | 56 | 8 | 1200.0 | 1189.1 | 0.009167 |
| wood | *P. sylvestris* | 57 | 1 | 1134.2 | 1123.0 | 0.009973 |
| wood | *P. sylvestris* | 57 | 3 | 611.8 | 604.1 | 0.012746 |
| wood | *P. sylvestris* | 57 | 3 | 2932.8 | 2906.7 | 0.008979 |
| wood | *P. sylvestris* | 57 | 6 | 759.7 | 753.2 | 0.008630 |
| wood | *P. sylvestris* | 57 | 6 | 2451.6 | 2428.0 | 0.009720 |
| wood | *P. sylvestris* | 57 | 8 | 1347.6 | 1336.7 | 0.008154 |
| wood | *P. sylvestris* | 62 | 3 | 3231.4 | 3203.4 | 0.008741 |
| wood | *P. sylvestris* | 62 | 4 | 1467.5 | 1453.7 | 0.009493 |
| wood | *P. sylvestris* | 62 | 5 | 1606.6 | 1590.8 | 0.009932 |
| wood | *P. sylvestris* | 62 | 6 | 1978.4 | 1960.4 | 0.009182 |
| wood | *P. sylvestris* | 67 | 2 | 2144.1 | 2123.7 | 0.009606 |
| wood | *P. sylvestris* | 67 | 2 | 1121.7 | 1112.2 | 0.008542 |
| wood | *P. sylvestris* | 67 | 3 | 1595.0 | 1579.6 | 0.009749 |
| wood | *P. sylvestris* | 67 | 3 | 982.4 | 972.1 | 0.010596 |
| wood | *P. sylvestris* | 67 | 5 | 731.8 | 725.2 | 0.009101 |
| wood | *P. sylvestris* | 67 | 6 | 665.6 | 659.1 | 0.009862 |
| wood | *P. sylvestris* | 74 | 5 | 232.1 | 229.7 | 0.010448 |
| wood | *P. sylvestris* | 76 | 5 | 1013.3 | 1003.8 | 0.009464 |
| wood | *P. sylvestris* | 80 | 2 | 2284.1 | 2262.5 | 0.009547 |
| wood | *P. sylvestris* | 80 | 4 | 970.1 | 962.2 | 0.008210 |
| wood | *P. sylvestris* | 80 | 8 | 1235.1 | 1224.2 | 0.008904 |
| wood | *P. sylvestris* | 88 | 1 | 1131.7 | 1121.4 | 0.009185 |
| wood | *P. sylvestris* | 88 | 1 | 691.4 | 684.4 | 0.010228 |
| wood | *P. sylvestris* | 88 | 1 | 92.0 | 91.0 | 0.010989 |
| wood | *P. sylvestris* | 88 | 2 | 162.0 | 160.0 | 0.012500 |
| wood | *P. sylvestris* | 88 | 4 | 555.2 | 550.4 | 0.008721 |
| wood | *P. sylvestris* | 88 | 5 | 683.2 | 677.4 | 0.008562 |
| wood | *P. sylvestris* | 101 | 1 | 965.3 | 955.4 | 0.010362 |
| wood | *P. sylvestris* | 101 | 1 | 3793.0 | 3758.4 | 0.009206 |
| wood | *P. sylvestris* | 101 | 2 | 638.1 | 632.1 | 0.009492 |
| wood | *P. sylvestris* | 101 | 2 | 2905.0 | 2877.5 | 0.009557 |
| wood | *P. sylvestris* | 101 | 4 | 1671.2 | 1655.3 | 0.009606 |
| wood | *P. sylvestris* | 101 | 7 | 1101.0 | 1090.9 | 0.009258 |
| wood | *P. sylvestris* | 128 | 1 | 162.5 | 160.8 | 0.010572 |
| wood | *P. sylvestris* | 128 | 2 | 115.3 | 114.2 | 0.009632 |
| wood | *P. sylvestris* | 128 | 4 | 12.5 | 12.3 | 0.016260 |
| wood | *P. sylvestris* | 128 | 4 | 92.9 | 91.6 | 0.014192 |
| wood | *P. sylvestris* | 128 | 6 | 4.3 | 4.3 | 0.000000 |
| wood | *P. sylvestris* | 128 | 8 | 9.3 | 9.2 | 0.010870 |
| wood | *P. sylvestris* | 136 | 1 | 5.0 | 4.9 | 0.020408 |
| wood | *P. sylvestris* | 136 | 4 | 3.7 | 3.7 | 0.000000 |
| wood | *P. sylvestris* | 138 | 2 | 10.4 | 10.2 | 0.019608 |
| wood | *P. sylvestris* | 138 | 3 | 16.6 | 16.4 | 0.012195 |
| wood | *P. sylvestris* | 138 | 3 | 275.7 | 273.1 | 0.009520 |
| wood | *P. sylvestris* | 138 | 4 | 88.6 | 87.3 | 0.014891 |
| wood | *P. sylvestris* | 138 | 5 | 11.0 | 11.0 | 0.000000 |
| wood | *P. sylvestris* | 138 | 5 | 12.6 | 12.3 | 0.024390 |
| wood | *P. sylvestris* | 138 | 6 | 9.6 | 9.5 | 0.010526 |
| wood | *P. sylvestris* | 138 | 6 | 59.7 | 58.9 | 0.013582 |
| wood | *P. sylvestris* | 138 | 7 | 47.2 | 46.6 | 0.012876 |
| wood | *P. sylvestris* | 138 | 8 | 37.4 | 37.0 | 0.010811 |
| wood | *P. abies* | 16 | 1 | 2471.1 | 2445.6 | 0.010427 |
| wood | *P. abies* | 28 | 2 | 1232.2 | 1220.1 | 0.009917 |
| wood | *P. abies* | 28 | 5 | 84.6 | 83.5 | 0.013174 |
| wood | *P. abies* | 46 | 5 | 123.4 | 122.1 | 0.010647 |
| wood | *P. abies* | 46 | 5 | 1218.3 | 1205.0 | 0.011037 |
| wood | *P. abies* | 46 | 5 | 645.9 | 639.7 | 0.009692 |
| wood | *P. abies* | 51 | 5 | 1608.2 | 1590.3 | 0.011256 |
| wood | *P. abies* | 69 | 4 | 942.2 | 933.2 | 0.009644 |
| wood | *P. abies* | 69 | 7 | 452.0 | 447.4 | 0.010282 |
| wood | *P. abies* | 71 | 2 | 2240.2 | 2216.7 | 0.010601 |
| wood | *P. abies* | 71 | 7 | 1969.8 | 1950.8 | 0.009740 |
| wood | *P. abies* | 73 | 6 | 3493.4 | 3455.7 | 0.010910 |
| wood | *P. abies* | 78 | 1 | 232.0 | 229.7 | 0.010013 |
| wood | *P. abies* | 78 | 3 | 222.6 | 220.4 | 0.009982 |
| wood | *P. abies* | 81 | 4 | 35.8 | 35.4 | 0.011299 |
| wood | *P. abies* | 81 | 8 | 12.3 | 12.2 | 0.008197 |
| wood | *P. abies* | 98 | 1 | 1730.3 | 1711.2 | 0.011162 |
| wood | *P. abies* | 98 | 2 | 1341.6 | 1329.0 | 0.009481 |
| wood | *P. abies* | 98 | 3 | 3596.2 | 3562.8 | 0.009375 |
| wood | *P. abies* | 98 | 6 | 1166.9 | 1153.6 | 0.011529 |
| wood | *P. abies* | 103 | 1 | 7.0 | 7.0 | 0.000000 |
| wood | *P. abies* | 103 | 1 | 40.0 | 39.4 | 0.015228 |
| wood | *P. abies* | 103 | 1 | 124.2 | 122.9 | 0.010578 |
| wood | *P. abies* | 103 | 1 | 253.6 | 251.1 | 0.009956 |
| wood | *P. abies* | 103 | 1 | 271.8 | 269.1 | 0.010033 |
| wood | *P. abies* | 103 | 4 | 60.0 | 59.4 | 0.010101 |
| wood | *P. abies* | 103 | 6 | 2.1 | 2.1 | 0.000000 |
| wood | *P. abies* | 103 | 8 | 5.5 | 5.4 | 0.018519 |
| wood | *P. abies* | 106 | 1 | 17.5 | 17.2 | 0.017442 |
| wood | *P. abies* | 106 | 1 | 38.2 | 37.7 | 0.013263 |
| wood | *P. abies* | 106 | 1 | 48.5 | 48.0 | 0.010417 |
| wood | *P. abies* | 106 | 3 | 42.6 | 42.2 | 0.009479 |
| wood | *P. abies* | 106 | 3 | 11.3 | 11.1 | 0.018018 |
| wood | *P. abies* | 106 | 7 | 8.1 | 8.0 | 0.012500 |
| wood | *P. abies* | 107 | 2 | 2586.2 | 2563.5 | 0.008855 |
| wood | *P. abies* | 107 | 5 | 861.7 | 853.5 | 0.009607 |
| wood | *P. abies* | 110 | 1 | 77.3 | 76.4 | 0.011780 |
| wood | *P. abies* | 110 | 3 | 15.6 | 15.4 | 0.012987 |
| wood | *P. abies* | 110 | 3 | 576.7 | 570.9 | 0.010159 |
| wood | *P. abies* | 110 | 3 | 698.5 | 691.0 | 0.010854 |
| wood | *P. abies* | 110 | 3 | 821.9 | 814.3 | 0.009333 |
| wood | *P. abies* | 110 | 3 | 588.1 | 582.3 | 0.009961 |
| wood | *P. abies* | 110 | 4 | 302.5 | 299.2 | 0.011029 |
| wood | *P. abies* | 110 | 5 | 479.8 | 475.1 | 0.009893 |
| wood | *P. abies* | 114 | 4 | 2839.7 | 2812.6 | 0.009635 |
| wood | *P. abies* | 114 | 4 | 2878.8 | 2852.2 | 0.009326 |
| wood | *P. abies* | 114 | 4 | 965.8 | 956.3 | 0.009934 |
| wood | *P. abies* | 114 | 4 | 1305.0 | 1292.2 | 0.009906 |
| wood | *P. abies* | 114 | 4 | 2352.8 | 2331.1 | 0.009309 |
| wood | *P. abies* | 117 | 4 | 18.6 | 18.2 | 0.021978 |
| wood | *P. abies* | 117 | 4 | 2419.1 | 2395.0 | 0.010063 |
| wood | *P. abies* | 117 | 6 | 89.8 | 88.6 | 0.013544 |
| wood | *P. abies* | 118 | 3 | 1918.0 | 1899.2 | 0.009899 |
| wood | *P. abies* | 118 | 3 | 3133.8 | 3103.3 | 0.009828 |
| wood | *P. abies* | 118 | 3 | 3617.0 | 3579.4 | 0.010505 |
| wood | *P. abies* | 118 | 3 | 797.1 | 789.2 | 0.010010 |
| wood | *P. abies* | 118 | 3 | 1205.6 | 1190.9 | 0.012344 |
| wood | *P. abies* | 118 | 5 | 134.2 | 132.9 | 0.009782 |
| wood | *P. abies* | 118 | 6 | 1021.4 | 1011.4 | 0.009887 |
| wood | *P. abies* | 123 | 2 | 13.6 | 13.4 | 0.014925 |
| wood | *P. abies* | 123 | 2 | 102.9 | 101.6 | 0.012795 |
| wood | *P. abies* | 123 | 2 | 471.4 | 466.7 | 0.010071 |
| wood | *P. abies* | 123 | 2 | 488.0 | 483.6 | 0.009098 |
| wood | *P. abies* | 123 | 2 | 548.0 | 542.7 | 0.009766 |
| wood | *P. abies* | 123 | 7 | 104.5 | 103.5 | 0.009662 |
| wood | *P. abies* | 126 | 1 | 237.1 | 234.6 | 0.010656 |
| wood | *P. abies* | 126 | 3 | 2073.2 | 2051.4 | 0.010627 |
| wood | *P. abies* | 126 | 3 | 765.8 | 758.5 | 0.009624 |
| wood | *P. abies* | 126 | 4 | 49.2 | 48.5 | 0.014433 |
| wood | *P. abies* | 126 | 4 | 339.0 | 335.5 | 0.010432 |
| wood | *P. abies* | 126 | 4 | 1522.9 | 1505.3 | 0.011692 |
| wood | *P. abies* | 126 | 4 | 1596.9 | 1579.2 | 0.011208 |
| wood | *P. abies* | 126 | 4 | 1667.0 | 1651.0 | 0.009691 |
| wood | *P. abies* | 126 | 4 | 488.4 | 482.7 | 0.011809 |
| wood | *P. abies* | 126 | 4 | 1261.0 | 1248.8 | 0.009769 |
| wood | *P. abies* | 126 | 5 | 92.5 | 91.5 | 0.010929 |
| wood | *P. abies* | 126 | 7 | 84.1 | 82.9 | 0.014475 |
| wood | *P. abies* | 126 | 8 | 90.0 | 88.9 | 0.012373 |
| wood | *P. abies* | 140 | 1 | 551.4 | 545.9 | 0.010075 |
| wood | *P. abies* | 140 | 2 | 359.2 | 355.9 | 0.009272 |
| wood | *P. abies* | 140 | 2 | 423.9 | 419.6 | 0.010248 |
| wood | *P. abies* | 140 | 2 | 766.6 | 759.5 | 0.009348 |
| wood | *P. abies* | 140 | 2 | 983.4 | 973.1 | 0.010585 |
| wood | *P. abies* | 140 | 2 | 513.9 | 508.7 | 0.010222 |
| wood | *P. abies* | 140 | 2 | 657.0 | 651.5 | 0.008442 |
| wood | *P. abies* | 140 | 2 | 671.7 | 664.4 | 0.010987 |
| wood | *P. abies* | 140 | 3 | 61.3 | 60.6 | 0.011551 |
| wood | *P. abies* | 140 | 5 | 42.9 | 42.4 | 0.011792 |
| wood | *P. abies* | 142 | 5 | 7.4 | 7.3 | 0.013699 |
| wood | *P. abies* | 169 | 5 | 4753.6 | 4709.9 | 0.009278 |

**Table S2.** Overview of the study plot characteristics

| **Plot number** | **Forest Inspectorate** | **Forest compartment** | **N [°]** | **E [°]** | **Tree species** | **Tree stand age**  **[years]** | **Basal area**  **[m^2^ ha^-1^]** | **Tree stand volume**  **[m^3^ ha^-1^]** | **Tree stand density**  **[ind. ha^-1^]** | **Mean DBH [cm]** | **Basal area**  **weighted**  **mean height [m]** |
| --- | --- | --- | --- | --- | --- | --- | --- | --- | --- | --- | --- |
| 45 | Trzebież | 16g | 53.7040 | 14.3353 | *B. pendula* | 47 | 20.4 | 188.2 | 617.1 | 20.0 | 21.0 |
| 30 | Dębno | 438k | 52.7101 | 14.4776 | *B. pendula* | 32 | 16.1 | 152.7 | 704.8 | 16.7 | 18.5 |
| 134 | Kup | 37c | 50.9471 | 17.8219 | *B. pendula* | 41 | 16.6 | 187.6 | 340.0 | 24.5 | 24.5 |
| 58 | Sławno | 368d | 54.4029 | 16.7524 | *B. pendula* | 53 | 23.9 | 316.5 | 590.7 | 22.2 | 24.3 |
| 86 | Golub-Dobrzyń | 293g | 53.0923 | 19.0984 | *B. pendula* | 81 | 13.3 | 161.5 | 98.7 | 40.9 | 28.5 |
| 89 | Kłobuck | 198d | 50.9251 | 19.0857 | *B. pendula* | 19 | 16.9 | 110.8 | 3159.1 | 7.9 | 11.6 |
| 72 | Warcino | 132f | 54.2741 | 16.9395 | *B. pendula* | 24 | 17.0 | 149.6 | 1188.9 | 13.1 | 15.8 |
| 171 | Szubin | 62l | 53.1224 | 17.4300 | *B. pendula* | 11 | 7.8 | 34.2 | 4708.3 | 5.4 | 6.6 |
| 39 | Wolsztyn | 130d | 52.1413 | 16.1574 | *B. pendula* | 6 | 1.1 | 5.0 | 4976.1 | 2.3 | 3.1 |
| 132 | Strzelce Opolskie | 20b | 50.4436 | 18.2412 | *F. sylvatica* | 86 | 34.6 | 527.4 | 217.4 | 43.6 | 34.7 |
| 100 | Bardo Śląskie | 221c | 50.4170 | 16.8433 | *F. sylvatica* | 93 | 41.1 | 650.8 | 398.4 | 35.5 | 29.4 |
| 104 | Sucha | 307a | 49.6751 | 19.4771 | *F. sylvatica* | 72 | 34.6 | 324.4 | 492.0 | 29.1 | 27.7 |
| 108 | Andrychów | 73f | 49.9043 | 19.2423 | *F. sylvatica* | 54 | 24.3 | 304.2 | 552.4 | 22.7 | 25.5 |
| 144 | Leśny Dwór | 35g | 54.4299 | 17.2145 | *F. sylvatica* | 46 | 32.3 | 361.2 | 750.0 | 22.2 | 23.8 |
| 170 | Sławno | 29d | 54.3852 | 16.7894 | *F. sylvatica* | 68 | 35.0 | 444.3 | 501.1 | 28.9 | 27.0 |
| 93 | Świdnica | 241g | 50.6606 | 16.5520 | *F. sylvatica* | 66 | 36.5 | 418.6 | 460.0 | 30.7 | 26.4 |
| 131 | Olkusz | 27c | 50.3049 | 19.5953 | *F. sylvatica* | 43 | 37.3 | 474.8 | 505.5 | 29.4 | 27.8 |
| 119 | Ustroń | 152a | 49.7370 | 18.8552 | *F. sylvatica* | 110 | 36.7 | 545.6 | 275.3 | 40.1 | 31.4 |
| 37 | Nowogard | 143i | 53.6447 | 15.0965 | *F. sylvatica* | 37 | 34.2 | 348.7 | 3235.3 | 10.9 | 18.3 |
| 18 | Międzychód | 97n | 52.5966 | 15.7047 | *F. sylvatica* | 82 | 28.9 | 372.9 | 507.3 | 25.6 | 25.6 |
| 35 | Gryfino | 164b | 53.3411 | 14.6783 | *F. sylvatica* | 117 | 38.2 | 753.5 | 205.4 | 48.2 | 42.3 |
| 111 | Jeleśnia | 91a | 49.5538 | 19.3631 | *F. sylvatica* | 34 | 25.2 | 143.9 | 1490.2 | 13.2 | 13.2 |
| 65 | Lipka | 75a | 53.5006 | 17.2043 | *F. sylvatica* | 75 | 35.0 | 565.6 | 340.0 | 35.4 | 33.2 |
| 116 | Wisła | 86h | 49.6266 | 18.9197 | *F. sylvatica* | 111 | 30.4 | 514.6 | 245.2 | 39.1 | 33.0 |
| 41 | Świebodzin | 16a | 52.3853 | 15.3251 | *F. sylvatica* | 99 | 31.8 | 590.2 | 166.7 | 48.6 | 38.2 |
| 130 | Sulęcin | 217d | 52.4197 | 15.3192 | *F. sylvatica* | 28 | 14.6 | 95.8 | 3933.3 | 6.6 | 10.3 |
| 129 | Bielsko | 32i | 49.8705 | 19.1100 | *F. sylvatica* | 8 | 0.6 | 3.6 | 6993.5 | 1.8 | 2.1 |
| 43 | Świebodzin | 326c | 52.2757 | 15.5817 | *Q. robur* | 64 | 25.4 | 248.8 | 705.9 | 20.7 | 19.0 |
| 29 | Dębno | 64k | 52.7958 | 14.6552 | *Q. robur* | 89 | 32.0 | 507.7 | 301.3 | 35.9 | 30.7 |
| 64 | Złotów | 394d | 53.2905 | 17.2527 | *Q. robur* | 49 | 17.7 | 204.0 | 301.7 | 26.8 | 21.6 |
| 34 | Gryfino | 440i | 53.1169 | 14.5314 | *Q. robur* | 42 | 24.8 | 246.2 | 875.5 | 18.4 | 18.6 |
| 84 | Golub-Dobrzyń | 186c | 53.1411 | 19.1355 | *Q. robur* | 40 | 16.8 | 135.2 | 487.1 | 20.0 | 16.2 |
| 79 | Durowo | 169i | 52.7987 | 17.1433 | *Q. robur* | 61 | 21.6 | 240.1 | 520.0 | 22.3 | 20.9 |
| 49 | Nowogard | 271b | 53.5613 | 15.1797 | *Q. robur* | 55 | 15.1 | 168.5 | 282.5 | 25.4 | 21.5 |
| 24 | Strzelce Krajeńskie | 86b | 52.9382 | 15.6493 | *Q. robur* | 29 | 20.9 | 138.6 | 1734.7 | 11.8 | 11.7 |
| 127 | Wolsztyn | 1d | 52.2403 | 16.0762 | *Q. robur* | 19 | 11.5 | 80.9 | 1311.1 | 10.2 | 11.7 |
| 68 | Krucz | 27f | 52.8618 | 16.5430 | *Q. robur* | 79 | 27.9 | 448.8 | 330.3 | 32.3 | 29.4 |
| 133 | Strzelce Opolskie | 32i | 50.4263 | 18.2943 | *Q. robur* | 11 | 4.7 | 17.4 | 4678.6 | 4.0 | 5.0 |
| 141 | Lwówek Śląski | 1i | 51.1996 | 15.6067 | *Q. robur* | 10 | 4.3 | 19.1 | 5066.7 | 4.2 | 4.3 |
| 32 | Dąbrowa | 283g | 53.5443 | 18.5806 | *Q. robur* | 16 | 20.8 | 118.0 | 5830.0 | 6.4 | 9.1 |
| 61 | Wałcz | 593h | 53.2661 | 16.4058 | *Q. robur* | 112 | 24.9 | 389.2 | 152.2 | 44.8 | 32.0 |
| 143 | Leśny Dwór | 594g | 54.4302 | 17.0396 | *Q. robur* | 121 | 39.7 | 644.7 | 247.7 | 44.7 | 31.7 |
| 83 | Gniewkowo | 520b | 52.7984 | 18.7352 | *Q. robur* | 89 | 24.0 | 343.1 | 232.8 | 35.4 | 28.3 |
| 77 | Lipinki | 142h | 51.6250 | 14.9196 | *Q. robur* | 132 | 38.3 | 572.6 | 204.6 | 46.9 | 29.8 |
| 137 | Miradz | 59g | 52.5951 | 18.1458 | *Q. robur* | 113 | 25.9 | 364.4 | 181.7 | 41.9 | 29.7 |
| 48 | Goleniów | 342f | 53.6657 | 14.8091 | *Q. robur* | 120 | 25.3 | 388.6 | 133.3 | 48.4 | 30.8 |
| 21 | Bolewice | 18g | 52.5320 | 15.9714 | *Q. robur* | 141 | 30.5 | 519.8 | 122.1 | 55.4 | 31.1 |
| 115 | Ujsoły | 50b | 49.5263 | 19.1669 | *A. alba* | 40 | 41.8 | 321.8 | 1851.9 | 15.9 | 14.1 |
| 113 | Jeleśnia | 247a | 49.5763 | 19.2259 | *A. alba* | 78 | 41.4 | 565.5 | 581.0 | 29.1 | 25.5 |
| 121 | Olkusz | 27c | 50.3051 | 19.5958 | *A. alba* | 41 | 43.8 | 557.4 | 1110.2 | 21.3 | 22.9 |
| 109 | Andrychów | 47b | 49.9213 | 19.6542 | *A. alba* | 57 | 30.5 | 351.4 | 573.8 | 24.6 | 23.1 |
| 122 | Andrychów | 150a | 49.8572 | 19.6546 | *A. alba* | 107 | 35.5 | 582.6 | 235.3 | 42.0 | 33.0 |
| 124 | Olkusz | 105a | 50.4985 | 19.6311 | *A. alba* | 115 | 37.0 | 560.8 | 211.8 | 46.5 | 31.5 |
| 125 | Węgierska Górka | 3a | 49.6951 | 19.0649 | *A. alba* | 68 | 42.2 | 576.3 | 544.0 | 30.0 | 24.9 |
| 112 | Jeleśnia | 254a | 49.5978 | 19.2445 | *A. alba* | 93 | 36.0 | 575.3 | 301.2 | 37.9 | 29.7 |
| 105 | Sucha | 50a | 49.8024 | 19.6118 | *A. alba* | 82 | 42.3 | 656.8 | 407.1 | 34.3 | 30.8 |
| 44 | Torzym | 116g | 52.3776 | 14.9313 | *L. decidua* | 27 | 24.5 | 260.1 | 875.0 | 18.4 | 19.5 |
| 99 | Bardo Śląskie | 99j | 50.4824 | 16.8077 | *L. decidua* | 68 | 37.5 | 416.2 | 525.8 | 29.6 | 23.5 |
| 75 | Lubsko | 3Aa | 51.8167 | 14.7704 | *L. decidua* | 34 | 21.2 | 236.8 | 548.7 | 22.0 | 20.6 |
| 85 | Golub-Dobrzyń | 269j | 53.1313 | 19.1653 | *L. decidua* | 76 | 26.9 | 421.2 | 306.8 | 32.9 | 31.3 |
| 59 | Warcino | 214d | 54.2420 | 16.9234 | *L. decidua* | 96 | 34.6 | 555.1 | 192.5 | 47.4 | 35.4 |
| 25 | Strzelce Krajeńskie | 133f | 52.9093 | 15.3744 | *L. decidua* | 46 | 28.8 | 362.1 | 312.1 | 33.9 | 26.1 |
| 87 | Kłobuck | 281n | 50.8620 | 18.9841 | *L. decidua* | 17 | 24.8 | 218.5 | 1441.4 | 14.5 | 14.9 |
| 63 | Złotów | 380c | 53.3094 | 17.2540 | *L. decidua* | 59 | 40.1 | 635.8 | 396.4 | 35.1 | 31.6 |
| 139 | Lwówek Śląski | 325n | 51.0640 | 15.4947 | *L. decidua* | 7 | 1.9 | 7.7 | 1650.0 | 4.6 | 4.8 |
| 120 | Ustroń | 114d | 49.7189 | 18.7176 | *L. decidua* | 120 | 28.9 | 466.5 | 170.0 | 45.7 | 33.9 |
| 36 | Gryfice | 755n | 53.9093 | 14.8819 | *L. decidua* | 88 | 38.4 | 615.4 | 373.3 | 35.6 | 33.1 |
| 54 | Gościno | 32a | 54.1808 | 15.7243 | *L. decidua* | 106 | 51.2 | 944.4 | 332.3 | 43.8 | 37.4 |
| 66 | Lipka | 315f | 53.4546 | 16.9039 | *A. glutinosa* | 23 | 28.6 | 289.5 | 1415.6 | 15.7 | 18.1 |
| 53 | Międzyzdroje | 451d | 53.8640 | 14.7227 | *A. glutinosa* | 33 | 35.6 | 417.9 | 1177.8 | 19.0 | 22.3 |
| 31 | Dąbrowa | 67d | 53.5197 | 18.5618 | *A. glutinosa* | 69 | 34.6 | 432.4 | 679.5 | 24.6 | 23.7 |
| 55 | Gościno | 66r | 54.1521 | 15.6794 | *A. glutinosa* | 46 | 37.4 | 457.4 | 680.7 | 25.8 | 24.6 |
| 19 | Międzychód | 48l | 52.6151 | 15.6931 | *A. glutinosa* | 62 | 42.9 | 597.0 | 661.9 | 27.8 | 27.7 |
| 20 | Bolewice | 676g | 52.3800 | 16.0312 | *A. glutinosa* | 49 | 39.5 | 507.9 | 963.0 | 22.4 | 24.8 |
| 50 | Nowogard | 82a | 53.7247 | 14.8949 | *A. glutinosa* | 21 | 14.5 | 105.3 | 909.1 | 14.0 | 13.3 |
| 102 | Namysłów | 191c | 51.0171 | 17.6315 | *A. glutinosa* | 37 | 35.8 | 417.2 | 883.3 | 22.4 | 22.9 |
| 135 | Szubin | 57p | 53.1257 | 17.3775 | *A. glutinosa* | 9 | 16.4 | 95.5 | 2925.0 | 8.2 | 10.4 |
| 16 | Jamy | 155b | 53.3674 | 18.5920 | *P. sylvestris* | 41 | 34.4 | 347.3 | 1437.5 | 17.0 | 19.0 |
| 67 | Wronki | 591c | 52.7002 | 16.1249 | *P. sylvestris* | 85 | 36.9 | 398.5 | 712.5 | 25.2 | 21.7 |
| 33 | Różańsko | 327a | 52.8489 | 14.8402 | *P. sylvestris* | 56 | 38.5 | 459.7 | 1010.1 | 21.6 | 23.6 |
| 56 | Gościno | 505f | 53.9220 | 15.5687 | *P. sylvestris* | 66 | 34.9 | 509.9 | 560.0 | 27.6 | 27.6 |
| 88 | Kłobuck | 196d | 50.9306 | 19.0930 | *P. sylvestris* | 34 | 36.9 | 336.2 | 1211.4 | 19.3 | 18.6 |
| 42 | Świebodzin | 243f | 52.3113 | 15.2646 | *P. sylvestris* | 56 | 43.9 | 576.6 | 960.0 | 23.6 | 26.6 |
| 47 | Goleniów | 163n | 53.6961 | 14.7978 | *P. sylvestris* | 78 | 37.1 | 527.3 | 372.6 | 34.9 | 28.8 |
| 74 | Lubsko | 205b | 51.7274 | 14.8599 | *P. sylvestris* | 36 | 27.1 | 171.9 | 3183.2 | 10.2 | 10.8 |
| 52 | Międzyzdroje | 128a | 53.8744 | 14.5229 | *P. sylvestris* | 94 | 34.0 | 421.8 | 287.2 | 38.0 | 28.3 |
| 62 | Złotów | 89a | 53.3892 | 17.2415 | *P. sylvestris* | 94 | 34.5 | 468.3 | 363.1 | 34.4 | 28.6 |
| 26 | Barlinek | 681l | 53.0173 | 15.2176 | *P. sylvestris* | 87 | 39.7 | 595.4 | 360.0 | 37.0 | 30.8 |
| 23 | Strzelce Krajeńskie | 82r | 52.9355 | 15.6840 | *P. sylvestris* | 26 | 28.4 | 235.8 | 1433.3 | 15.6 | 14.8 |
| 138 | Miradz | 220l | 52.5382 | 18.1946 | *P. sylvestris* | 17 | 29.0 | 177.9 | 3812.5 | 9.3 | 9.2 |
| 76 | Lipinki | 335b | 51.5952 | 15.1374 | *P. sylvestris* | 45 | 37.7 | 430.1 | 1180.0 | 19.8 | 21.4 |
| 136 | Szubin | 286g | 52.9425 | 17.8840 | *P. sylvestris* | 5 | 0.2 | 5.6 | 7753.6 | 3.0 | 1.3 |
| 57 | Sławno | 100b | 54.3281 | 16.7905 | *P. sylvestris* | 117 | 34.7 | 461.3 | 186.1 | 47.9 | 31.1 |
| 80 | Człuchów | 154f | 53.7265 | 17.3611 | *P. sylvestris* | 114 | 30.2 | 453.2 | 226.7 | 40.7 | 30.7 |
| 101 | Kup | 92c | 50.8495 | 17.8112 | *P. sylvestris* | 107 | 40.2 | 626.4 | 403.5 | 35.0 | 30.8 |
| 22 | Bolewice | 168d | 52.4868 | 15.9715 | *P. sylvestris* | 107 | 35.1 | 528.6 | 320.0 | 37.0 | 28.5 |
| 128 | Strzelce Opolskie | 270a | 50.5652 | 18.3247 | *P. sylvestris* | 16 | 31.7 | 176.9 | 6139.4 | 7.7 | 9.0 |
| 60 | Wałcz | 644g | 53.2556 | 16.3399 | *P. sylvestris* | 8 | 1.4 | 10.7 | 14125.0 | 2.3 | 1.8 |
| 40 | Wolsztyn | 116j | 52.1507 | 16.1413 | *P. sylvestris* | 25 | 25.9 | 169.5 | 3712.4 | 9.1 | 10.2 |
| 73 | Świdwin | 494a | 53.6999 | 15.9942 | *P. abies* | 98 | 49.7 | 779.3 | 271.4 | 47.9 | 35.3 |
| 46 | Trzebież | 122r | 53.6623 | 14.4121 | *P. abies* | 39 | 31.5 | 345.7 | 595.7 | 25.5 | 21.7 |
| 169 | Miastko | 208b | 54.0087 | 16.8530 | *P. abies* | 81 | 44.2 | 598.1 | 248.9 | 47.1 | 35.1 |
| 28 | Barlinek | 244f | 52.8882 | 15.0922 | *P. abies* | 45 | 38.3 | 408.0 | 1089.0 | 20.7 | 20.2 |
| 103 | Namysłów | 66f | 51.0485 | 17.6523 | *P. abies* | 15 | 14.0 | 75.4 | 3448.3 | 7.9 | 7.0 |
| 98 | Bardo Śląskie | 141g | 50.4855 | 16.7698 | *P. abies* | 68 | 50.6 | 855.7 | 555.0 | 33.3 | 32.3 |
| 106 | Sucha | 416a | 49.6174 | 19.5754 | *P. abies* | 15 | 12.8 | 61.0 | 12095.2 | 3.8 | 5.3 |
| 81 | Człuchów | 314d | 53.5358 | 17.1376 | *P. abies* | 10 | 4.8 | 22.1 | 4190.5 | 4.8 | 3.5 |
| 126 | Ujsoły | 191b | 49.4316 | 19.1484 | *P. abies* | 60 | 48.8 | 701.3 | 816.7 | 26.9 | 27.7 |
| 71 | Warcino | 187f | 54.2016 | 16.7205 | *P. abies* | 66 | 44.3 | 598.5 | 462.5 | 34.3 | 27.7 |
| 107 | Sucha | 418a | 49.6128 | 19.5767 | *P. abies* | 117 | 44.0 | 571.9 | 605.0 | 29.6 | 24.6 |
| 110 | Jeleśnia | 211b | 49.5419 | 19.2499 | *P. abies* | 28 | 32.8 | 325.9 | 1053.0 | 19.2 | 18.3 |
| 78 | Durowo | 125j | 52.8248 | 17.1674 | *P. abies* | 57 | 42.6 | 568.6 | 857.1 | 24.5 | 24.1 |
| 51 | Międzyzdroje | 82i | 53.8906 | 14.4901 | *P. abies* | 78 | 40.5 | 566.3 | 456.0 | 32.8 | 30.8 |
| 38 | Gryfice | 378l | 53.8779 | 15.0519 | *P. abies* | 88 | 42.8 | 585.1 | 376.7 | 37.4 | 31.0 |
| 117 | Wisła | 67b | 49.5752 | 18.9253 | *P. abies* | 71 | 47.4 | 745.9 | 580.0 | 31.4 | 30.9 |
| 140 | Świeradów | 214b | 51.0565 | 15.1965 | *P. abies* | 35 | 33.3 | 361.0 | 1856.0 | 14.5 | 18.9 |
| 118 | Ustroń | 4c | 49.7347 | 18.8409 | *P. abies* | 85 | 49.3 | 624.0 | 847.2 | 26.3 | 24.3 |
| 123 | Torzym | 47g | 52.3667 | 14.9967 | *P. abies* | 27 | 27.3 | 218.6 | 2436.8 | 11.6 | 12.7 |
| 142 | Lwówek Śląski | 188a | 51.1261 | 15.7226 | *P. abies* | 7 | 1.6 | 6.6 | 3011.4 | 3.9 | 2.7 |
| 82 | Gniewkowo | 444h | 52.8920 | 18.6806 | *P. abies* | 109 | 30.5 | 485.7 | 320.0 | 34.4 | 30.0 |
| 114 | Ujsoły | 220a | 49.4196 | 19.1419 | *P. abies* | 95 | 36.4 | 533.3 | 250.0 | 42.4 | 34.2 |
| 69 | Polanów | 492b | 54.0763 | 16.6868 | *P. abies* | 113 | 35.2 | 547.4 | 139.2 | 55.3 | 35.7 |
| 92 | Zdroje | 466b | 50.4158 | 16.3830 | *P. abies* | 107 | 22.1 | 353.0 | 196.3 | 37.4 | 29.9 |
| 97 | Świdnica | 241b | 50.6605 | 16.5536 | *P. abies* | 46 | 54.9 | 655.3 | 924.2 | 26.9 | 24.4 |

**Table S3.** Parameters of mixed effects ANCOVA explaining impacts of studied species and sample mass on the difference in dry mass between two drying temperatures (D)

| **Component** | **Term** | **Estimate** | **SE** | **t** | **Pr(>\|t\|)** |
| --- | --- | --- | --- | --- | --- |
| bark | (Intercept) | 0.01270 | 0.00089 | 14.29200 | <0.001 |
| bark | species=*F. sylvatica* | 0.00108 | 0.00123 | 0.87500 | 0.3860 |
| bark | species=*Q. robur* | 0.00207 | 0.00125 | 1.66000 | 0.1020 |
| bark | species=*A. alba* | 0.00321 | 0.00129 | 2.48500 | 0.0172 |
| bark | species=*L. decidua* | 0.00443 | 0.00125 | 3.54900 | 0.0008 |
| bark | species=*A. glutinosa* | 0.00283 | 0.00126 | 2.23700 | 0.0311 |
| bark | species=*P. sylvestris* | 0.00562 | 0.00124 | 4.52300 | <0.0001 |
| bark | species=*P. abies* | 0.00564 | 0.00121 | 4.68000 | <0.0001 |
| bark | sample mass (105°C) | 0.00001 | 0.00000 | 3.00500 | 0.0030 |
| bark | random effect (plot) | SD= | 0.00117 | - | - |
| bark | random effect (residuals) | SD= | 0.00410 | - | - |
| branches | (Intercept) | 0.01493 | 0.00067 | 22.14830 | <0.001 |
| branches | species=*F. sylvatica* | 0.00112 | 0.00043 | 2.58677 | 0.5691 |
| branches | species=*Q. robur* | 0.00403 | 0.00044 | 9.25042 | 0.1572 |
| branches | species=*A. alba* | 0.00217 | 0.00043 | 5.04526 | 0.0613 |
| branches | species=*L. decidua* | 0.00527 | 0.00043 | 12.35538 | 0.0021 |
| branches | species=*A. glutinosa* | 0.00129 | 0.00043 | 3.03901 | 0.1491 |
| branches | species=*P. sylvestris* | 0.00314 | 0.00043 | 7.27690 | 0.0001 |
| branches | species=*P. abies* | 0.00462 | 0.00042 | 10.96356 | <0.0001 |
| branches | sample mass (105°C) | <0.00001 | <0.00001 | -0.06172 | 0.0168 |
| branches | random effect (plot) | SD= | 0.00041 | - | - |
| branches | random effect (residuals) | SD= | 0.00133 | - | - |
| foliage | (Intercept) | 0.02763 | 0.00092 | 29.90800 | <0.001 |
| foliage | species=*F. sylvatica* | -0.00119 | 0.00088 | -1.34000 | 0.1867 |
| foliage | species=*Q. robur* | -0.00030 | 0.00092 | -0.32700 | 0.7458 |
| foliage | species=*A. alba* | -0.01843 | 0.00133 | -13.90000 | <0.0001 |
| foliage | species=*L. decidua* | -0.01336 | 0.00089 | -15.01900 | <0.0001 |
| foliage | species=*A. glutinosa* | 0.00464 | 0.00090 | 5.12300 | <0.0001 |
| foliage | species=*P. sylvestris* | -0.01589 | 0.00091 | -17.52400 | <0.0001 |
| foliage | species=*P. abies* | -0.01797 | 0.00138 | -12.99500 | <0.0001 |
| foliage | sample mass (105°C) | 0.00001 | 0.00001 | 2.39200 | 0.0177 |
| foliage | random effect (plot) | SD= | 0.00091 | - | - |
| foliage | random effect (residuals) | SD= | 0.00280 | - | - |
| wood | (Intercept) | 0.00783 | 0.00026 | 30.67803 | <0.0001 |
| wood | species=*F. sylvatica* | 0.00022 | 0.00034 | 0.63644 | 0.5266 |
| wood | species=*Q. robur* | 0.00384 | 0.00033 | 11.47788 | <0.0001 |
| wood | species=*A. alba* | 0.00426 | 0.00035 | 12.27859 | <0.0001 |
| wood | species=*L. decidua* | 0.00189 | 0.00034 | 5.53243 | <0.0001 |
| wood | species=*A. glutinosa* | -0.00007 | 0.00036 | -0.20524 | 0.8383 |
| wood | species=*P. sylvestris* | 0.00218 | 0.00033 | 6.52300 | <0.0001 |
| wood | species=*P. abies* | 0.00309 | 0.00033 | 9.23512 | <0.0001 |
| wood | sample mass (105°C) | <0.00001 | <0.00001 | -1.77723 | 0.0761 |
| wood | random effect (plot) | SD= | 0.00039 | - | - |
| wood | random effect (residuals) | SD= | 0.00189 | - | - |

**Table S4.** Models used for biomass calculation at tree and stand level

| **Level** | **Species** | **Formula** | **a** | **SE** | **b** | **SE** | **c** | **SE** | **RMSE** | **R^2^** | **n** | **Source** | **Remarks** |
| --- | --- | --- | --- | --- | --- | --- | --- | --- | --- | --- | --- | --- | --- |
| Stand | *F. sylvatica* | y=a*V^b | 0.5297 | 0.0580 | 1.0041 | 0.0173 | - | - | 1.30 | 0.999 | 12 | ^1^ | for lowlands stands only |
| Tree | *F. sylvatica* | log(y)=a+b*log(DBH)+c*log(H) | -3.6560 | 0.0603 | 1.8349 | 0.0371 | 1.0994 | 0.0464 | 8.54 | 0.998 | 96 | ^1^ | for backward transformation correction factor=1.0058 |
| Stand | *L. decidua* | y=a*V^b | 0.2098 | 0.0299 | 1.1050 | 0.0221 | - | - | 0.71 | 0.997 | 12 | ^2^ | - |
| Tree | *L. decidua* | y=a*DBH^b | 0.1397 | 0.0366 | 2.3588 | 0.0689 | - | - | 42.16 | 0.961 | 96 | ^2^ | - |
| Stand | *P. sylvestris* | y=a*V^b | 0.2260 | 0.0153 | 1.0926 | 0.0112 | - | - | 13.74 | 0.985 | 120 | ^3^ | - |
| Tree | *P. sylvestris* | y=a*DBH^b | 0.1899 | 0.0168 | 2.2082 | 0.0235 | - | - | 6.34 | 0.967 | 549 | ^3^ | - |

RMSE – root mean squared error, R^2^ – coefficient of determination, n – sample size

**References**

1. Jagodziński, A. M., Dyderski, M. K. & Horodecki, P. Differences in biomass production and carbon sequestration between highland and lowland stands of *Picea abies* (L.) H. Karst. and *Fagus sylvatica* L. *Forest Ecology and Management* **474**, 118329 (2020).

2. Jagodziński, A. M., Dyderski, M. K., Gęsikiewicz, K. & Horodecki, P. Tree- and Stand-Level Biomass Estimation in a *Larix decidua* Mill. Chronosequence. *Forests* **9**, 587 (2018).

3. Jagodziński, A. M., Dyderski, M. K., Gęsikiewicz, K. & Horodecki, P. Effects of stand features on aboveground biomass and biomass conversion and expansion factors based on a *Pinus sylvestris* L. chronosequence in Western Poland. *European Journal of Forest Research* **138**, 673–683 (2019).

**Table S5.** Database of simulated tree and stand level biomass at 75°C and 105°C. We used H only in models of *F. sylvatica* biomass, due to model requirements. For models used – see **Table S4**

| **Level** | **Species** | **DBH or V**  **[cm for the tree or m^3^ ha^-1^ for the stand level]** | **H [m]** | **Stem biomass**  **in 75°C**  **[tree level - kg, stand level - Mg ha^-1^]** | **Stem biomass**  **in 105°C**  **[tree level - kg,**  **stand level - Mg ha^-1^]** | **Difference**  **[tree level - kg, stand level - Mg ha^-1^]** |
| --- | --- | --- | --- | --- | --- | --- |
| Tree | *P. sylvestris* | 10 |  | 30.671 | 30.329 | 0.342 |
| Tree | *P. sylvestris* | 11 |  | 37.855 | 37.433 | 0.422 |
| Tree | *P. sylvestris* | 12 |  | 45.875 | 45.363 | 0.511 |
| Tree | *P. sylvestris* | 13 |  | 54.744 | 54.134 | 0.610 |
| Tree | *P. sylvestris* | 14 |  | 64.477 | 63.758 | 0.719 |
| Tree | *P. sylvestris* | 15 |  | 75.088 | 74.251 | 0.837 |
| Tree | *P. sylvestris* | 16 |  | 86.589 | 85.624 | 0.965 |
| Tree | *P. sylvestris* | 17 |  | 98.992 | 97.889 | 1.103 |
| Tree | *P. sylvestris* | 18 |  | 112.310 | 111.058 | 1.252 |
| Tree | *P. sylvestris* | 19 |  | 126.552 | 125.141 | 1.411 |
| Tree | *P. sylvestris* | 20 |  | 141.729 | 140.149 | 1.580 |
| Tree | *P. sylvestris* | 21 |  | 157.852 | 156.092 | 1.760 |
| Tree | *P. sylvestris* | 22 |  | 174.929 | 172.979 | 1.950 |
| Tree | *P. sylvestris* | 23 |  | 192.971 | 190.820 | 2.151 |
| Tree | *P. sylvestris* | 24 |  | 211.986 | 209.623 | 2.363 |
| Tree | *P. sylvestris* | 25 |  | 231.983 | 229.397 | 2.586 |
| Tree | *P. sylvestris* | 26 |  | 252.970 | 250.150 | 2.820 |
| Tree | *P. sylvestris* | 27 |  | 274.956 | 271.891 | 3.065 |
| Tree | *P. sylvestris* | 28 |  | 297.947 | 294.626 | 3.321 |
| Tree | *P. sylvestris* | 29 |  | 321.953 | 318.364 | 3.589 |
| Tree | *P. sylvestris* | 30 |  | 346.980 | 343.112 | 3.868 |
| Tree | *P. sylvestris* | 31 |  | 373.035 | 368.877 | 4.158 |
| Tree | *P. sylvestris* | 32 |  | 400.126 | 395.666 | 4.460 |
| Tree | *P. sylvestris* | 33 |  | 428.260 | 423.486 | 4.774 |
| Tree | *P. sylvestris* | 34 |  | 457.443 | 452.344 | 5.099 |
| Tree | *P. sylvestris* | 35 |  | 487.681 | 482.245 | 5.436 |
| Tree | *P. sylvestris* | 36 |  | 518.982 | 513.197 | 5.785 |
| Tree | *P. sylvestris* | 37 |  | 551.351 | 545.205 | 6.146 |
| Tree | *P. sylvestris* | 38 |  | 584.794 | 578.276 | 6.519 |
| Tree | *P. sylvestris* | 39 |  | 619.318 | 612.415 | 6.903 |
| Tree | *P. sylvestris* | 40 |  | 654.928 | 647.628 | 7.300 |
| Tree | *P. sylvestris* | 41 |  | 691.631 | 683.921 | 7.709 |
| Tree | *P. sylvestris* | 42 |  | 729.431 | 721.300 | 8.131 |
| Tree | *P. sylvestris* | 43 |  | 768.334 | 759.769 | 8.564 |
| Tree | *P. sylvestris* | 44 |  | 808.346 | 799.335 | 9.010 |
| Tree | *P. sylvestris* | 45 |  | 849.472 | 840.003 | 9.469 |
| Tree | *P. sylvestris* | 46 |  | 891.717 | 881.777 | 9.940 |
| Tree | *P. sylvestris* | 47 |  | 935.086 | 924.663 | 10.423 |
| Tree | *P. sylvestris* | 48 |  | 979.584 | 968.665 | 10.919 |
| Tree | *P. sylvestris* | 49 |  | 1025.217 | 1013.789 | 11.428 |
| Tree | *P. sylvestris* | 50 |  | 1071.989 | 1060.040 | 11.949 |
| Tree | *F. sylvatica* | 10 | 10 | 22.337 | 22.142 | 0.195 |
| Tree | *F. sylvatica* | 11 | 10 | 26.606 | 26.374 | 0.233 |
| Tree | *F. sylvatica* | 12 | 10 | 31.212 | 30.939 | 0.273 |
| Tree | *F. sylvatica* | 13 | 10 | 36.150 | 35.834 | 0.316 |
| Tree | *F. sylvatica* | 14 | 10 | 41.415 | 41.053 | 0.362 |
| Tree | *F. sylvatica* | 15 | 10 | 47.005 | 46.594 | 0.411 |
| Tree | *F. sylvatica* | 16 | 10 | 52.914 | 52.451 | 0.463 |
| Tree | *F. sylvatica* | 17 | 10 | 59.140 | 58.623 | 0.517 |
| Tree | *F. sylvatica* | 18 | 10 | 65.680 | 65.105 | 0.574 |
| Tree | *F. sylvatica* | 19 | 10 | 72.530 | 71.896 | 0.634 |
| Tree | *F. sylvatica* | 20 | 10 | 79.688 | 78.991 | 0.697 |
| Tree | *F. sylvatica* | 21 | 15 | 136.102 | 134.913 | 1.190 |
| Tree | *F. sylvatica* | 22 | 15 | 148.230 | 146.935 | 1.296 |
| Tree | *F. sylvatica* | 23 | 15 | 160.827 | 159.422 | 1.406 |
| Tree | *F. sylvatica* | 24 | 15 | 173.890 | 172.370 | 1.520 |
| Tree | *F. sylvatica* | 25 | 15 | 187.416 | 185.777 | 1.638 |
| Tree | *F. sylvatica* | 26 | 15 | 201.400 | 199.640 | 1.760 |
| Tree | *F. sylvatica* | 27 | 15 | 215.842 | 213.955 | 1.887 |
| Tree | *F. sylvatica* | 28 | 15 | 230.736 | 228.719 | 2.017 |
| Tree | *F. sylvatica* | 29 | 15 | 246.082 | 243.931 | 2.151 |
| Tree | *F. sylvatica* | 30 | 20 | 359.297 | 356.156 | 3.141 |
| Tree | *F. sylvatica* | 31 | 20 | 381.578 | 378.242 | 3.335 |
| Tree | *F. sylvatica* | 32 | 20 | 404.467 | 400.931 | 3.535 |
| Tree | *F. sylvatica* | 33 | 20 | 427.961 | 424.221 | 3.741 |
| Tree | *F. sylvatica* | 34 | 20 | 452.058 | 448.106 | 3.951 |
| Tree | *F. sylvatica* | 35 | 20 | 476.753 | 472.586 | 4.167 |
| Tree | *F. sylvatica* | 36 | 20 | 502.045 | 497.657 | 4.388 |
| Tree | *F. sylvatica* | 37 | 20 | 527.930 | 523.316 | 4.615 |
| Tree | *F. sylvatica* | 38 | 20 | 554.406 | 549.560 | 4.846 |
| Tree | *F. sylvatica* | 39 | 20 | 581.471 | 576.388 | 5.083 |
| Tree | *F. sylvatica* | 40 | 25 | 778.478 | 771.673 | 6.805 |
| Tree | *F. sylvatica* | 41 | 25 | 814.561 | 807.440 | 7.120 |
| Tree | *F. sylvatica* | 42 | 25 | 851.386 | 843.944 | 7.442 |
| Tree | *F. sylvatica* | 43 | 25 | 888.950 | 881.180 | 7.770 |
| Tree | *F. sylvatica* | 44 | 25 | 927.252 | 919.146 | 8.105 |
| Tree | *F. sylvatica* | 45 | 30 | 1180.749 | 1170.428 | 10.321 |
| Tree | *F. sylvatica* | 46 | 30 | 1229.341 | 1218.595 | 10.746 |
| Tree | *F. sylvatica* | 47 | 30 | 1278.823 | 1267.645 | 11.178 |
| Tree | *F. sylvatica* | 48 | 30 | 1329.192 | 1317.573 | 11.619 |
| Tree | *F. sylvatica* | 49 | 30 | 1380.444 | 1368.378 | 12.067 |
| Tree | *F. sylvatica* | 50 | 30 | 1432.578 | 1420.055 | 12.522 |
| Tree | *L. decidua* | 10 |  | 31.915 | 31.570 | 0.345 |
| Tree | *L. decidua* | 11 |  | 39.961 | 39.529 | 0.432 |
| Tree | *L. decidua* | 12 |  | 49.065 | 48.535 | 0.530 |
| Tree | *L. decidua* | 13 |  | 59.261 | 58.620 | 0.640 |
| Tree | *L. decidua* | 14 |  | 70.580 | 69.818 | 0.763 |
| Tree | *L. decidua* | 15 |  | 83.054 | 82.157 | 0.897 |
| Tree | *L. decidua* | 16 |  | 96.711 | 95.666 | 1.045 |
| Tree | *L. decidua* | 17 |  | 111.578 | 110.373 | 1.206 |
| Tree | *L. decidua* | 18 |  | 127.683 | 126.304 | 1.380 |
| Tree | *L. decidua* | 19 |  | 145.051 | 143.484 | 1.567 |
| Tree | *L. decidua* | 20 |  | 163.707 | 161.938 | 1.769 |
| Tree | *L. decidua* | 21 |  | 183.674 | 181.689 | 1.984 |
| Tree | *L. decidua* | 22 |  | 204.976 | 202.761 | 2.215 |
| Tree | *L. decidua* | 23 |  | 227.636 | 225.176 | 2.459 |
| Tree | *L. decidua* | 24 |  | 251.674 | 248.955 | 2.719 |
| Tree | *L. decidua* | 25 |  | 277.113 | 274.119 | 2.994 |
| Tree | *L. decidua* | 26 |  | 303.973 | 300.689 | 3.284 |
| Tree | *L. decidua* | 27 |  | 332.275 | 328.685 | 3.590 |
| Tree | *L. decidua* | 28 |  | 362.037 | 358.125 | 3.911 |
| Tree | *L. decidua* | 29 |  | 393.279 | 389.030 | 4.249 |
| Tree | *L. decidua* | 30 |  | 426.020 | 421.417 | 4.603 |
| Tree | *L. decidua* | 31 |  | 460.278 | 455.305 | 4.973 |
| Tree | *L. decidua* | 32 |  | 496.071 | 490.712 | 5.360 |
| Tree | *L. decidua* | 33 |  | 533.417 | 527.654 | 5.763 |
| Tree | *L. decidua* | 34 |  | 572.333 | 566.150 | 6.184 |
| Tree | *L. decidua* | 35 |  | 612.836 | 606.215 | 6.621 |
| Tree | *L. decidua* | 36 |  | 654.942 | 647.866 | 7.076 |
| Tree | *L. decidua* | 37 |  | 698.668 | 691.119 | 7.548 |
| Tree | *L. decidua* | 38 |  | 744.029 | 735.991 | 8.039 |
| Tree | *L. decidua* | 39 |  | 791.042 | 782.496 | 8.547 |
| Tree | *L. decidua* | 40 |  | 839.722 | 830.650 | 9.072 |
| Tree | *L. decidua* | 41 |  | 890.084 | 880.467 | 9.617 |
| Tree | *L. decidua* | 42 |  | 942.143 | 931.964 | 10.179 |
| Tree | *L. decidua* | 43 |  | 995.914 | 985.154 | 10.760 |
| Tree | *L. decidua* | 44 |  | 1051.411 | 1040.052 | 11.360 |
| Tree | *L. decidua* | 45 |  | 1108.649 | 1096.671 | 11.978 |
| Tree | *L. decidua* | 46 |  | 1167.642 | 1155.026 | 12.615 |
| Tree | *L. decidua* | 47 |  | 1228.403 | 1215.131 | 13.272 |
| Tree | *L. decidua* | 48 |  | 1290.946 | 1276.999 | 13.948 |
| Tree | *L. decidua* | 49 |  | 1355.286 | 1340.643 | 14.643 |
| Tree | *L. decidua* | 50 |  | 1421.435 | 1406.077 | 15.357 |
| Stand | *F. sylvatica* | 10 |  | 5.347 | 5.301 | 0.047 |
| Stand | *F. sylvatica* | 20 |  | 10.725 | 10.631 | 0.094 |
| Stand | *F. sylvatica* | 30 |  | 16.114 | 15.973 | 0.141 |
| Stand | *F. sylvatica* | 40 |  | 21.511 | 21.323 | 0.188 |
| Stand | *F. sylvatica* | 50 |  | 26.913 | 26.678 | 0.235 |
| Stand | *F. sylvatica* | 60 |  | 32.320 | 32.038 | 0.283 |
| Stand | *F. sylvatica* | 70 |  | 37.731 | 37.401 | 0.330 |
| Stand | *F. sylvatica* | 80 |  | 43.144 | 42.767 | 0.377 |
| Stand | *F. sylvatica* | 90 |  | 48.561 | 48.136 | 0.424 |
| Stand | *F. sylvatica* | 100 |  | 53.980 | 53.508 | 0.472 |
| Stand | *F. sylvatica* | 110 |  | 59.401 | 58.882 | 0.519 |
| Stand | *F. sylvatica* | 120 |  | 64.824 | 64.257 | 0.567 |
| Stand | *F. sylvatica* | 130 |  | 70.249 | 69.635 | 0.614 |
| Stand | *F. sylvatica* | 140 |  | 75.676 | 75.014 | 0.661 |
| Stand | *F. sylvatica* | 150 |  | 81.104 | 80.395 | 0.709 |
| Stand | *F. sylvatica* | 160 |  | 86.534 | 85.778 | 0.756 |
| Stand | *F. sylvatica* | 170 |  | 91.965 | 91.161 | 0.804 |
| Stand | *F. sylvatica* | 180 |  | 97.398 | 96.546 | 0.851 |
| Stand | *F. sylvatica* | 190 |  | 102.832 | 101.933 | 0.899 |
| Stand | *F. sylvatica* | 200 |  | 108.267 | 107.320 | 0.946 |
| Stand | *F. sylvatica* | 210 |  | 113.703 | 112.709 | 0.994 |
| Stand | *F. sylvatica* | 220 |  | 119.140 | 118.098 | 1.041 |
| Stand | *F. sylvatica* | 230 |  | 124.578 | 123.489 | 1.089 |
| Stand | *F. sylvatica* | 240 |  | 130.017 | 128.880 | 1.136 |
| Stand | *F. sylvatica* | 250 |  | 135.457 | 134.273 | 1.184 |
| Stand | *F. sylvatica* | 260 |  | 140.898 | 139.666 | 1.232 |
| Stand | *F. sylvatica* | 270 |  | 146.340 | 145.061 | 1.279 |
| Stand | *F. sylvatica* | 280 |  | 151.782 | 150.456 | 1.327 |
| Stand | *F. sylvatica* | 290 |  | 157.226 | 155.851 | 1.374 |
| Stand | *F. sylvatica* | 300 |  | 162.670 | 161.248 | 1.422 |
| Stand | *L. decidua* | 10 |  | 2.672 | 2.643 | 0.029 |
| Stand | *L. decidua* | 20 |  | 5.747 | 5.685 | 0.062 |
| Stand | *L. decidua* | 30 |  | 8.995 | 8.898 | 0.097 |
| Stand | *L. decidua* | 40 |  | 12.362 | 12.228 | 0.134 |
| Stand | *L. decidua* | 50 |  | 15.819 | 15.648 | 0.171 |
| Stand | *L. decidua* | 60 |  | 19.349 | 19.140 | 0.209 |
| Stand | *L. decidua* | 70 |  | 22.942 | 22.695 | 0.248 |
| Stand | *L. decidua* | 80 |  | 26.590 | 26.303 | 0.287 |
| Stand | *L. decidua* | 90 |  | 30.286 | 29.959 | 0.327 |
| Stand | *L. decidua* | 100 |  | 34.026 | 33.658 | 0.368 |
| Stand | *L. decidua* | 110 |  | 37.805 | 37.396 | 0.408 |
| Stand | *L. decidua* | 120 |  | 41.620 | 41.170 | 0.450 |
| Stand | *L. decidua* | 130 |  | 45.469 | 44.977 | 0.491 |
| Stand | *L. decidua* | 140 |  | 49.349 | 48.816 | 0.533 |
| Stand | *L. decidua* | 150 |  | 53.258 | 52.683 | 0.575 |
| Stand | *L. decidua* | 160 |  | 57.195 | 56.577 | 0.618 |
| Stand | *L. decidua* | 170 |  | 61.158 | 60.497 | 0.661 |
| Stand | *L. decidua* | 180 |  | 65.145 | 64.441 | 0.704 |
| Stand | *L. decidua* | 190 |  | 69.156 | 68.409 | 0.747 |
| Stand | *L. decidua* | 200 |  | 73.189 | 72.398 | 0.791 |
| Stand | *L. decidua* | 210 |  | 77.243 | 76.408 | 0.835 |
| Stand | *L. decidua* | 220 |  | 81.317 | 80.439 | 0.879 |
| Stand | *L. decidua* | 230 |  | 85.411 | 84.488 | 0.923 |
| Stand | *L. decidua* | 240 |  | 89.524 | 88.557 | 0.967 |
| Stand | *L. decidua* | 250 |  | 93.655 | 92.643 | 1.012 |
| Stand | *L. decidua* | 260 |  | 97.803 | 96.746 | 1.057 |
| Stand | *L. decidua* | 270 |  | 101.968 | 100.866 | 1.102 |
| Stand | *L. decidua* | 280 |  | 106.149 | 105.002 | 1.147 |
| Stand | *L. decidua* | 290 |  | 110.346 | 109.153 | 1.192 |
| Stand | *L. decidua* | 300 |  | 114.558 | 113.320 | 1.238 |
| Stand | *P. sylvestris* | 10 |  | 2.797 | 2.766 | 0.031 |
| Stand | *P. sylvestris* | 20 |  | 5.965 | 5.899 | 0.066 |
| Stand | *P. sylvestris* | 30 |  | 9.290 | 9.186 | 0.104 |
| Stand | *P. sylvestris* | 40 |  | 12.721 | 12.579 | 0.142 |
| Stand | *P. sylvestris* | 50 |  | 16.233 | 16.052 | 0.181 |
| Stand | *P. sylvestris* | 60 |  | 19.811 | 19.591 | 0.221 |
| Stand | *P. sylvestris* | 70 |  | 23.446 | 23.184 | 0.261 |
| Stand | *P. sylvestris* | 80 |  | 27.128 | 26.826 | 0.302 |
| Stand | *P. sylvestris* | 90 |  | 30.854 | 30.510 | 0.344 |
| Stand | *P. sylvestris* | 100 |  | 34.619 | 34.233 | 0.386 |
| Stand | *P. sylvestris* | 110 |  | 38.418 | 37.990 | 0.428 |
| Stand | *P. sylvestris* | 120 |  | 42.250 | 41.779 | 0.471 |
| Stand | *P. sylvestris* | 130 |  | 46.111 | 45.597 | 0.514 |
| Stand | *P. sylvestris* | 140 |  | 50.000 | 49.442 | 0.557 |
| Stand | *P. sylvestris* | 150 |  | 53.915 | 53.314 | 0.601 |
| Stand | *P. sylvestris* | 160 |  | 57.854 | 57.209 | 0.645 |
| Stand | *P. sylvestris* | 170 |  | 61.815 | 61.126 | 0.689 |
| Stand | *P. sylvestris* | 180 |  | 65.799 | 65.066 | 0.733 |
| Stand | *P. sylvestris* | 190 |  | 69.803 | 69.025 | 0.778 |
| Stand | *P. sylvestris* | 200 |  | 73.827 | 73.004 | 0.823 |
| Stand | *P. sylvestris* | 210 |  | 77.869 | 77.001 | 0.868 |
| Stand | *P. sylvestris* | 220 |  | 81.929 | 81.016 | 0.913 |
| Stand | *P. sylvestris* | 230 |  | 86.007 | 85.048 | 0.959 |
| Stand | *P. sylvestris* | 240 |  | 90.101 | 89.096 | 1.004 |
| Stand | *P. sylvestris* | 250 |  | 94.210 | 93.160 | 1.050 |
| Stand | *P. sylvestris* | 260 |  | 98.335 | 97.239 | 1.096 |
| Stand | *P. sylvestris* | 270 |  | 102.475 | 101.332 | 1.142 |
| Stand | *P. sylvestris* | 280 |  | 106.629 | 105.440 | 1.189 |
| Stand | *P. sylvestris* | 290 |  | 110.796 | 109.561 | 1.235 |
| Stand | *P. sylvestris* | 300 |  | 114.977 | 113.695 | 1.282 |
